# MicroRNA profiles of dry secretions through the first three weeks of the dry period from Holstein cows

**DOI:** 10.1038/s41598-019-56193-5

**Published:** 2019-12-23

**Authors:** Ellie J. Putz, Austin M. Putz, Hyeongseon Jeon, John D. Lippolis, Hao Ma, Timothy A. Reinhardt, Eduardo Casas

**Affiliations:** 1 Ruminant Diseases and Immunology Research Unit, USDA Agriculture Research Service, National Animal Disease Center, Ames, IA USA; 2 Oak Ridge Institute for Science and Education, Oak Ridge Associated Universities, Oak Ridge, TN USA; 3 Animal Breeding and Genetics, Iowa State University, Ames, IA USA; 4 Department of Statistics, Iowa State University, Ames, IA USA

**Keywords:** RNA sequencing, Agricultural genetics

## Abstract

In dairy cows, the period from the end of lactation through the dry period and into the transition period, requires vast physiological and immunological changes critical to mammary health. The dry period is important to the success of the next lactation and intramammary infections during the dry period will adversely alter mammary function, health and milk production for the subsequent lactation. MicroRNAs (miRNAs) are small non-coding RNAs that can post transcriptionally regulate gene expression. We sought to characterize the miRNA profile in dry secretions from the last day of lactation to 3, 10, and 21 days post dry-off. We identified 816 known and 80 novel miRNAs. We found 46 miRNAs whose expression significantly changed (q-value < 0.05) over the first three weeks of dry-off. Additionally, we examined the slopes of random regression models of log transformed normalized counts and cross analyzed the 46 significantly upregulated and downregulated miRNAs. These miRNAs were found to be associated with important components of pregnancy, lactation, as well as inflammation and disease. Detailing the miRNA profile of dry secretions through the dry-off period provides insight into the biology at work, possible means of regulation, components of resistance and/or susceptibility, and outlets for targeted therapy development.

## Introduction

MicroRNAs (miRNAs) are small non-coding RNAs that can modify gene expression at the post transcription level^1^. To date, miRNAs have been associated with regulation of biological processes such as immune response, development, cancer, and specific disease and pathogen associations^2,3^. Attention has been brought to miRNAs as potential biomarkers of traits or phenotypes of interest, particularly in the context of disease pathogenesis, detection, and prediction. While miRNAs were initially described in humans, miRNAs are becoming increasingly characterized in livestock veterinary models including classification within the porcine, avian, and bovine families^2,4,5^.

Serum sourced miRNAs from beef calves were shown to be associated with the results of Enzyme Linked Immunosorbent Assay (ELISA) antibody testing of *Mycoplasma bovis*^6^. Differential expression of serum sourced bovine miRNAs were also identified in calves challenged with Bovine Viral Diarrhea Virus^7^. In addition to common cow diseases, the dairy cow provides a lactation/mastitis model to examine miRNA expression. For example, miRNA differentially express with stage of lactation^8^. In mastitis challenge models, milk CD14+ cells from cows infected with *Streptococcus uberis* or exosomes from *Staphylococcus aureus* infected cows showed differential miRNA expression compared to their respective controls^9,10^. Understanding these changes could have important implications in the detection and understanding the immune response of mastitis infections^10^.

Clinical mastitis infections postpartum pose an adverse health and economic impact on the cow. Dry period infection dynamics and bacteriology play a large role in causative clinical mastitis in the subsequent lactation^11^. Cows are generally dried off approximately 60 days before their calving due date^12,13^. During this dry period the mammary gland replenishes epithelial cell content and optimizes the mammary environment for subsequent milk production^14,15^. The periparturient period, spanning from roughly three weeks before to three weeks after calving, is one of great energy demand and immune suppression for dairy cows^16,17^. Increased stress, metabolic demand, and immune suppression including the reduced function of critical immune cells such as neutrophils, contribute to the high incidence of periparturient disease^18,19^. High on the lists of periparturient diseases are uterine infections and mastitis which have large consequences for the economic and health potential of the animal^19,20^. Lactation phase transitions represent massive physiologically and immunological changes for lactating mammals. Information that provides insight into the biology of the mammary gland during this sensitive time may ultimately contribute to better awareness of animal susceptibility/resistance, therapy development for disease treatment or prevention, and/or alternative health and immunological components.

Investigating miRNA profiles has the potential to identify uncharacterized immune regulators and identify trends and profiles of miRNA expression that may provide insight into the biological processes they are involved in. The role of miRNAs as predictive markers associated with future health or production phenotypes has also not been dismissed. The goal of this study was to profile the miRNAs in mammary secretions during the transition from lactation into day 21 of the dry period.

## Methods

### Experimental design

All protocols and animal procedures were approved by the Animal Care and Use Committee (ACUC) and follow the United States Department of Agriculture guide to Large Animals and the Animal Welfare Act. All animals were maintained and cared for at the National Animal Disease Center (NADC). Six multi-parity Holsteins, confirmed pregnant were utilized for this study. Dry secretion samples were taken on days 0, 3, 10 and 21 days of dry-off. Cows averaged 353.8 ± 5.8 days in milk at the start of the experiment and were all producing below 14 kg of milk at the last day of lactation (day 0).

### Dry-off & dry secretion collection

Approximately 50 mL of dry secretion was collected from a single quarter previously checked free of bacteria (plated overnight and evaluated visually for any CFU formation). On the last day of lactation (day 0) cows had samples collected and had all four quarters treated with a single 300 mg intramammary treatment of Cephapirin benzathine (ToMORROW, Boehringer Ingelheim, Missouri, USA). Single quarter dry cow secretion samples were collected aseptically, from the same quarter, on day 0, 3, 10, and 21 post dry-off.

### RNA isolation

Dry secretion samples were chilled on ice for 30 minutes, spun for 45 minutes at 4 °C at 10,000 × g, and had fat layers removed. From the remaining sample, 0.5 mL of dry secretion was collected from the supernatant of the sample, avoiding the cell pellet entirely. RNA was isolated from the dry secretion sample using the mirVana kit (Cat No. AM1560, Invitrogen, CA, USA) utilizing manufacturer’s instructions. Quality and quantity of RNA was determined using the Agilent 2100 small RNA chip bioanalyzer system (Cat No. G2938-90094, Agilent Technologies, CA, USA).

### Library preparation and sequencing

Libraries were prepared using the NEBNext Multiplex Small RNA Library Prep Set for Illumina Set 1 and 2 (New England BioLabs, Ipswich, MA, United States). Six microliters (6 µL) of each extracted dry secretion sample was individually indexed with one of the Illumina 1–23 indexed primers. Library PCR products were then purified to single- and double-stranded DNA fragments and concentrated to 35 µL using the QIAquick PCR purification kit (QIAGEN, Germantown, MD, United States). The quality and quantity of each library was determined using a 135–170 nucleotide gate on an Agilent 2100 Bioanalyzer High Sensitivity DNA chip (Agilent Technologies, Santa Clara, CA, United States). A total of 30 ng of each library was pooled into one of two pools. Libraries in each pool were created and size selected (142–170 nt) according to manufacturer’s instructions using the Pippin Prep on a 3% Agarose gel without Ethidium Bromide (SAGE Sciences, Beverly, MA, United States). After the gel was run, the pools were concentrated using the QIAquick PCR purification kit (QIAGEN, Germantown, MD, United States) by eluting in 32 µL of RNase-free water. The concentration of each pool was determined using a 135–170 nucleotide gate on an Agilent 2100 Bioanalyzer High Sensitivity DNA chip (Agilent Technologies, Santa Clara, CA, United States). The pool was stored at −20◦C until sequencing. Each pool was sequenced by 50 cycles on the Illumina 2500 System (Illumina, San Diego, CA, United States). Nucleotide sequence data reported was submitted and is available in the NCBI SRA database under the BioProject accession number PRJNA545722.

### Sequence analysis

The Illumina sequences were inspected for quality using FastQC v0.11.2 (http://www.bioinformatics.babraham.ac.uk/projects/fastqc), and the adapter was removed using cutadapt^21^. Sequences of bovine microRNAs and their precursors were downloaded from miRBase (www.mirbase.org, release^22^). Reads were mapped to known bovine microRNAs, and read counts for each microRNA were compiled using miRDeep2^22^. The bovine genome downloaded from Ensembl 96 was used as a reference. The novel miRNAs predicted by miRDeep2 were identified in at least five independent samples and over 200 mature reads.

### Statistical analysis

Normalized counts were calculated as (normalized counts) = (raw counts) * (mean library size)/(library size of individual sample)^23^. Two statistical models were fit to analyze the miRNA data.

#### Compound symmetry model

**Figure 1:**
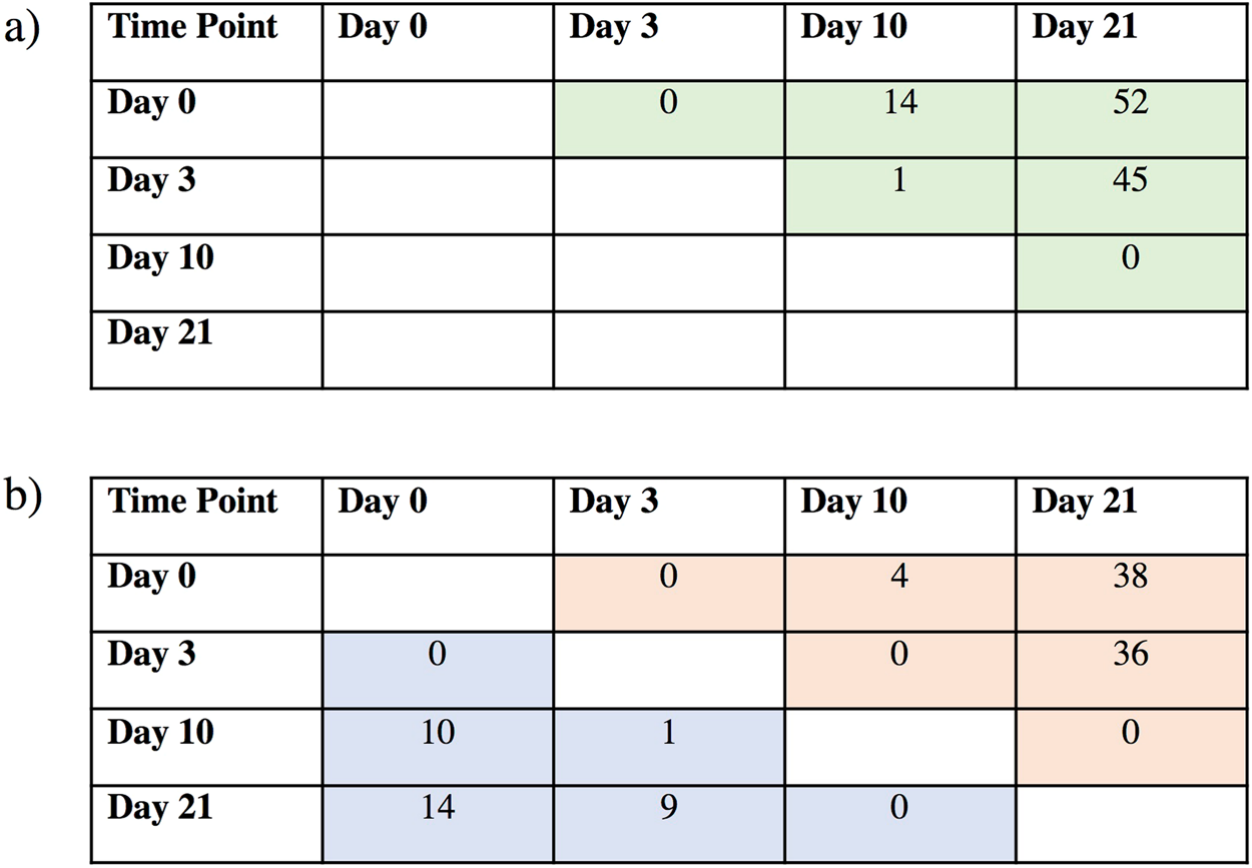
Matrices of the number of miRNAs whose expression significantly changed between experimental days of dry-off. (**a**) Depicts total number of miRNAs (green) differentiated between days 0, 3, 10, and 21 post dry-off. Of total significantly changed miRNAs, (**b**) depicts downregulated (blue) and upregulated (red) miRNAs.

A model was fit using PROC MIXED in SAS with the log_2_ normalized count as the response variable. A repeated measures model was fit to the data. In order to find the best fitting model across all miRNAs, different covariance structures were fit to the data including: compound symmetry (CS), heterogeneous compound symmetry, spatial power, Toeplitz, autoregressive (1), and heterogeneous autoregressive (1). Each miRNA was analyzed independently using the ‘by’ statement within PROC MIXED. The only fixed effect fit in the model was day. The best fitting model, CS, was chosen by AIC and AICC, which was less complex and contains two parameters to estimate within the covariance structure. More complex covariance structures did not converge well across all miRNA. The simple CS structure had the best convergence among all covariance structures attempted. This model will be referred to as the CS model. The overall F-statistic was extracted from each miRNA model along with the associated p-value. These p-values were then used as input for the qvalue package function from the qvalue package in Bioconductor^24^. For the time point contrasts seen in Fig. 1, each pairwise contrast estimate was extracted from the output using the ODS feature. The p-values for each contrast pair were then used as input for the q-value function (six separate pairwise contrasts). The miRNAs with q-values ≤ 5% were declared to have a statistically significant effect. The procedure yields a 5% FDR among significant miRNAs within each pairwise contrast.

#### Random regression model

A basic random regression (RR) model was fit using all miRNAs, cows, and time points into one parsimonious model. The model was fit using the lmer function within the lme4 package in R^25^. The following model was fit,

$$LN{C}_{ijk}=({\beta }_{0}+{\beta }_{{0}_{k}})+({\beta }_{1}+{\beta }_{{1}_{k}})Da{y}_{ij}+{e}_{ijk},$$

where *LNC*_*ijk*_ is the log_2_ normalized counts for the i^th^ cow at the j^th^ day for the k^th^ miRNA, *β*_0_ and *β*_1_ are the fixed intercept and slope for the regression of LNC on day post dry-off across miRNA, ${\beta }_{{0}_{k}}$ and ${\beta }_{{1}_{k}}$ are the random intercept and slope for each miRNA k as deviations from the respective means (*β*_0_ and *β*_1_), which have expectations equal to 0. *Day*_*ij*_ is the day post dry-off of the i^th^ cow at the j^th^ day, and *e*_*ijk*_ is a random residual. This model will be referred to as the RR model. The RR model fits the following covariance structure for the random intercept and slope terms,

$${var}(\begin{array}{c}{\beta }_{{0}_{k}}\\ {\beta }_{{1}_{k}}\end{array})=\,(\begin{array}{cc}{\sigma }_{{\beta }_{{0}_{k}}}^{2} & {\sigma }_{{\beta }_{{0}_{k}},{\beta }_{{1}_{k}}}\\ {\sigma }_{{\beta }_{{0}_{k}},{\beta }_{{1}_{k}}} & {\sigma }_{{\beta }_{{1}_{k}}}^{2}\end{array}),$$

where ${\sigma }_{{\beta }_{{0}_{k}}}^{2}$ is the variance of the random intercept terms among miRNAs, ${\sigma }_{{\beta }_{{1}_{k}}}^{2}\,$is the variance of the random slope terms among miRNAs, and ${\sigma }_{{\beta }_{{0}_{k}},{\beta }_{{1}_{k}}}$ is the covariance between the random intercept and slope terms.

#### Minimum regression slope threshold

The minimum slope threshold applied to collective regression slopes described above, was calculated as the positive and negative slope that would facilitate a two-fold miRNA expression change (of log2 (normalized counts +1)) over the 21 days of data collection.

## Results

In total, there were 146,497,558 cleaned reads processed, and 103,903,112 sequences that mapped to the reference genome. The mean library size per sample (six cows, each with four timepoints) was 6,104,065 ± 557,996 total cleaned reads, and 4,329,296 ± 591,016 sequences that mapped to the reference genome. Of total sequences that mapped to the bovine genome, 40,494,729 sequences were mapped to mature miRNA sequences. From those sequences, a total of 816 miRNAs were mapped back to previously known miRNAs (Supplemental Table 1) and additionally, there were 80 novel miRNAs identified (Supplemental Table 2). There were 6 novel miRNAs with sequences in at least two different genomic locations. There were 42 novel miRNAs derived from the negative strand and 38 from the positive strand. All novel miRNAs were mapped to a chromosome with the exception of bta-novel-miR103 and bta-novel-miR104. Novel microRNAs were mapped to most bovine chromosomes with the exception of chromosomes 3, 8, 22, 24, and 25.

Of total identified miRNAs (known and novel), 398 miRNAs were identified as having ten counts or more in least four of the six cows for at least one timepoint, and were evaluated for expression changes. The normalized counts for all 398 miRNAs evaluated can be seen in Supplemental Table 3. Of the 398 miRNAs, 30 were previously not in the bovine miRNA database and thus identified as novel miRNAs, and 368 were previously known (see Supplemental Table 3). Across all samples and all days, the 20 miRNAs with the highest normalized reads are shown in Table 1. Of the 20 miRNAs with the highest normalized counts, reads ranged from 2.18 × 10^5^ to 1.56 × 10^7^ (bta-let-7i and bta-miR148a, respectively).

**Table 1:** The twenty most highly expressed miRNAs by total normalized counts, over all animals, across experimental days, as well as shown by individual timepoint reads, across all animals.

miRNA	Normalized Total reads	Normalized Reads by Timepoint (across all cows)			
		Day 0	Day 3	Day 10	Day 21
bta-miR-148a	15597442.20	5023611.78	4616087.23	3385754.47	2571988.76
bta-miR-21-5p	4135607.81	322010.05	470583.70	1519926.87	1823087.20
bta-let-7a-5p	2803335.02	729089.13	721726.22	696472.88	656046.79
bta-miR-26a	1748092.80	446942.40	514996.93	347867.09	438286.29
bta-let-7b	1683550.58	283215.70	401282.76	451190.55	547861.57
bta-miR-200b	1373598.77	339175.95	342872.49	319014.98	372535.35
bta-miR-99a-5p	1343424.18	346288.40	369600.24	240152.43	387383.11
bta-miR-200c	1267996.91	389330.79	356600.95	247529.57	274535.60
bta-miR-30a-5p	1250619.00	271701.59	297919.85	313162.09	367835.47
bta-mir-1246	876050.95	22071.75	72759.28	519853.84	261366.08
bta-let-7f	828603.66	135397.60	176359.30	270699.31	246147.45
bta-miR-200a	592502.74	156648.69	188751.70	121119.42	125982.93
bta-let-7g	568498.98	94948.84	129127.76	158613.97	185808.41
bta-miR-30d	528901.19	164602.98	159179.32	97818.87	107300.02
bta-let-7c	510609.08	176686.64	139030.77	97365.35	97526.32
bta-miR-22-5p	399233.63	43915.330	45393.50	159316.19	150608.66
bta-miR-151-5p	306251.68	103386.88	90243.91	61812.86	50808.03
bta-miR-27b	304527.30	63626.40	59217.08	74380.00	107303.82
bta-miR-320a	238753.39	82066.64	56386.81	48153.46	52146.48
bta-let-7i	218016.09	38036.73	41142.92	49295.22	89541.22

Of primary interest are the miRNAs with significant changes in reads over time from the cessation of lactation (day 0) through day 21 of the dry period in dry secretions. Using a compound symmetry model (CS), a total of 46 miRNAs were significantly upregulated or down regulated (q-value < 0.05) over the four time points, including seven novel miRNAs (Table 2). Additional studies will need to be pursued to verify the novel miRNAs and ascertain their function. The most significantly down regulated miRNA was miR-22-3p (q-value = 0.006), and the most significantly upregulated was miR-106a (q-value = 0.006). The breakdown of total miRNAs, and the number of upregulated and downregulated miRNAs with significant  differences in count numbers by experimental day are shown in (Fig. 1).

**Table 2:** The miRNAs with significant changes (q-value < 0.05) over experimental days and their corresponding slope identified by random regression of normalized log transformed counts.

miRNA		q-value	Direction	Slope ± SE	Verified	Biological Association	Reference
**1**	bta-miR-106a	0.0060	Up	0.0815 ± 0.0253	Yes	Lactation, Pregnancy	^8,30^
**2**	bta-miR-22-3p	0.0060	Down	−0.2947 ± 0.0253	Yes	Lactation	^8^
**3**	bta-miR-365-5p	0.0060	Down	−0.1518 ± 0.0253	Yes	Infection	^35,38^
**4**	bta-miR-31	0.0091	Up	0.1214 ± 0.0253	Yes	Infection, Lactation	^26,35,38^
**5**	bta-miR-1307	0.0109	Down	−0.0603 ± 0.0253	Yes	Infection	^35,38^
**6**	bta-miR-10174-3p	0.0113	Down	−0.1086 ± 0.0253	Yes	NA	
**7**	bta-miR-11978	0.0113	Down	−0.0896 ± 0.0253	Yes	NA	
**8**	bta-miR-15a	0.0113	Up	0.0669 ± 0.0253	Yes	Pregnancy	^31^
**9**	bta-mir-874	0.0141	Up	0.0814 ± 0.0253	Yes	Infection, Lactation	^8,35,38^
**10**	bta-mir-378d	0.0163	Up	0.0886 ± 0.0253	Yes	Infection	^35,38^
**11**	bta-miR-130b	0.0169	Up	0.1278 ± 0.0253	Yes	Infection	^35,38^
**12**	bta-miR-365-3p	0.0169	Down	−0.1406 ± 0.0253	Yes	NA	
**13**	bta-miR-504	0.0169	Up	0.0770 ± 0.0253	Yes	NA	
**14**	bta-miR-615	0.0169	Down	−0.0603 ± 0.0253	Yes	Infection	^9^
**15**	bta-miR-218	0.0302	Up	0.0986 ± 0.0253	Yes	Infection, Lactation	^8^
**16**	bta-miR-11975	0.0371	Down	−0.0725 ± 0.0253	Yes	NA	
**17**	bta-mir-11986c	0.0371	Up	0.0538 ± 0.0253	Yes	NA	
**18**	bta-miR-130a	0.0371	Up	0.0802 ± 0.0253	Yes	NA	
**19**	bta-mir-1343	0.0371	Down	−0.0522 ± 0.0253	Yes	NA	
**20**	bta-miR-194	0.0371	Up	0.0167 ± 0.0253	No	Pregnancy	^39^
**21**	bta-miR-21-3p	0.0371	Up	0.0798 ± 0.0253	Yes	Infection, Lactation	^26,35,38^
**22**	**bta-novel-miR62**	**0.0371**	**Down**	−0.0212 ± 0.0253	**No**	—	
**23**	**bta-novel-miR64**	**0.0371**	**Up**	0.1122 ± 0.0253	**Yes**	—	
**24**	bta-mir-30c	0.0377	Down	−0.0144 ± 0.0253	No	Infection	^35^
**25**	bta-miR-34a	0.0377	Up	0.0178 ± 0.0253	No	Infection	^9^
**26**	bta-miR-193b	0.0421	Up	0.0670 ± 0.0253	Yes	Infection, Lactation, Pregnancy	^8,39^
**27**	bta-novel-miR42	0.0421	Up	0.1113 ± 0.0253	Yes	—	
**28**	**bta-novel-miR45**	**0.0421**	**Up**	0.0912 ± 0.0253	**Yes**	—	
**29**	**bta-miR-183**	**0.0421**	**Up**	0.0279 ± 0.0253	**No**	Infection, Pregnancy	^10,31^
**30**	bta-miR-195	0.0421	Up	0.0491 ± 0.0253	Yes	NA	
**31**	**bta-novel-miR33**	**0.0421**	**Up**	0.0950 ± 0.0253	**Yes**	—	
**32**	bta-miR-452	0.0431	Up	0.0791 ± 0.0253	Yes	Infection, Lactation	^35^
**33**	bta-miR-128	0.0432	Down	−0.0596 ± 0.0253	Yes	Lactation	^38^
**34**	bta-miR-1468	0.0432	Down	−0.0380 ± 0.0253	No	Infection	^35,38^
**35**	bta-miR-455-5p	0.0432	Up	0.0748 ± 0.0253	Yes	Infection	^38^
**36**	bta-miR-542-5p	0.0432	Up	0.0808 ± 0.0253	Yes	Infection, Lactation	^8,38^
**37**	**bta-novel-miR39**	**0.0432**	**Up**	0.1440 ± 0.0253	**Yes**	—	
**38**	bta-miR-2443	0.0433	Down	−0.0676 ± 0.0253	Yes	Lactation	^8^
**39**	**bta-novel-miR24-1**	**0.0433**	**Up**	0.1624 ± 0.0253	**Yes**	—	
**40**	bta-miR-450a	0.0436	Up	0.0549 ± 0.0253	Yes	Infection	^38^
**41**	bta-miR-1388-3p	0.0485	Up	0.0333 ± 0.0253	No	Infection	^35^
**42**	bta-miR-140	0.0485	Up	0.0401 ± 0.0253	No	Pregnancy	^31^
**43**	bta-miR-24-5p	0.0485	Down	−0.0049 ± 0.0253	No	NA	
**44**	bta-miR-29a	0.0485	Up	0.0411 ± 0.0253	No	Lactation	^8^
**45**	bta-mir-7180	0.0485	Down	−0.0408 ± 0.0253	No	NA	
**46**	bta-miR-9-3p	0.0485	Up	0.0308 ± 0.0253	No	Infection	^35,38^

SE values represent standard error. Verified status refers to whether the differentially expressed miRNA was in the top or bottom 5% of all random regression slopes respectively. Biological association refers to whether the miRNA was found to be associated in existing literature related to lactation, pregnancy, or infection respectively. Novel miRNAs are bolded.

**Figure 2:**
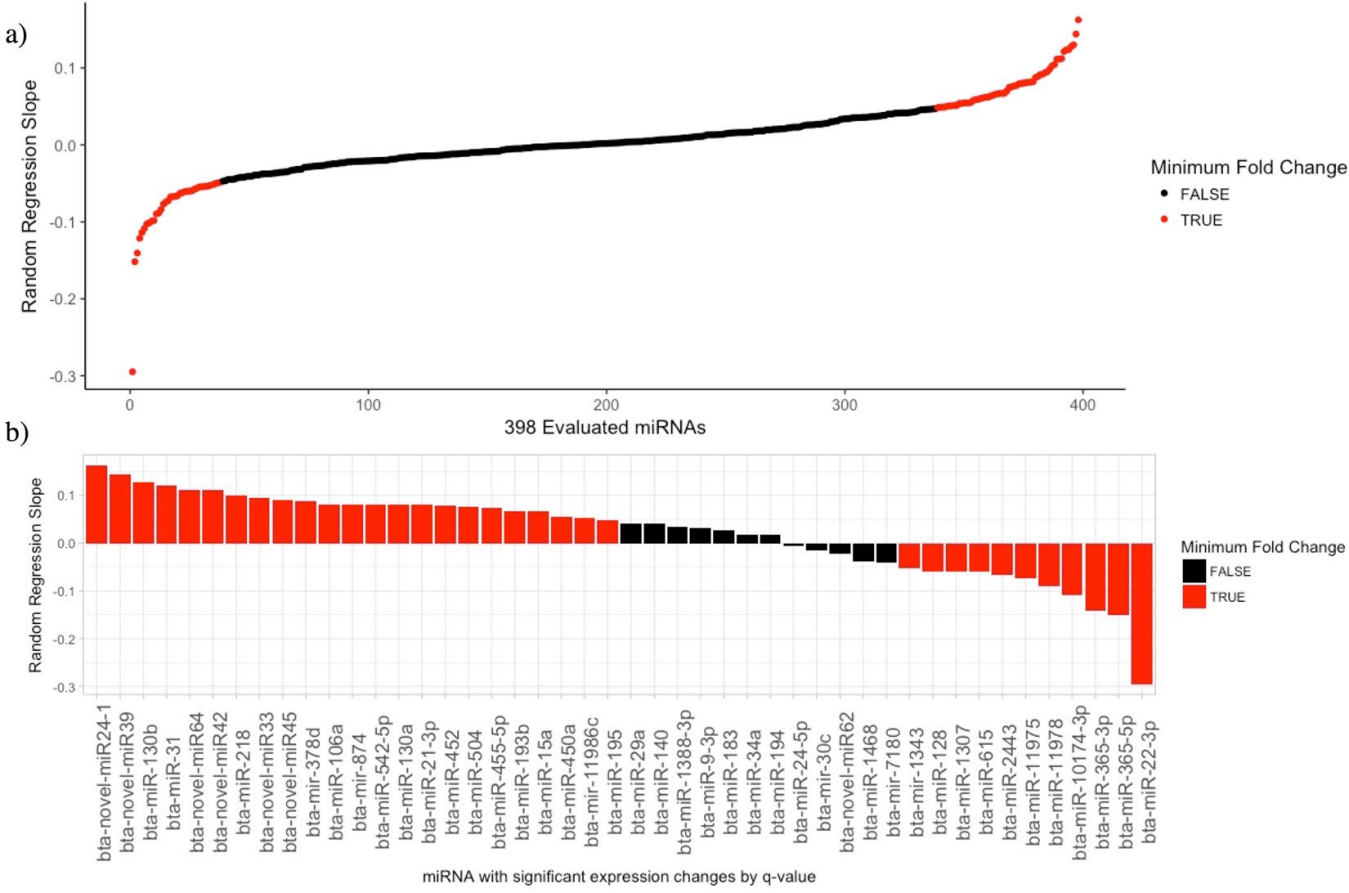
Random regression slope evaluation of miRNA with expression changes. Shown are the miRNA with a minimum two fold expression change over the first 21 days of the dry period using a random regression slope of log2 (normal counts +1) in all evaluated 398 miRNA (**a**), and the 46 significant miRNA (q-value < 0.05) identified by the CS model (**b**) (also, see Table 2).

**Figure 3:**
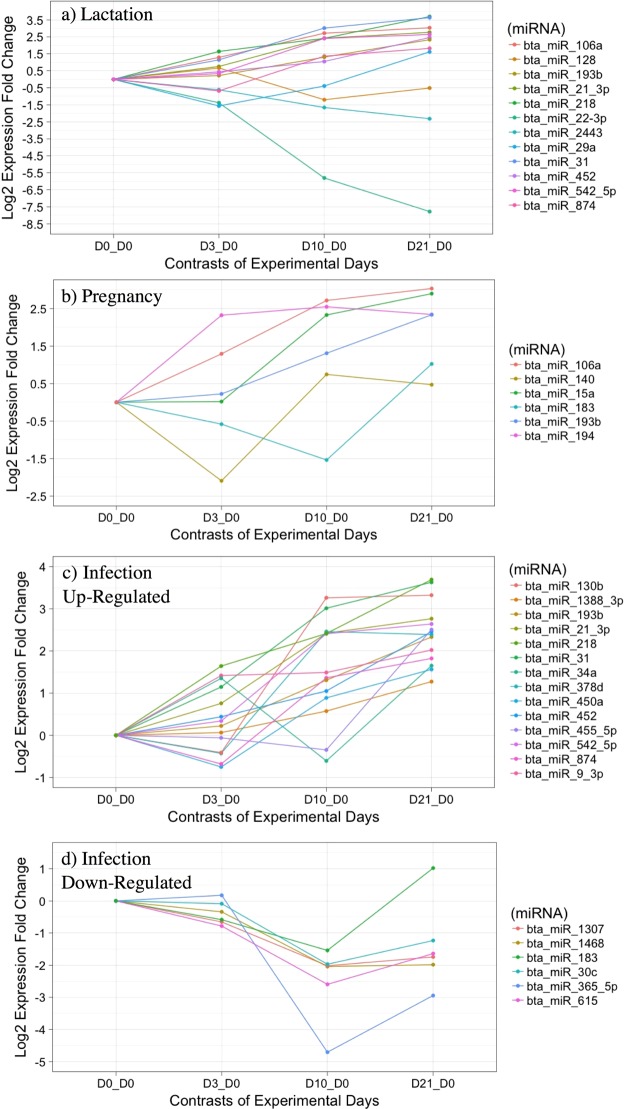
The fold change differences of log2 (normal counts +1) between experimental days and last day of lactation. Panels characterize fold change differences of miRNAs with significant (q-value < 0.05) changes over experimental days associated with existing literature related to lactation (**a**), pregnancy (**b**), and infection (**c**,**d**) which was separated into up-regulated miRNAs associated with infection (**c**) and downregulated miRNAs associated with infection (**d**) for clarity. See Table 2 for reference details.

Of the 46 significant miRNAs that changed across time (q-value < 0.05), 16 were down regulated and 30 were upregulated. In an additional analysis, we evaluated the slopes of log transformed normalized counts using a random regression model over time for each individual miRNA (Fig. 2). The most negative slope belonged to miR-22-3p, which was also identified to be the most significantly downregulated with the CS model (q-value = 0.006). The same was true for the miRNA with the most positive slope, bta-novel-miR24-1, also found to be significantly upregulated (q-value = 0.0433). The random regression slopes for the 398 miRNAs are plotted in Fig. 2a. The slope thresholds for the highlighted miRNAs were based on a minimum of a twofold change in expression over the 21 days of dry period. Figure 2b depicts the 46 significant miRNAs identified by the CS model, and whether they appeared in the top and bottom of random regression slopes. We found that 11 out 16 of the downregulated (68.75%), and 23 out of 30 upregulated miRNAs (76.67%) were identified by both methods, suggestive that the analysis of random regression slopes was useful to cross verify miRNAs with count or read differences. Further, miRNAs with the most significant q-values (q-value < 0.01) were 100% identified by regression slope method for both up and downregulated miRNAs. The 46 miRNAs with significant changes in expression, their slopes, and whether they cross verified is summarized in Table 2. We manually curated these 46 miRNAs with previous published data sets on bovine miRNAs and gave them a biological association based on this literature. We reported both upregulated and downregulated miRNAs found to be identified with the most biological relevance to the current study being associated with lactation, pregnancy, or infection (Fig. 3).

## Discussion

The dry period in a dairy cow’s lactation cycle results in significant changes to the mammary gland. We obtained mammary secretions on various day after the cession of milking to determine the changes in miRNAs as the cows progressed through the physiological process of dry-off. Nearly 12% (46/398) of the miRNAs that had sufficient data for quantification demonstrated expression changes. To further analyze our results, we developed a linear slope regression analysis that supported the traditional modeling approach to identify miRNAs from dry secretions whose expression changed significantly over time. Of the miRNAs that had significant count changes with experimental days (q-value < 0.05), 68.75% and 76.67% cross verified in both methods for downregulated and upregulated miRNAs respectively (Fig. 2b). Additionally, miRNAs with significant expression changes, were cross referenced with known miRNAs in the literature and found to be associated with lactation, pregnancy and reproduction, as well as inflammation and disease.

In dairy cattle, emphasis is placed on expression profiles associated with lactation. In a large study following miRNA expression patterns through a complete lactation cycle, miR-874 was found to be the highest differentially expressed miRNA between the galactopoiesis and involution stages of lactation, and miR-29a was differentially expressed between involution and lactogenesis^8^. MiR-106a, was significantly upregulated in this study. However, MiR-106a was found to be downregulated in the galactopoiesis, involution, and lactogenesis lactation phases^8^. In the same study miR-22-3p is upregulated between galactopoiesis and lactogenesis^8^, but is downregulated in our findings here in the dry period. MiR-21-3p is upregulated in this study, and is similar to the upregulation of miR-21 in early lactation, with suggested roles in the mechanism of proliferation of cells within the mammary gland^26^, which we know is a cellular process important during the dry-off period. While not related to lactation stage but milk composition, miR-130a expression, downregulated in this study, is inversely proportionate to lipid droplet formation and could be linked to alterations in mammary gland secretion components^27^. We are additionally interested in the relationship of changing miRNAs over the dry period and how they may correlate with changing dry secretion composition. This is a subject our lab group is currently investigating with proteomic profiles of these dry secretion samples.

All cows utilized in the study were confirmed pregnant, thus miRNAs associated with reproduction and embryo development would be expected. From the literature, miR-130b and miR-106a, both up regulated in our study, have been shown to functionally regulate genes or to play a role in bovine reproductive, oocyte, and embryo development^28,29^. MiR-106a has also been shown to respond to interferon tau *in vitro* in a bovine endometrial cell culture, which has important implications on the crosstalk between fetal embryos and maternal cells, especially important for embryo implantation^30^. MicroRNAs miR-15a (upregulated in this study) and miR-128 (downregulated in this study) were both characterized in milk cell and skim cell fractions, and although they are not yet recommended as biomarkers of bovine pregnancy detection, miRNA analysis did yield distinct differences between pregnant and cyclic cows^31^.

Many of the significantly expressed miRNAs identified have previously been associated with inflammatory responses in blood, mammary tissue, and milk. The dry-off period represents vast physiological and immunological changes within the dairy cow, and identifying behaviors of miRNAs could be valuable in contributing to the understanding of dry-off health. Consistent with our results, members of the bta-let-7 family (let-7a, b, c, f, g, i in this study) are highly expressed, highly conserved, and found to be associated with dicer/miRNA regulation^32,33^. While miR-34a was downregulated and miR-615 was up regulated in monocytes responding to a *Streptococcus uberis* mastitis disease challenge^9^, during dry-off we see the inverse where miR-34 is upregulated and miR-615 is downregulated. Other miRNAs, such as miR-140 and miR-31, have also been associated with up regulation in liver samples under severe negative energy balance in periparturient cattle^33^ as well as upregulated in the data shown herein. Upregulated in the present study, miR-21 was found upregulated in mastitis milk compared to healthy control milk^34^. In a look at microRNA significantly differentially expressed in the mammary gland of *staphylococcus aureus* infected animals compared to controls miR-378 was found to be upregulated^35^ as we show miR-378d was upregulated during dry-off. Interestingly, miR-218 and miR-106a were upregulated in the current study and similarly found to be up regulated in post-partum metritis cows compared to healthy counterparts^36^. In the same study, while miR-31 was upregulated in our data set, it was found to be downregulated in the metritis cows compared to healthy controls^36^.

Collectively, many of the miRNAs that were differentially expressed over the first three weeks of dry-off have been found in the literature to be associated with components of lactation, pregnancy, reproduction, and inflammation. The relevance of miRNAs expression changes associated with stages of lactation and pregnancy were biologically supported by our experimental design, and established that dry secretions are a viable sample for miRNA detection. Since dairy cattle are pregnant during the dry-off period, associating biological functions to changes in miRNA expression is confounded by the multiple physiological changes due to the cessation of lactation and late stage pregnancy. Furthermore, dry secretions are known to have inhibitory microbial properties for gram- negative bacteria^37^. Therefore, the study of the changes from milk to dry secretion could yield information about the antimicrobial properties of dry secretions. The miRNAs associated with disease and regulation of inflammation could shed light on the tremendous immunological and physiology changes undergone by the dairy cow and the mammary gland during the dry-off period and how these are and could be utilized to protect the mammary gland against infection.

The number of miRNAs that change significantly over the first 21 days of the dry period suggest that the dry-off period is one of significant biological modulation. Dry secretion sampling over various time points of the dry-off process may help capture information about reproductive health, lactation condition, and relevant inflammatory state of cows as their bodies prepare for the periparturient period and lactation.

## Supplementary information

Supplementary information 

## Data Availability

The datasets generated during and/or analyzed during the current study are available from the corresponding author on reasonable request, or included in the published article and its supplemental material.
