# Supplementary material for: MicroRNA profiles of dry secretions through the first three weeks of the dry period from Holstein cows
Source: Sci Rep. 2019 Dec 23;9:19658. doi: 10.1038/s41598-019-56193-5 (PMC6928067; doi:10.1038/s41598-019-56193-5)
Supplement: Supplementary file 1 — Supplementary information [file 41598_2019_56193_MOESM1_ESM.pdf]

Supplementary Information

MicroRNA profiles of dry secretions through the first three weeks of the dry period from Holstein cows

Ellie J Putz[1,2], Austin M Putz[3], Hyeongseon Jeon[4], John D Lippolis[1], Hao Ma[1], Timothy A. Reinhardt[1], and Eduardo Casas[1*]

[1]Ruminant Diseases and Immunology Research Unit
USDA Agriculture Research Service
National Animal Disease Center, Ames, IA
[2]Oak Ridge Institute for Science and Education
Oak Ridge Associated Universities, Oak Ridge, TN
[3]Animal Breeding and Genetics,
Iowa State University, Ames, IA
[4]Department of Statistics,
Iowa State University, Ames, IA

*__Corresponding author__:
Eduardo Casas
eduardo.casas@usda.gov

Supplemental Table 1. All miRNA that mapped to known bovine miRNAs

**miRNA**	**Sequence**
bta-miR-10162-5p	uggacaggccaagccgcugug
bta-miR-10164-3p	aacuguuaggaggcuuggcu
bta-miR-10165-5p	gagccugcaggugacgagga
bta-mir-10183	cucgcugugggguccgaggcggg
bta-mir-10184	uuuaauguagaaaacccaaugg
bta-let-7a-3p	cuauacaaucuacugucuuucc
bta-let-7a-5p	ugagguaguagguuguauaguu
bta-let-7b	ugagguaguagguugugugguu
bta-let-7c	ugagguaguagguuguau
bta-let-7d	agagguaguagguugcauaguu
bta-let-7e	ugagguaggagguuguauaguu
bta-let-7f	ugagguaguagauuguauaguu
bta-let-7g	ugagguaguaguuuguacaguu
bta-let-7i	ugagguaguaguuugugcuguu
bta-miR-1	uggaauguaaagaaguauguau
bta-miR-100	aacccguagauccgaacuugu
bta-miR-101	uacaguacugugauaacuga
bta-miR-10166-3p	caggcugugugcagcugaccaca
bta-miR-10166-5p	ugugcagcugaucacagacugug
bta-miR-10167-3p	gcggguggucggggcggguc
bta-miR-10167-5p	gacgggcgucuccuccgcggg
bta-miR-10170-3p	cggguuaccgucacgucggag
bta-miR-10170-5p	accggcgcagcggcugaccuggg
bta-miR-10172-5p	ucacugggcauccucugcuuu
bta-miR-10173-5p	uugcucccuccacaccuccaga
bta-miR-10174-3p	uggguuccuggcaugcugauu
bta-miR-10174-5p	aucacauugccagggauuaccacg
bta-miR-10175-5p	uggagagaacagguggcuuu
bta-miR-10176-5p	aggaggagagagcugcaagu
bta-miR-10179-3p	ccccgccugcccaccuguccagg
bta-miR-10179-5p	ucugcagggugguaggcugugg
bta-miR-10180-3p	gaggaggggcgcgcugugcucu
bta-miR-10181-5p	ugugggaaggagcugugcug
bta-miR-10182-3p	caguccggucccgcggugucuccgc
bta-miR-10182-5p	caguccggucccgcggugucuccg
bta-miR-10185-5p	gagccgcggagggagcgag
bta-miR-10225a	ccgagccugacagaucacaca
bta-mir-10225b	ucgagccugacagaucacaca
bta-miR-103	agcagcauuguacagggcuauga
bta-miR-106a	aaaagugcuuacagugcagguag
bta-miR-106b	ccgcacuguggguacuugcu

bta-miR-107	agcagcauuguacagggcuau
bta-miR-10a	uacccuguagauccgaauuugu
bta-miR-10b	uacccuguagaaccgaauuugu
bta-miR-1185	auauacagagggagacucuu
bta-miR-11971	ugaggggcagagagugagaa
bta-mir-11972	ggggcgggagcggccggggguc
bta-miR-11973	cuugcugagugaccucccugcu
bta-mir-11974	agcaccugcugugucccccacagc
bta-miR-11975	gcggcggcggcgccggggcgcgc
bta-mir-11976-2	ggcggcggcggcgccggggcg
bta-miR-11977	ugucucaguucagcaggaag
bta-miR-11978	acucuucccucccucggccggc
bta-miR-11979	ucagugaucagugccugguggg
bta-mir-11980	aggcaacgggcuuggcggag
bta-miR-11981	cagggcgggaacgggcugcggg
bta-mir-11982	uucggcgccaccacccugcgggu
bta-miR-11983	gaccagugcgaggccuguggga
bta-miR-11984	acacgcguccuuggauccugac
bta-mir-11985	cccaccgcucuccucccgcc
bta-mir-11986	uuguccaaugaguuaguccuu
bta-miR-11986b	uuuuccaaugaguuaguccuuu
bta-mir-11986c	gaacugaguccuuuggaaaaga
bta-mir-11987	cgaggaaucucugguggaggu
bta-mir-11988	aaggggacgacagaggaugaga
bta-miR-11989	cccagggauguagcuccu
bta-mir-11991	auguucuguaucuugauugua
bta-miR-11992	ccuaggggauugacauagucu
bta-miR-11993	gaccugugcguguacacucaug
bta-miR-11994	uccaaaacgggaccuuucug
bta-miR-11995	uuguccuacuucucagcugucu
bta-miR-11996	aucugagagcacuuugucaagg
bta-mir-11997	aaccaagggugugggccgaga
bta-miR-11998	uucucuaguguguuuucccaac
bta-miR-11999	cagacccauauucucauaggc
bta-miR-12000	uugaauccacagaugugagacc
bta-miR-12001	ccugugagggcuucacccuggac
bta-miR-12002a	cauguggaauuuuuaguugcu
bta-miR-12002b	ccucauguggaaucuuuagcug
bta-miR-12003	auuggguuggccauagguucu
bta-mir-12004	cuggcucuggcucucugccuccu
bta-miR-12005	cuccccuuaaggacuucau
bta-miR-12006	gguugaaaagaguuucgguagu
bta-mir-12007	uggcucuuuuacuggcgucucu

bta-miR-12008	cuuuaacucuucauccuuuug
bta-miR-12009	aguggagggaacuugcuugu
bta-miR-12010	auggaggcugggagugcaugagcu
bta-miR-12012	gugcucuagagcucacaggcc
bta-miR-12013	cccgaguuugggcagcaguuc
bta-miR-12014	ugccacgggcugggccuuggga
bta-miR-12015	caggucccugccgggcggaga
bta-miR-12016	uacugauuugacuuacauacauu
bta-miR-12017	acagaggagcuuuugacaugaacu
bta-miR-12018	aucuuguccgagaaugucuugaa
bta-miR-12021	uugagaaaaaucugccagc
bta-miR-12023	uccucccgcccccccgcg
bta-miR-12024	uuaggauccaaacaacacuga
bta-miR-12025	ucgcgggguuuugcagaggacu
bta-miR-12027	ugccgcgcuguuucccuggugugc
bta-mir-12028	ugucugggaccccgauucucu
bta-mir-12030	cccggggcccggagcggcc
bta-miR-12031	uaauuggagccagucugcag
bta-mir-12034	ccccggggagcccggcggu
bta-miR-12035	acaccaggacuugucuccccaga
bta-miR-12036	cggcuccgugacucguccgugg
bta-mir-12037	cauucugucuucucuuuagu
bta-miR-12038	aacauugacagcuucuuugcaga
bta-miR-12039	ccagggcccuggcuucuucu
bta-miR-12042	ugugucuuuccuucugugugugcug
bta-miR-12043	ccuccagggcuaggagguggguc
bta-mir-12044	aauggauuacuucugauugcu
bta-miR-12045	acccgggaagucccugacacu
bta-miR-12046	cuucugcucccugcccuguag
bta-mir-12047	caggaaacaggaccuuugccu
bta-miR-12048	caaggaggaggugaagcaccaga
bta-miR-12050	uggcugcugucguccuuccuac
bta-miR-12052	cccaaugagauuucaaacugaga
bta-miR-12053	uauccaggacuaggucugcag
bta-mir-12056	cugggaugggguugggaaggg
bta-miR-12057	acugggaguggaaggagag
bta-mir-12058	guucuuccaguagucaugugc
bta-mir-12059	ucuccucccacccugccuccu
bta-mir-12060	uugggagaccaggggaagacu
bta-miR-12061	ugugggaccugggucuugugg
bta-miR-12062	ugggaugguccugugucacu
bta-miR-12064	ugguaguauuccuaguguggc
bta-miR-122	uggagugugacaaugguguuug

bta-mir-1224	gugaggacucgggagguggag
bta-mir-1225	ccgagccccugugccgcccccag
bta-mir-1246	aauggauuuuuggagcagg
bta-mir-1248-2	accuucuuguauaagcacugugcuaaa
bta-miR-1249	acgcccuuccccccuucuuc
bta-miR-1251	acucuagcugccaaaggcgcu
bta-miR-125a	ucccugagacccuuuaaccugu
bta-miR-125b	ucccugagacccuaacuugug
bta-mir-1260b	aucccaccacugccacca
bta-miR-126-3p	cauuauuacuuuugguacgcg
bta-miR-126-5p	ucguaccgugaguaauaaugcg
bta-miR-127	ucggauccgucugagcuuggcu
bta-miR-1271	cuuggcaccuaguaaguacuca
bta-miR-1277	uacguagauauauauguauuu
bta-miR-128	ucacagugaaccggucucuuu
bta-miR-1281	gaggguucggaggguggg
bta-mir-1287	ugcuggaucagugguuugaguc
bta-miR-129	cuuuuugcggucugggcuugc
bta-mir-1291	uggcccugacugaagaccugcagu
bta-miR-129-3p	aagcccuuaccccaaaaag
bta-miR-129-5p	cuuuuugcggucugggcuugc
bta-mir-1296	uuagggcccuggcuccaucucc
bta-miR-1298	uucauucggcuguccagaug
bta-miR-1301	uugcagcugccuaggagugauu
bta-mir-1306	ccaccuccccugcaaacgucc
bta-miR-1307	acucggcguggcgucggucgugg
bta-miR-130a	cagugcaauguuaaaaggg
bta-miR-130b	cagugcaaugaugaaagggc
bta-miR-132	uaacagucuacagccauggucg
bta-miR-133a	uugguccccuucaaccagcugu
bta-miR-133b	uuugguccccuucaaccagcu
bta-miR-133c	cagcugguugaaggggac
bta-miR-134	ugugacugguugaccagagugg
bta-mir-1343	cuccuggggcccgcacucuc
bta-miR-135a	uauggcuuuuuauuccuaugug
bta-miR-135b	uauggcuuuucauuccuaugug
bta-miR-136	caucaucgucucaaaugagucu
bta-miR-138	agcugguguugugaauca
bta-miR-1388-3p	aucucagguuugucagcccg
bta-miR-1388-5p	aggacuguccaaccugagaau
bta-miR-139	ucuacagugcacgugucuccagu
bta-miR-140	uaccacaggguagaaccacgga
bta-miR-141	uaacacugucugguaaagaug

bta-miR-142-3p	aguguuuccuacuuuaugga
bta-miR-142-5p	cccauaaaguagaaagcacu
bta-miR-143	ugagaugaagcacuguagcuc
bta-miR-1434-3p	aagaaaucuaaggucugagg
bta-miR-1434-5p	aaauuucuucacuuugac
bta-miR-144	uacaguauagaugauguacu
bta-miR-145	guccaguuuucccaggaaucccu
bta-miR-1468	cuccguuugccuguuuugcuga
bta-miR-146a	ugagaacugaauuccauaggu
bta-miR-146b	ugagaacugaauuccauaggcugu
bta-miR-147	gugugcggaaaugcuucug
bta-miR-148a	ucagugcacuacagaacuuugu
bta-miR-148b	ucagugcaucacagaacuuugu
bta-miR-148d	ucggagugucucagaacuuu
bta-miR-149-3p	gagggagggacgggggcugugc
bta-miR-149-5p	ucuggcuccgugucuucacu
bta-miR-150	ucucccaacccuuguaccagug
bta-miR-151-3p	ucgaggagcucacagucuagu
bta-miR-151-5p	cuagacugaagcuccuugagg
bta-miR-152	ucagugcaugacagaacuugg
bta-miR-153	gucauuuuugugaucugcagc
bta-miR-154a	aucauacacgguucaccuauuu
bta-miR-154b	agaggucuuccauggugcauucg
bta-miR-154c	agauauugcacgguugaucucu
bta-miR-155	uuaaugcuaaucgugauaggggu
bta-mir-1584	uuggggcugggcugggggca
bta-miR-1584-5p	ucucuccgcagaccucagc
bta-miR-15a	uagcagcacauaaugguuugu
bta-miR-15b	uagcagcacaucaugguuu
bta-miR-16a	uagcagcacguaaauauuggu
bta-miR-16b	uagcagcacguaaauauuggc
bta-miR-17-3p	acugcagugaaggcacuugua
bta-miR-17-5p	caaagugcuuacagugcagguag
bta-mir-1777a	ugggggcggugggggcggg
bta-mir-1777b	gggggcgguggggggcgggg
bta-mir-1814c	guuuuguuuggguuuguuuu
bta-miR-181a	aacauucaacgcugucggugagu
bta-miR-181b	aacauucauugcugucgguggguu
bta-miR-181c	aacauucaaccugucggugagu
bta-miR-181d	aacauucauuguugucgguggguu
bta-miR-182	uuuggcaaugguagaacucacacu
bta-miR-183	uauggcacugguagaauucacu
bta-miR-1839	aagguagauagaacaggucuug

bta-miR-184	uggacggagaacugauaagggu
bta-miR-1842	uuggcucugugaggucggcu
bta-mir-1843	aagcuucguggucucugucuugcc
bta-miR-185	uggagagaaaggcaguuccuga
bta-miR-186	caaagaauucuccuuuugggcu
bta-mir-187	ucgugucuuguguugcagccgg
bta-mir-188	caucccuugcauggugagggu
bta-miR-18a	acugcccuaagugcuccuucu
bta-miR-18b	uacugcccuaaaugcuccuucu
bta-miR-190a	ugauauguuugauauauuagguug
bta-miR-190b	ugauauguuugauauuggguug
bta-miR-191	caacggaaucccaaaagcagcu
bta-miR-1911	caccaggcauugguggucucugc
bta-mir-191b	gaacgaaauccaagcgcagcug
bta-miR-192	cugaccuaugaauugacagcc
bta-miR-193a-3p	aacuggccuacaaaguccc
bta-miR-193a-5p	ugggucuuugcgggcgagaug
bta-miR-193b	aacuggcccacaaagucccgcu
bta-miR-194	uguaacagcaacuccaugugg
bta-mir-1949	uauaccaggaugccagcauaguu
bta-miR-194b	cacauggaguugcuguuacaa
bta-miR-195	uagcagcacagaaauauuggca
bta-miR-196a	uagguaguuucauguuguuggg
bta-miR-196b	uagguaguuuccuguuguuggg
bta-miR-197	uucaccaccuucuccacccagc
bta-miR-199a-3p	caguagucugcacauugguu
bta-miR-199a-5p	uacaguagucugcacauugguu
bta-miR-199b	uacaguagucugcacauugguu
bta-miR-19a	ugugcaaaucuaugcaaaacuga
bta-miR-19b	ugugcaaauccaugcaaaacug
bta-miR-200a	uaacacugucugguaacgaug
bta-miR-200b	uaauacugccugguaaugauga
bta-miR-200c	uaauacugccggguaaugaugga
bta-miR-204	gcugggaaggcaaagggacgu
bta-miR-205	uccuucauuccaccggagucugu
bta-miR-206	uggaauguaaggaagugugugg
bta-miR-208b	uauaagacgaacaaaaggu
bta-miR-20a	uaaagugcuuauagugcagguag
bta-miR-20b	caaagugcucacagugcagguag
bta-miR-210	cugugcgugugacagcggcuga
bta-miR-211	uucccuuugucauccuuugccc
bta-miR-212	accuuggcucuagacugcuuac
bta-miR-21-3p	caacagcagucgaugggc

bta-miR-214	ugccugucuacacuugcugu
bta-miR-215	augaccuaugaauugacagaca
bta-miR-21-5p	uagcuuaucagacugauguugac
bta-mir-217	uacugcaucaggaacugauuggau
bta-miR-218	uugugcuugaucuaaccaugugg
bta-miR-219	uguccaaacgcaauucucgagu
bta-mir-219-2	agaguugagucuggacgucccg
bta-miR-219-3p	agaauuguggcuggacaucugug
bta-miR-219b-3p	gauguccagccacaauucucg
bta-miR-221	agcuacauugucugcuggguuu
bta-miR-222	agcuacaucuggcuacugggucucu
bta-mir-223	ugucaguuugucaaauacccca
bta-miR-22-3p	aguucuucaguggcaagcuuu
bta-miR-224	caagucacuagugguuccguuu
bta-miR-22-5p	aagcugccaguugaagaacugu
bta-miR-2283p	aauuguucauuugguuuuu
bta-mir-2284a	aaaaguucguuggguuuuu
bta-miR-2284aa	aaaaaguuuguuuggguuuucu
bta-miR-2284ab	uaaaaguuugguuggguuuuu
bta-mir-2284b	aaaaguucguuugguuuuuuc
bta-miR-2284c	cggaaaaacucuaaugaacuuu
bta-mir-2284d	aaaaaguucguuaggguuuuuc
bta-miR-2284f	aaaaguucguucggauuuuucc
bta-mir-2284g	aaaaguucguucggcuuuuu
bta-mir-2284h	accccaaugagcuuuugacc
bta-miR-2284h-3p	aaacccaaacaaauuuuuug
bta-miR-2284h-5p	aaaaguucguucggguuuuu
bta-miR-2284i	aaacccggaauuaacuuuuug
bta-miR-2284j	aaaaguucguucagguuuuuc
bta-mir-2284k	gaaaaguucggucggguuuuu
bta-mir-2284l	aaaaguugguucggguuuuu
bta-miR-2284m	gaaaaguuuguucggguuuuc
bta-mir-2284n	aaaaaguuuauucggguuuuu
bta-mir-2284o	aaaguucguucgggguuuuc
bta-miR-2284p	ugaaaguuuguucgggauuuu
bta-miR-2284q	aaaaguucguucggauuuuucc
bta-mir-2284r	uuggcccaaaaguucguucggau
bta-mir-2284s	uggcccaaaaguuuguucggau
bta-mir-2284u	aaaaguucguucgguuuuucu
bta-mir-2284v	aaaaaguucguuuggggguuu
bta-miR-2284w	aaaaccucaaugaacucuuugg
bta-miR-2284x	ugaaaaguucguucggguuuuu
bta-mir-2284y-4	aaaaguucguucggguuuuu

bta-mir-2284z-7	aaaaaaguuuguuuggguuuuu
bta-miR-2285a	aaaacugaaugaaauucuuggu
bta-miR-2285aa	aaaacuggaacgaacuuuuggg
bta-miR-2285ab	aaaaccugaaugaacuucuugg
bta-miR-2285ac	aaaaccugaagagacuuuuugg
bta-miR-2285ad	ugaaaaguucguuuggguuua
bta-miR-2285ae	aaaaacuugaacggacuuuu
bta-mir-2285af-2	gccaaaaagcuuguucagguu
bta-mir-2285ag	uccaagugaacuuuuuggcuaa
bta-miR-2285ag-3p	uccaagugaacuuuuuggcuaa
bta-mir-2285ah	uuggggguagaggugggcagga
bta-miR-2285ah-5p	ucgugcccacgcuauccuccagg
bta-miR-2285ai-3p	aaaaaccugagugaacuuuuca
bta-miR-2285ai-5p	aaauguucgcuuggcuuuuuc
bta-mir-2285aj	ugaaaaguucguuuggguuuuu
bta-miR-2285aj-5p	gcugaaaaguucguuuggguuu
bta-miR-2285ak-3p	aaaacccaaacaaaucuuuugg
bta-miR-2285ak-5p	aaagguucguucggguuuuc
bta-miR-2285al-3p	acaaaguuuguucggguuuua
bta-miR-2285al-5p	aaacccaaaugaacuuuuug
bta-miR-2285am-3p	aaaaaguuaguuuagguuuuuc
bta-miR-2285am-5p	aaaaccugaaugaacuuuuugg
bta-miR-2285an	aaagcccgaaugaacuuuuuag
bta-miR-2285ao	aaacccagacgaacuuuuug
bta-mir-2285ao-3	aaaaaguucguucggguuuucu
bta-mir-2285ap	aaacccggaugaacuuuuuggc
bta-miR-2285aq	aagcccagaugaacuuuuuggc
bta-mir-2285ar	aaacccugaugaacuuuuug
bta-mir-2285as-3	aagcccugaaugaacuuuuugg
bta-miR-2285at	aaaacccgaaggaacuuuuug
bta-mir-2285at-4	aaaacccgaaggaacuuuuug
bta-mir-2285au	aaaaaguucguucggguuuucu
bta-miR-2285av	aaaaguucguucggguuuuu
bta-miR-2285aw	aaaccugaacaaacuuuuuggc
bta-mir-2285ax-3	aaaacuugaaugaaccuuuugg
bta-mir-2285ay	aaacccugaaugaaccuuuugg
bta-mir-2285az	aaaauccgagugaacuuuuugg
bta-miR-2285b	aaaaucugagugaacuuuuugg
bta-mir-2285b-2	aaaaucugagugaacuuuuugg
bta-miR-2285ba	aaacccugaacgaacuuuuugg
bta-mir-2285ba-2	aaacccugaacgaacuuuuugg
bta-miR-2285bb	gaaaguuuguuggguuuuu
bta-miR-2285bc	aaaaaccugaaugacccuuuug

bta-miR-2285bd	aaacccugaugaacuuuuggc
bta-miR-2285be	aaaacucgagcaaacuuuuugg
bta-mir-2285bf-3	aaaacacgaacaaacuuuuug
bta-miR-2285bg	aaaaguucguuuagguuuuuc
bta-miR-2285bh	aaagguucguucggguuuu
bta-mir-2285bi-2	aagcccagaugaacuuuuuggc
bta-mir-2285bj-2	caacccugaaugaacuuuuugg
bta-mir-2285bk	aaaaguucguuugaguuuuucu
bta-mir-2285bl	aaaaccugaaugaacucuuuggu
bta-miR-2285bm	aaaaaaccgaguggacuuuuug
bta-mir-2285bn	aaaaccugaaugaacuuuuugu
bta-miR-2285bo	ugaaaaguucguucgaguuuuc
bta-miR-2285bp	aaaaaccagaacgaacuuug
bta-mir-2285bq	aaaacccgaaugaacuucuuggc
bta-mir-2285br	aaaaccugaaugaacuuucugu
bta-miR-2285bs	aaaaguuuguuugguuuuu
bta-miR-2285bt	aaaaaguucauuugguugucu
bta-miR-2285bu	aagaccugaaugaacuuuuugg
bta-mir-2285bu-2	aagaccugaaugaacuuuuugg
bta-miR-2285bv	aauguuuguuuggauguuuugu
bta-miR-2285bw	aaaaccgaaaugaacuuuuug
bta-miR-2285bx	aaaacccgaaggaacuuuuuugg
bta-miR-2285by	aaaaguuugguugguuuuucu
bta-miR-2285bz	aaaacuugaaugaacuuuuugg
bta-miR-2285c	gaaaccugaacaaacuuuuugg
bta-miR-2285ca	aagggaccugaaugaacuu
bta-miR-2285cb	aacacccggaugaacuuuugggc
bta-mir-2285cc	aaaaaccugaaugaaccuuuuga
bta-mir-2285cd	aacaaccuggaugaacuuuuugg
bta-mir-2285ce	gaaaaccugaaugaacuuuuuga
bta-mir-2285cf	caaaguucguucggguuuuuc
bta-miR-2285cg	ugaaacuuuugagccaaccca
bta-mir-2285ch	aucaaugaacuuuuuggccagc
bta-miR-2285ci	aaaaguucauucggguuuuc
bta-mir-2285cj	aaaacuggaaugaacuuuuugu
bta-miR-2285ck	uggccaaaaucuuuguuucagu
bta-miR-2285cl	uaaaguucauucggguuuu
bta-mir-2285cm	aaaaccugaacaaacuuuuug
bta-mir-2285cn	aaacccaaaugaacuucuuugg
bta-mir-2285co	aaaaccugaaugaacucuuugg
bta-mir-2285cp	aaaaguucguuuagguuuuucu
bta-miR-2285cr	aaaaaguucguucagguuu
bta-mir-2285cr-2	aaaaguucguucagguuucu

bta-mir-2285cs	aaaacccugaaugaacuuuuuga
bta-mir-2285ct	gaaaguuaguuuggauuuuucu
bta-mir-2285cv-2	gaaaauccaaaugaacuuuuuu
bta-mir-2285cw-2	aaaaguuaguucagguuuuucu
bta-mir-2285cx	aacacccaaaugaacuuuuuga
bta-mir-2285cy	aaaaccagaaugaacuuuuuug
bta-mir-2285cz	caaacccgaaugaauuuuuugu
bta-mir-2285d	aaaccugaacgaaauuuuuggc
bta-mir-2285da	gaaaaccugaaugaacuuuuuu
bta-mir-2285db	aaaaguucguucagauuuuucu
bta-mir-2285dd	gaaaaccugaaugaacuuuuua
bta-mir-2285de	aaaaguucuuucagguuuuucu
bta-miR-2285df	aaaaaguuuguuuggguu
bta-miR-2285dg	gaaaaaccgaaaugaacuuu
bta-miR-2285dh	aaggacccaaaugaacuuuuu
bta-miR-2285di	aacccugaaggaacauuuugg
bta-mir-2285dj	gaaaguuuguucagguuuguccc
bta-mir-2285dk	ccugaaugaacauuuuggccaacu
bta-mir-2285dl-2	auaaccugaaugaacuuuuugg
bta-miR-2285dl-3p	aauaaccugaaugaacuuuu
bta-miR-2285dl-5p	aaagaguucauucagguuuuc
bta-miR-2285dm	aaaacucaaacucuuuggccaac
bta-miR-2285e	aaaaccugaacgaacuuuuugg
bta-mir-2285e-2	aaaccugaacgaacuuuuuggc
bta-miR-2285f	aaaaccugaaugaacuuuuugg
bta-miR-2285g	aaaccugaacaagcuuuuuggc
bta-miR-2285h	gaaaaccgaaacgaacuuuaugg
bta-miR-2285j	aaaaaccagaacgaacuuuuug
bta-mir-2285j-2	aaaaaccagaacgaacuuuuug
bta-miR-2285k	aaaaccggaaugaacuuuuug
bta-mir-2285k-5	aaaaccggaaugaacuuuuug
bta-mir-2285l	aaaacccgcaugaacuuuuuggc
bta-mir-2285m-5	aaaacgcaaaugaacuuuuugg
bta-miR-2285n	aaaacccgaaugaacuuuuggga
bta-mir-2285n-7	aaaaacccgaaugaacuuuugg
bta-miR-2285o	gaaacccgaacgaacuuuug
bta-miR-2285p	aaaaacuugagugaacuuuugg
bta-miR-2285q	aaggaccugaaugaacuuuc
bta-mir-2285r	agaaaccuggaugaacuuuuugg
bta-miR-2285s	aagacccaagugaacucuuugg
bta-miR-2285t	agaaucuggaugaacuuuuugg
bta-mir-2285u	aaacccgaacgaacuuuuug
bta-miR-2285v	agaaccggaacgaacuuuuu

bta-mir-2285x	gaaaaaucugaaugaacuuuugg
bta-mir-2285y	aaaacccaaacaaacuuuuuu
bta-mir-2285z	aaaaccugaaugaacuuuuugg
bta-mir-2288	agguaguaggugugugguuu
bta-miR-2289	ucguguucgagcucgcgucc
bta-miR-2295	ucgggguggggaggaagguu
bta-miR-2299-3p	acuggauucauuucucugaaagu
bta-miR-2299-5p	guccggggaauggauccagcgu
bta-miR-2300a-5p	ucagcuaguuuugucucccu
bta-miR-2300b-3p	gggagacaaaacuagcug
bta-mir-2304	gguugugugguugugugugu
bta-miR-2308	uugggcuugcagcagagag
bta-mir-2309	ugugagggugguggacggcaggg
bta-mir-2310	guuuuguuuggguuuguuucu
bta-miR-2311	uacugaaacugugcucguggu
bta-mir-2312	aaaaccugaacgaacuuuuc
bta-miR-2313-3p	agugcagcugaggaccaaggcagg
bta-miR-2313-5p	ccaguucuacgcugcaugccg
bta-miR-2316	acuccggccuggacugcggcg
bta-miR-2320-3p	uggcacaggguccagcugucggc
bta-miR-2320-5p	ucgaugauggucccuguguuuug
bta-miR-2323	ugaccugaucaugcuuacugagc
bta-miR-2324	uuggggguuggggcaguguggcu
bta-miR-2328-5p	ucugcgucuggggagggggccag
bta-mir-2329-2	ucugugaugugagcugauaagu
bta-miR-2329-3p	ugaucugugaugugagcuga
bta-miR-2329-5p	ucugaucugugaugugagc
bta-miR-2330-5p	ugggcuugggcagugaggac
bta-miR-2331-3p	ggcuuccgugccugcagauguc
bta-miR-2331-5p	gcuuccgugccugcagaugucu
bta-mir-2332	cgguuuaaggucuuggagacaaag
bta-miR-2333	agcaaugcagacuccagggc
bta-miR-2336	cuaaccguaacuuugaagugcu
bta-miR-2338	aggcugaggacaggggcugacgu
bta-miR-2339	ucacuggaccuagcagagcuc
bta-mir-2340	ggacuucccugguggucuugug
bta-miR-2342	ucugcggaaguuggcgcuuuc
bta-miR-2343	aagggaaggggagacggugg
bta-miR-2346	acugaugugaagguggguuugg
bta-mir-2349	uggcacuucuggucucagacuca
bta-miR-2350	cagcuccuguuucucuccuc
bta-miR-2353	uagccugcaguacagaggacugg
bta-miR-2355-5p	auuguccuugcuguuugg

bta-mir-2357	uuggucuggcacagagcaugugag
bta-miR-2358	accaaauaguugggaucgug
bta-miR-2360	gcgggcaggaggcuggaggcguu
bta-miR-2364	cucuguggauggaugugggcaguug
bta-miR-2366	ugggucacagaagagggucugg
bta-miR-2367-5p	acccuguaacucagccaucagagu
bta-miR-2368-3p	cagacugucagaccaccucugcc
bta-miR-2368-5p	gacugucagaccaccucugc
bta-miR-2370-3p	ugggaaaaugaaaguugguuau
bta-miR-2370-5p	uaagcaacuuuccuuucuccacc
bta-miR-2372	agaaugaugaaaggauugg
bta-mir-2373	cuuccccggucagggcucggc
bta-miR-2376	ccucugagaucuugcuaggcgcu
bta-miR-2377	acugcacagaccuaggcucu
bta-miR-2378	uguggguugucugagcaagggu
bta-miR-2380	gugacuguacuuuaaaagguua
bta-mir-2381	caggcugcucugugcuuggcu
bta-miR-2382-3p	uacuuccuggugccucgccccaca
bta-miR-2382-5p	aggggagugccugggaag
bta-miR-2387	uggaaggccuggcuuugcagcgu
bta-miR-2388-3p	cucgaggaagcuggagaugacu
bta-miR-2388-5p	agcucccgucuccucugugcug
bta-miR-2389	gaggggacucgggagucggagc
bta-miR-2392	auggaugggggugaggggu
bta-miR-2396	ucaugcacugggggucuugaugg
bta-miR-2397-3p	cagagcaguggcaagaacgcauc
bta-miR-2397-5p	cagagcaguggcaagaacgcau
bta-miR-2398	aucuagggacgcgucggcaac
bta-mir-2399	uucuaacaacuacagaaagugu
bta-miR-2399-5p	cuuucuguagcuguuggaacu
bta-miR-23a	aucacauugccagggauuu
bta-mir-23b	aucacauugccagggauuaccac
bta-miR-24	uggcucaguucagcaggaac
bta-miR-2400	cccgagcgcauguggcugagu
bta-miR-2402	auaccugccacucugccagca
bta-miR-2403	cucgggaagcuagcuggccuugu
bta-mir-2404-2	uugcacugcaugguaucugc
bta-miR-2406	agggaagaaggaagcuggauu
bta-miR-2407	gcugggcggaugggaagg
bta-miR-2408	cacgugugugagcucagccggc
bta-miR-2409	ucucacauggacgcugcccugg
bta-mir-2410	cugagcugaggaggaccuguga
bta-miR-2411-3p	ugaacugucuuacucccacaucc

bta-miR-2411-5p	uggagugacugucagaugcagcc
bta-miR-2415-5p	ccaggccugcuggaccgacg
bta-miR-2416	ugcagugcccacuguggaagaug
bta-miR-2417	aauuuucagugccuccaucuag
bta-miR-2419-3p	acaggcgggugcuaauacgau
bta-miR-2419-5p	aucgcaucaacacucgucugu
bta-miR-2425-5p	ccagggcacggauccaugaacu
bta-miR-2426	ugacaccgguccacuuccuu
bta-mir-2427	aggucauuucaaagagggcug
bta-miR-2428	cuggagugcggcggaggugagc
bta-miR-2431-3p	cacccccacuugcaugacccuga
bta-miR-2431-5p	caggucauauaaguguggaguu
bta-miR-2432	aggauucuaggcuuagagguc
bta-miR-2435	aaacucgaaugaacucuuugg
bta-mir-2436	cggcgcagcugucgggagggcg
bta-miR-2436-5p	uccccgcccgaccgccucgcc
bta-miR-2438	cugguccuguacccacccugc
bta-miR-2439-5p	uaucagaagacagguaggca
bta-miR-24-3p	uggcucaguucagcaggaac
bta-mir-2440	ugcagugaugagacccugga
bta-miR-2441	acaggacaggacagugga
bta-mir-2442	agagcaggggcugugggcugca
bta-miR-2443	ugagggcaggaccguaugaggu
bta-miR-2446	uaaaauauauuugaaaucgggcc
bta-miR-2447	ucugggaaccgguuuggcugc
bta-miR-2448-3p	gugguugauuggauccgugggu
bta-miR-2448-5p	gugguugauuggauccgugggu
bta-miR-2449	ugggcaggagacagcagggc
bta-miR-2450a	caugggcaguagaggugcaugu
bta-mir-2450c	cacgucaguagaggcgcgugug
bta-mir-2451	augaggugcucuggagagugga
bta-miR-2453	uccucagggcaggaagugcgcag
bta-miR-2454-3p	ucuccucuggccgcucuccucc
bta-miR-2454-5p	ucuccucuggccgcucuccu
bta-miR-2455	cucugugcucggggaggc
bta-miR-2457	uugccaacugcagagccgcgc
bta-miR-2458	auuggaaagaguaagcuggugu
bta-mir-2459	gccagaugaugggagcugauu
bta-miR-24-5p	gugccuacugagcugaaacaca
bta-miR-2460	uggagcucuugaggccuggc
bta-miR-2461-5p	ucagacugagagcagugugcc
bta-miR-2462	aaaggauuggcugaauuguagg
bta-mir-2463	cugcaugugguggaaaugucug

bta-mir-2464	cuccagagcugagcagaacug
bta-miR-2464-5p	acuucugcugcagaccuggaaga
bta-miR-2465	uugagccacaguagagccuugga
bta-miR-2466-5p	cugaguccgguggggcagugugu
bta-miR-2467-5p	caccccaagccuggcugcuacc
bta-miR-2468	auaggaacauggaagauuguc
bta-miR-2469	agggccugcggcuuccgc
bta-miR-2472	caggguuugggauucaggu
bta-miR-2474	uaccgggccagcuggaaggaga
bta-miR-2475	ugaagugugugaaacugcagcgg
bta-miR-2478	aucccacuucugacacca
bta-miR-2483-3p	cgucaaccauccagcuguuuga
bta-miR-2483-5p	uccgucaaccauccagcuguuu
bta-miR-2484	gagcuaugaugacuuugauu
bta-miR-2485	uuucuagaaucugcagguagu
bta-miR-25	cauugcacuugucucggucuga
bta-miR-26a	uucaaguaauccaggauaggcu
bta-miR-26b	uucaaguaauucaggauagguu
bta-miR-27a-3p	agggcuuagcugcuugugagca
bta-miR-27a-5p	uucacaguggcuaaguuccg
bta-miR-27b	uucacaguggcuaaguucug
bta-miR-28	cacuagauugagagcuccugga
bta-miR-2881	ucugugaacucugagccgugguggc
bta-miR-2885	gcggcggcagcgccggggcggu
bta-mir-2887-2	cgggaccggggguccggugcg
bta-miR-2890	ccgggaaaggugggcugggcu
bta-miR-2903	uuccgcgcucuacgccagcc
bta-mir-2904-3	gggagccucgguuggccuc
bta-miR-296-3p	aggguugggcggaggcuuucc
bta-miR-296-5p	gagggcccccccaauccugu
bta-miR-299	uaugugggacgguaaacc
bta-miR-299-2	uaugugggacgguaaacc
bta-miR-29a	uagcaccaucugaaaucgguu
bta-miR-29b	cuagcaccauuugaaaucagug
bta-mir-29c	ugaccgauuucuccuggugu
bta-miR-29d-3p	uagcaccauuugaaaucga
bta-miR-29d-5p	ugaccgauuucuccuggugu
bta-mir-29e	uagcaucauuugaaaucaguguuu
bta-miR-301a	gcucugacuuuauugcacu
bta-miR-301b	cagugcaaugauauugucaaagc
bta-mir-3064	uugccacacugcaacaccuuaca
bta-miR-3065	ucaacaaaaucacugaugcugga
bta-miR-30a-3p	cuuucagucggauguuugcagc

bta-miR-30a-5p	uguaaacauccucgacuggaagcu
bta-miR-30b-3p	cugggagguggauguuuacu
bta-miR-30b-5p	uguaaacauccuacacucagcu
bta-mir-30c	uguaaacauccuacacucucagc
bta-miR-30d	uguaaacauccccgacuggaagcu
bta-miR-30e-3p	cuuucagucggauguuuacagc
bta-miR-30e-5p	uguaaacauccuugacuggaagcu
bta-miR-30f	uguaaacacccuacacucucagc
bta-miR-31	aggcaagaugcuggcauagcugu
bta-mir-3141	gagggcggguggaggagg
bta-miR-32	uauugcacaugacuaaguugca
bta-miR-320a	aaaagcuggguugagagggcga
bta-mir-320b	agcuggguugagaggguggu
bta-mir-323	gcacauuacacggucgaccucu
bta-mir-323b	cacaauacacggucggccucu
bta-miR-323b-3p	cacaauacacggucggccucu
bta-miR-324	acugccccaggugcugcuggg
bta-miR-326	ccucugggcccuuccuccagc
bta-miR-328	cuggcccucucugcccuuccgu
bta-miR-329a	aacacaccugguuaaccuuuu
bta-miR-329b	aacacaccugguuaaccucuu
bta-miR-330	gcaaagcacacggccugcagaga
bta-mir-331	ucuagguaugguccccagg
bta-miR-331-3p	gccccugggccuauccuagaac
bta-miR-331-5p	ucuagguaugguccccagggauc
bta-miR-335	uuuuucauuauugcuccugacc
bta-miR-338	aacaauauccuggugcugagu
bta-miR-339b	ucccuguccuccaggagcuca
bta-mir-33a	gugcauuguaguugcauugca
bta-mir-33b	gugcauugcuguugcauugc
bta-miR-340	uuauaaagcaaugagacugauu
bta-miR-342	ucucacacagaaaucgcacccauc
bta-miR-3431	ccucagucagccuuguggaugu
bta-miR-3432a	ugcgggaucuuuaguuguggug
bta-mir-3432b	uucgggaucuuuaguuguggu
bta-mir-345	ccugaacuaggggucuggag
bta-miR-345-3p	gcugacuccuaguccagugcu
bta-miR-345-5p	cccugaacuaggggucuggagg
bta-miR-346	ugucugcccgcaugccugccucuc
bta-miR-34a	uggcagugucuuagcugguuguu
bta-mir-34b	aggcaguguaauuagcugauug
bta-mir-34c	aggcaguguaguuagcugauug
bta-mir-3533	augaagugugacguggacau

bta-miR-361	ucccccaggugugauucugauu
bta-miR-3613a	acaaaaaaaaaaagcccaacccu
bta-miR-362-3p	aacacaccuauucaaggauu
bta-miR-362-5p	aauccuuggaaccuaggugugagu
bta-miR-363	aauugcacgguauccaucugcg
bta-mir-365-1	agggacuuuugggggcagaugug
bta-miR-365-3p	uaaugccccuaaaaauccuuau
bta-miR-365-5p	uaaugccccuaaaaauccuuau
bta-mir-369	aucgaccguguuauauucgc
bta-miR-369-3p	aauaauacaugguugaucuuu
bta-miR-369-5p	agaucgaccguguuauauucg
bta-miR-370	gccugcugggguggaaccuggu
bta-miR-374a	uuauaauacaaccugauaagu
bta-miR-374b	auauaauacaaccugcuaagu
bta-miR-374c	uuacaauacaaccugauaagugc
bta-miR-375	uuuguucguucggcucgcguga
bta-miR-376a	aucauagaggaaaauccacgu
bta-miR-376c	aacauagaggaaaauccacauu
bta-miR-378	acuggacuuggagucagaaggc
bta-mir-378b	acuugacuuggagucagaaggc
bta-mir-378c	acuggacuuggagucagaagu
bta-mir-378d	cuggacuuggagucagaagacc
bta-miR-379	ugguagacuauggaacguagg
bta-miR-380-3p	augguugaccauagaacaugcg
bta-miR-380-5p	uauguaaugugguccacgucu
bta-miR-381	uauacaagggcaagcucucugu
bta-miR-382	gaaguuguucgugguggauucg
bta-miR-383	agaucagaaggugauuguggcu
bta-mir-3956	acguggaugcugaaggucagagg
bta-miR-3957	acgcacagcaccucacugagcucc
bta-miR-409a	gaauguugcucggugaacccc
bta-miR-410	aauauaacacagauggccugu
bta-miR-411a	auaguagaccguauagcgua
bta-miR-411b	uggucgaccauaaaacguacg
bta-mir-411c	gguugaucagagaacauacauu
bta-miR-411c-3p	uguaugucaacugauccacagu
bta-miR-411c-5p	gguugaucagagaacauacau
bta-miR-412	uggucgaccaguuggaaag
bta-miR-421	aucaacagacauuaauugggcgc
bta-miR-423-3p	agcucggucugaggccccucagu
bta-miR-423-5p	ugaggggcagagagcgagacuuu
bta-miR-424-3p	caaaacgugaggcgcugcuaua
bta-miR-424-5p	cagcagcaauucauguuuuga

bta-miR-425-3p	caucgggaaugucguguccgcc
bta-miR-425-5p	aaugacacgaucacucccguugagu
bta-miR-4286	accccacuccugguacca
bta-miR-429	uaauacugucugguaaugccg
bta-mir-432	ucuuggaguaggucauugggugg
bta-mir-433	aucaugaugggcuccucggugu
bta-miR-4444	ugaacucgaguuggaagaggcga
bta-miR-4449	cccucggcggccugggagggacgg
bta-miR-449a	uggcaguguauuguuagcuggu
bta-mir-449b	aggcaguguauuguuagcuggc
bta-mir-449d	gaaggcugugugcuguggag
bta-miR-450a	uuuugcgauguguuccuaaua
bta-miR-450b	uuuugcaauauguuccugaauau
bta-mir-451	aaaccguuaccauuacugaguuu
bta-miR-452	uguuugcagaggaaacugaga
bta-miR-4523	gaccgagagggccucggcugu
bta-miR-454	uagugcaauauugcuuauaggguu
bta-miR-455-3p	ugcaguccaugggcauauacacu
bta-miR-455-5p	uaugugccuuuggacuacaucgu
bta-mir-4657	aauguggaaguggucugaggcau
bta-mir-4680	agaacucuugcagucuuagaugu
bta-mir-484	ucaggcucaguccccucccgau
bta-miR-485	agaggcuggccgugaugaauu
bta-miR-486	uccuguacugagcugccccgagg
bta-miR-487a	gugguuaucccugcuguguu
bta-miR-487b	aaucguacagggucauccacu
bta-miR-490	caaccuggaggacuccaugcug
bta-miR-491	aguggggaacccuuccaugagg
bta-miR-493	uuguacaugguaggcuuucauu
bta-miR-494	ugaaacauacacgggaaaccucu
bta-miR-495	aaacaaacauggugcacuucuuu
bta-miR-497	cagcagcacacugugguuuguac
bta-miR-499	uuaagacuugcagugauguu
bta-miR-500	aaugcaccugggcaaggauucu
bta-miR-502a	aaugcaccugggcaaggauuc
bta-mir-502b	aauccaccugggcaaggauuc
bta-miR-503-3p	uagcagcgggaacaguacug
bta-miR-503-5p	uagcagcgggaacaguacug
bta-miR-504	agacccuggucugcacucuguc
bta-miR-505	gucaacacuugcugguuuccucu
bta-miR-507-3p	auuggcaugucuuggaaugaa
bta-miR-507b	ugauugguacuucuuagagugga
bta-miR-532	caugccuugaguguaggaccgu

bta-miR-539	ggagaaauuauccuuggugugu
bta-miR-541	aaaggauucugcugucgguc
bta-miR-542-3p	ucggggaucaucaugucacg
bta-miR-542-5p	ugugacagauugauaacuga
bta-miR-543	aaacauucgcggugcacuucuuu
bta-mir-545	aucaacaaacauuuauugugug
bta-miR-545-5p	ucaacaaacauuuauugugugccu
bta-miR-574	cacgcucaugcacacacccaca
bta-miR-582	uacaguuguucaaccaguuacu
bta-miR-592	uugugucaauaugcgaugaugu
bta-miR-599	uuugauaagcugacaugggac
bta-miR-6119-3p	caaaucauuuuuuacucuccaa
bta-miR-6119-5p	agagguaaaaaauugauuugacu
bta-miR-6120-3p	cuguucccguuuuucacaugugu
bta-miR-6120-5p	uauguuggacaacguggauagc
bta-miR-6123	ugccaagcccacguucaaagg
bta-miR-615	uccgagccugggucucccu
bta-miR-628	augcugacauauuuacuagaggg
bta-mir-6501	ccagggcagccugugguaacagu
bta-miR-6516	gaucauguaugauacugcaaaca
bta-miR-6517	ucucagggucegugagcuccucg
bta-miR-6518	ucacggagaggagaaacugc
bta-mir-6519	uagcagcgggaacagagaaa
bta-miR-652	aauggcgccacuaggguugug
bta-miR-6520	ugaguauugucagagagagcgau
bta-miR-6521	guggcgccagcucugagcagagu
bta-miR-6522	ucggaauuguuuguguaccugu
bta-miR-6523a	ucuggguaacuuugagcagg
bta-miR-6524	uuacucugaguaaccuaacug
bta-miR-6525	cuggggaaagcaggagugagg
bta-mir-6527	cacguggacgaagagauggga
bta-miR-6529a	gagagaucagaggcgcagagu
bta-mir-6530	cgcggaggaagguaagacgg
bta-mir-6531	uagacuugguguacuuugagaga
bta-miR-6532	ugcggacaggaaagacgguu
bta-mir-6533	ugcggcagagcauuagagaac
bta-miR-6534	ucuaagagcagucaggacggauu
bta-mir-6535	uagggggagcgguuggaggacu
bta-miR-6536	uuuaaguauacgaugaacugca
bta-miR-654	uaugucugcugaccaucacc
bta-miR-655	auaauacaugguuaaccucucu
bta-miR-656	aauauuauacagucaaccucu
bta-mir-658	ggcggagggaagcggguccguuggu

bta-miR-660	uacccauugcauaucggagcugu
bta-miR-664a	caggcuggggugugugugg
bta-mir-664b	uauucauuuaucucccagccuac
bta-miR-665	accaguaggccgaggccccucac
bta-miR-671	uccgguucucagggcuccacc
bta-miR-6715	ccaaaccaguugugccuguaga
bta-mir-677	cucacugaugagcagcuucugac
bta-miR-6775	cauggucccuguucucugcccca
bta-miR-7	uggaagacuagugauuuuguugu
bta-miR-708	caucuagacugugagcuucu
bta-mir-7180	uguggccucuggguguguacccuc
bta-miR-744	ugcggggcuagggcuaacagc
bta-miR-758	uuugugaccugguccacuaacc
bta-miR-760-3p	ccccucaguccaccagagcccggauacc
bta-miR-760-5p	cggcucugggucugugggggagc
bta-miR-769	ugagaccucccggguucugagcu
bta-miR-7857-3p	auuguucuccaaccuggcucuuu
bta-miR-7857-5p	uagccaguuggggaagaaugc
bta-miR-7858	uaggauuuggaaagauugcu
bta-miR-7859	aaaaacuggcagcuucaugu
bta-miR-7860	uggagcggcugcacagagcgug
bta-miR-7861	cacacucagauaaaguaggcacu
bta-miR-7862	cacagcuccaggggacgccguuc
bta-mir-7863	auggacugucaccugaggagc
bta-miR-7864	cucugcugacuuugaaggugg
bta-miR-7865	cugacugccgccccccgcagg
bta-mir-8549	gagguaguagguggguau
bta-mir-8550	caggcucuggaacacgggagc
bta-mir-874	cugcccuggcccgagggaccga
bta-miR-877	guagaggagauggcgcagggg
bta-miR-885	uccauuacacuacccugccucu
bta-miR-92a	uauugcacuugucccggccugu
bta-miR-92b	uauugcacucgucccggccucc
bta-miR-93	caaagugcuguucgugcagguag
bta-miR-935	caguuaccgcuuccgcuaccg
bta-miR-9-3p	ucuuugguuaucuagcuguauga
bta-miR-95	uucaacggguauuuauugagc
bta-miR-9-5p	auaaagcuagauaaccgaaagu
bta-miR-96	uuuggcacuagcacauuuuugcu
bta-miR-98	ugagguaguaaguuguauuguu
bta-mir-9851	uggcaccagcacuggcggugg
bta-miR-99a-3p	caagcucgcuucuaugggucugu
bta-miR-99a-5p	aacccguagauccgaucuugu

bta-miR-99b	cacccguagaaccgaccuugcg

Supplemental Table 2. The predicted novel miRNAs from dry secretions

Novel miRNA	Consensus sequence	Mature read count	Chromosome/scaffold
bta-novel-miR1	ucccugcccuccaggagccca	3,353	1
bta-novel-miR2	cgcggggugggagcagcuc	625	1
bta-novel-miR3	gcaugggugguucagugguagaagu	12,109	2
bta-novel-miR4	cguggacugugugaggcc	2,718	2
bta-novel-miR5	gagaagccggcgggcgcg	349	2
bta-novel-miR6	aaaaccucaacgaacucuuugg	600	2
bta-novel-miR7	agaguucccugaacagcaagga	1,504	4
bta-novel-miR8	gcgcgcgccgcggcugga	1,150	4
bta-novel-miR9	agaaucuggaugaaauuuuuga	4,368	4
bta-novel-miR10	gggaagguggcucggggg	851	5
bta-novel-miR11	agugguuaggauucggcgcu	4,313	5
bta-novel-miR12	aagcugccaguugcagaa	544	6
bta-novel-miR13	aaaaagaucguucggguuuuu	5,714	6
bta-novel-miR13-1	aaaaagaucguucggguuuuu	10,539	19
bta-novel-miR14	cucuccgcggggggcggg	553	6
bta-novel-miR15	ugagauacgacugaagugacu	312	7
bta-novel-miR16	ugggcugcagugcgcuaugc	1,698	9
bta-novel-miR17	aaaacuugaaugaacuuuuugg	565	9
bta-novel-miR18	aaaaaguucguucggguuc	233	9
bta-novel-miR19	ugugacuguuccugguauuga	401	10
bta-novel-miR20	ggagguaguagguuguguu	239	10
bta-novel-miR21	gggggcccaaguccuucugauc	3,848	10
bta-novel-miR22	uuccuggucuagugguuaggauu	1,681	10
bta-novel-miR23	acaccaagcuguaggucugagcc	1,514	11
bta-novel-miR24	ggaggaggggagaggggg	1,834	11
bta-novel-miR24-1	ggaggaggggagaggggg	2,828	27
bta-novel-miR24-2	ggaggaggggagaggggg	1,848	29
bta-novel-miR25	cacugacuggcucagcgg	233	11
bta-novel-miR26	aaccgcgaguggcugaaccuug	377	11
bta-novel-miR27	acugacuggcucagcgugu	141,724	11
bta-novel-miR28	uuucuaaugagucagcucuuu	558	12
bta-novel-miR29	ccccgaguguuacagcccc	528	13
bta-novel-miR30	acucucauagggugggauuug	1,335	13
bta-novel-miR31	ucccuguggucccagugguuaggauc	6,058	13
bta-novel-miR32	aggagaggggggcucugga	233	13
bta-novel-miR33	uucucagguuggacaguccug	1,353	13
bta-novel-miR34	ugagguaguagguugugg	15,628	13
bta-novel-miR35	ucccugguggucuaguggu	427	13
bta-novel-miR36	gaaggggcugcugagcgcc	472	14
bta-novel-miR37	ugagguaguaguuugugu	2,434	14
bta-novel-miR38	gcauuggugguucagugguagaauu	350,928	15
bta-novel-miR39	ucacggagaggagaaacugcg	2,293	15
bta-novel-miR40	uagugugcuagaguccucgaag	3,654	15
bta-novel-miR41	aaaaauccgaacgaacuuu	536	16
bta-novel-miR42	ugccgaucgucaggccucgcugag	1,227	16
bta-novel-miR43	gagaagccgccgggagccccggga	486	16

bta-novel-miR44	ccgcgcccgcccgccggc	309	16
bta-novel-miR45	gaugaggcucagcgagcccuggu	1,091	17
bta-novel-miR46	gggggguauagcucaguggua	3,222	18
bta-novel-miR46-1	ggggguauagcucagugg	3,631	26
bta-novel-miR47	ugagugugugugugugagu	13,990	19
bta-novel-miR47-1	ugagugugugugugugag	1,170	26
bta-novel-miR48	gaggagaaggggcucuggg	3,207	19
bta-novel-miR49	acuggacuuggagucaga	8,233	19
bta-novel-miR50	uggauuuuuggagcaggga	51,410	19
bta-novel-miR51	ggggggccggcggcggcg	1,273	19
bta-novel-miR52	agaagccggcgggggcca	2,182	20
bta-novel-miR53	ucuuugguuaucuagcuguaug	6,323	21
bta-novel-miR54	aagguagauagaacaggucuug	66,660	21
bta-novel-miR55	aaaaguuuguucggguuuuc	687	21
bta-novel-miR56	gagcucaucagacugaug	724	21
bta-novel-miR57	gugaaauguuuaggaccacu	195,791	21
bta-novel-miR58	uacggggaagcuggggagg	621	23
bta-novel-miR58-1	uacggggaagcuggggagg	260	23
bta-novel-miR59	aggcgccgccgcccccccc	4,818	26
bta-novel-miR60	uuacucugaguggccuaacu	1,776	26
bta-novel-miR61	gaggcccccggcgccccga	241	26
bta-novel-miR62	gggggcgcgcggugcguc	1,082	27
bta-novel-miR63	aaaguucguucagguuuuucug	704	27
bta-novel-miR64	ugcgguagagcaucagagaacu	1,262	28
bta-novel-miR65	ucugguggggaaggaaggga	1,185	28
bta-novel-miR66	cuggacuggagcuccuugagg	1,854	29
bta-novel-miR67	cuuagagggacaaguggcguu	815	29
bta-novel-miR68	gucccacguugggaauugg	2,356	NKLS02000606.1
bta-novel-miR69	aucucgucugaucucggaagc	610	NKLS02000815.1
bta-novel-miR70	agagguaguaaguuguau	356	NKLS02002208.1
bta-novel-miR70-1	agagguaguaaguuguau	273	X
bta-novel-miR71	aaaaguucguucggguuu	361	X
bta-novel-miR72	aaaaguucguuuggguuuuc	1,532	X
bta-novel-miR73	ccuuggaggcgugggguuc	410	X

Start	End	Strand
53,290,937	53,291,016	+
99,229,776	99,229,848	+
27,428,895	27,428,967	-
72,887,559	72,887,626	+
106,925,171	106,925,238	+
114,749,768	114,749,828	-
9,646,179	9,646,241	+
89,038,486	89,038,560	-
97,122,231	97,122,293	-
34,032,969	34,033,039	+
114,325,903	114,325,971	+
17,718,563	17,718,600	+
92,398,700	92,398,766	+
45,341,831	45,341,890	-
115,983,639	115,983,705	+
60,742,520	60,742,585	+
2,054,445	2,054,526	+
30,373,218	30,373,277	+
95,776,317	95,776,376	+
13,239,290	13,239,353	-
27,567,832	27,567,871	-
41,330,918	41,330,969	-
42,888,512	42,888,576	+
2,568,960	2,569,027	+
30,254,683	30,254,732	-
6,224,548	6,224,594	-
37,120,794	37,120,867	-
68,281,414	68,281,476	-
72,585,221	72,585,277	-
95,956,548	95,956,615	-
32,330,927	32,330,993	+
795,246	795,292	+
10,242,604	10,242,661	+
40,116,167	40,116,239	-
42,574,042	42,574,126	-
53,904,038	53,904,098	-
55,631,831	55,631,869	-
65,122,825	65,122,897	-
892,986	893,041	+
13,823,320	13,823,385	+
1,327,753	1,327,825	-
42,378,037	42,378,090	-
43,881,418	43,881,479	-
3,159,799	3,159,851	+
4,484,507	4,484,576	+
51,865,351	51,865,419	-

79,950,418	79,950,492	-
60,624,771	60,624,833	+
53,719,304	53,719,368	-
26,484,446	26,484,510	-
17,729,207	17,729,254	-
48,308,885	48,308,942	-
26,406,707	26,406,769	-
39,161,045	39,161,086	-
43,221,914	43,221,957	+
49,127,860	49,127,917	+
23,282,164	23,282,220	-
20,839,107	20,839,163	+
23,034,426	23,034,488	+
57,156,234	57,156,287	-
66,513,063	66,513,137	-
68,645,108	68,645,164	+
27,534,902	27,534,953	+
27,534,934	27,535,006	+
961,037	961,085	+
31,451,977	31,452,033	-
35,277,932	35,278,016	-
6,221,855	6,221,912	-
15,646,583	15,646,639	-
24,750,918	24,750,977	+
33,236,707	33,236,759	-
41,900,293	41,900,355	-
44,457,083	44,457,144	-
3,017	3,090	+
22,988	23,040	+
5,875,702	5,875,744	-
22,641,832	22,641,874	+
44,781,632	44,781,688	+
104,619,211	104,619,268	+
108,256,927	108,256,967	-

Supplemental Table 3. The 398 miRNAs that have at least 10 counts in at least four cows for at leas

miRNA	Sequence	Type	cow1_D0
bta-miR-10162-5p	uggacaggccaagccgcugug	known	26.08
bta-miR-10164-3p	aacuguuaggaggcuuggcu	known	5.59
bta-let-7a-3p	cuauacaaucuacugucuuucc	known	139.72
bta-let-7a-5p	ugagguaguagguuguauaguu	known	112377.77
bta-let-7b	ugagguaguagguugugugguu	known	25300.09
bta-let-7c	ugagguaguagguuguau	known	24754.26
bta-let-7d	agagguaguagguugcauaguu	known	3327.14
bta-let-7e	ugagguaggagguuguauaguu	known	1318.94
bta-let-7f	ugagguaguagauuguauaguu	known	30318.75
bta-let-7g	ugagguaguaguuuguacaguu	known	24607.09
bta-let-7i	ugagguaguaguuugugcuguu	known	10033.60
bta-miR-100	aacccguagauccgaacuugu	known	175.11
bta-miR-101	uacaguacugugauaacuga	known	750.75
bta-miR-10167-3p	gcggguggucggggcggguc	known	0.00
bta-miR-10174-3p	uggguuccuggcaugcugauu	known	1.86
bta-miR-10174-5p	aucacauugccagggauuaccacg	known	2896.81
bta-miR-10179-5p	ucugcagggugguaggcugugg	known	52.16
bta-miR-10225a	ccgagccugacagaucacaca	known	3.73
bta-mir-10225b	ucgagccugacagaucacaca	known	3.73
bta-miR-103	agcagcauuguacagggcuauga	known	4789.52
bta-miR-106a	aaaagugcuuacagugcagguag	known	35.40
bta-miR-106b	ccgcacugugggguacuugcu	known	577.50
bta-miR-107	agcagcauuguacagggcuau	known	439.65
bta-miR-10a	uacccuguagauccgaauuugu	known	39.12
bta-miR-10b	uacccuguagaaccgaauuugu	known	20.49
bta-miR-11971	ugaggggcagagagugagaa	known	20.49
bta-miR-11973	cuugcugagugaccucccugcu	known	3.73
bta-miR-11975	gcggcggcggcgccggggcgcgc	known	13.04
bta-miR-11977	ugucucaguucagcaggaag	known	1.86
bta-miR-11978	acucuucccucccucggccggc	known	3.73
bta-mir-11980	aggcaacgggcuuggcggag	known	31.67
bta-miR-11981	cagggcgggaacgggcugcggg	known	16.77
bta-miR-11983	gaccagugcgaggccuguggga	known	5.59
bta-miR-11986b	uuuuccaaugaguuaguccuuu	known	72.65
bta-mir-11986c	gaacugaguccuuuggaaaaga	known	7.45
bta-mir-11987	cgaggaaucucugguggaggu	known	76.38
bta-mir-11988	aaggggacgacagaggaugaga	known	3.73
bta-miR-11989	cccagggauguagcuccu	known	0.00
bta-mir-11991	auguucuguaucuugauugua	known	1.86
bta-miR-11993	gaccugugcguguacacucaug	known	5.59
bta-mir-12004	cuggcucuggcucucugccuccu	known	3.73
bta-miR-12015	caggucccugccgggcggaga	known	0.00
bta-miR-12023	ucccucccgcccccccgcg	known	0.00
bta-miR-12025	ucgcgggguuuugcagaggacu	known	1.86
bta-mir-12034	ccccggggagcccggcggu	known	0.00
bta-miR-12035	acaccaggacuugucuccccaga	known	0.00

bta-miR-12042	ugugucuuuccuucugugugugcu	known	0.00
bta-miR-12057	acugggaguggaaggagag	known	0.00
bta-mir-12060	uugggagaccaggggaagacu	known	5.59
bta-miR-12061	ugugggaccugggucuugugg	known	1.86
bta-miR-122	uggagugugacaaugguguuug	known	9.31
bta-mir-1246	aauggauuuuuggagcagg	known	4377.82
bta-miR-1249	acgcccuucccccccuucuuc	known	3.73
bta-miR-125a	ucccugagacccuuuaaccugu	known	801.05
bta-miR-125b	ucccugagacccuaacuugug	known	1989.58
bta-mir-1260b	aucccaccacugccacca	known	150.90
bta-miR-126-5p	ucguaccgugaguaauaaugcg	known	24.22
bta-miR-127	ucggauccgucugagcuuggcu	known	7.45
bta-miR-1271	cuuggcaccuaguaaguacuca	known	87.56
bta-miR-128	ucacagugaaccggucucuuu	known	519.75
bta-miR-129	cuuuuugcggucugggcuugc	known	39.12
bta-mir-1296	uuagggcccuggcuccaucucc	known	3.73
bta-mir-1306	ccaccuccccugcaaacgucc	known	7.45
bta-miR-1307	acucggcguggcgucggucgugg	known	558.87
bta-miR-130a	cagugcaauguuaaaaggg	known	9.31
bta-miR-130b	cagugcaaugaugaaagggc	known	5.59
bta-miR-132	uaacagucuacagccauggucg	known	40.98
bta-mir-1343	cuccuggggcccgcacucuc	known	67.06
bta-miR-135a	uauggcuuuuuauuccuaugug	known	48.44
bta-miR-1388-3p	aucucagguuugucagcccg	known	0.00
bta-miR-1388-5p	aggacuguccaaccugagaau	known	13.04
bta-miR-139	ucuacagugcacgugucuccagu	known	3.73
bta-miR-140	uaccacaggguagaaccacgga	known	1123.33
bta-miR-141	uaacacugucugguaaagaug	known	2103.22
bta-miR-142-5p	cccauaaaguagaaagcacu	known	318.56
bta-miR-143	ugagaugaagcacuguagcuc	known	210.51
bta-miR-145	guccaguuuucccaggaaucccu	known	3.73
bta-miR-1468	cuccguuugccuguuuugcuga	known	135.99
bta-miR-146a	ugagaacugaauuccauaggu	known	91.28
bta-miR-146b	ugagaacugaauuccauaggcugu	known	495.53
bta-miR-147	gugugcggaaaugcuucug	known	24.22
bta-miR-148a	ucagugcacuacagaacuuugu	known	817868.49
bta-miR-148b	ucagugcaucacagaacuuugu	known	3517.16
bta-miR-149-5p	ucuggcuccgugucuucacu	known	50.30
bta-miR-150	ucucccaacccuuguaccagug	known	57.75
bta-miR-151-3p	ucgaggagcucacagucuagu	known	208.65
bta-miR-151-5p	cuagacugaagcuccuugagg	known	15257.18
bta-miR-152	ucagugcaugacagaacuugg	known	773.10
bta-miR-155	uuaaugcuaaucgugauaggggu	known	255.22
bta-miR-15a	uagcagcacauaaugguuugu	known	20.49
bta-miR-15b	uagcagcacaucaugguuu	known	68.93
bta-miR-16a	uagcagcacguaaauauuggu	known	337.19
bta-miR-16b	uagcagcacguaaauauuggc	known	150.90
bta-miR-17-3p	acugcagugaaggcacuugua	known	3.73

bta-miR-17-5p	caaagugcuuacagugcagguag	known	210.51
bta-miR-181a	aacauucaacgcugucggugagu	known	1479.15
bta-miR-181b	aacauucauugcugucgguggu	known	322.28
bta-miR-181c	aacauucaaccugucggugagu	known	9.31
bta-miR-181d	aacauucauuguugucgguggu	known	42.85
bta-miR-182	uuuggcaaugguagaacucacacu	known	3438.92
bta-miR-183	uauggcacugguagaauucacu	known	745.16
bta-miR-1839	aagguagauagaacaggucuug	known	1009.69
bta-miR-184	uggacggagaacugauaagggu	known	54.02
bta-miR-185	uggagagaaaggcaguuccuga	known	245.90
bta-miR-186	caaagaauucuccuuuugggcu	known	9167.35
bta-mir-188	caucccuugcaugguggagggu	known	31.67
bta-miR-18a	acugcccuaagugcuccuucu	known	13.04
bta-miR-191	caacggaaucccaaaagcagcu	known	7392.00
bta-miR-192	cugaccuaugaauugacagcc	known	566.32
bta-miR-193a-3p	aacuggccuacaaaguccc	known	9.31
bta-miR-193a-5p	ugggucuuugcgggcgagaug	known	929.59
bta-miR-193b	aacuggcccacaaagucccgcu	known	5.59
bta-miR-194	uguaacagcaacuccaugugg	known	307.38
bta-miR-195	uagcagcacagaaauauuggca	known	0.00
bta-miR-196a	uagguaguuucauguuguuggg	known	139.72
bta-miR-196b	uagguaguuuccuguuguuggg	known	1.86
bta-miR-197	uucaccaccuucuccacccagc	known	126.68
bta-miR-19a	ugugcaaaucuaugcaaaacuga	known	37.26
bta-miR-19b	ugugcaaauccaugcaaaacug	known	130.40
bta-miR-200a	uaacacugucugguaacgaug	known	26130.94
bta-miR-200b	uaauacugccugguaaugauga	known	57850.59
bta-miR-200c	uaauacugccggguaaugaugga	known	49107.99
bta-miR-205	uccuucauuccaccggagucugu	known	31.67
bta-miR-20a	uaaagugcuuauagugcagguag	known	299.93
bta-miR-20b	caaagugcucacagugcagguag	known	61.48
bta-miR-210	cugugcgugugacagcggcuga	known	83.83
bta-miR-212	accuuggcucuagacugcuuac	known	11.18
bta-miR-21-3p	caacagcagucgaugggc	known	9.31
bta-miR-215	augaccuaugaauugacagaca	known	240.31
bta-miR-21-5p	uagcuuaucagacugauguugac	known	61058.51
bta-miR-218	uugugcuugaucuaaccaugugg	known	0.00
bta-miR-221	agcuacauugucugcuggguuu	known	1017.15
bta-miR-222	agcuacaucuggcuacugggucucu	known	240.31
bta-mir-223	ugucaguuugucaaauacccca	known	154.62
bta-miR-22-3p	aguucuucaguggcaagcuuu	known	484.35
bta-miR-224	caagucacuagugguuccguuu	known	22.35
bta-miR-22-5p	aagcugccaguugaagaacugu	known	8737.02
bta-miR-2283p	aauuguucauuuggguuuuu	known	0.00
bta-mir-2284a	aaaaguucguuggggguuuuu	known	1.86
bta-miR-2284ab	uaaaaguuugguuggguuuuu	known	124.81
bta-mir-2284b	aaaaguucguuugguuuuuuc	known	5.59
bta-mir-2284d	aaaaaguucguuaggguuuuuc	known	13.04

bta-mir-2284h	accccaaugagcuuuugacc	known	29.81
bta-miR-2284j	aaaaguucguucagguuuuuc	known	204.92
bta-mir-2284k	gaaaaguucggucggguuuuu	known	9.31
bta-miR-2284m	gaaaaguuuguucggguuuuc	known	70.79
bta-mir-2284n	aaaaaguuuauucggguuuuu	known	0.00
bta-mir-2284v	aaaaaguucguuuggguuu	known	7.45
bta-miR-2284w	aaaaccucaaugaacucuuugg	known	137.85
bta-miR-2284x	ugaaaaguucguucggguuuuu	known	4808.15
bta-mir-2284y-4	aaaaguucguucggguuuuu	known	1000.38
bta-mir-2284z-7	aaaaaaguuuguuuggguuuuu	known	22.35
bta-miR-2285aa	aaaacuggaacgaacuuuuggg	known	39.12
bta-miR-2285ab	aaaaccugaaugaacuucuugg	known	3.73
bta-miR-2285ad	ugaaaaguucguuuggguuua	known	20.49
bta-miR-2285aj-5p	gcugaaaaguucguuuggguuu	known	27.94
bta-miR-2285ak-5p	aaagguucguucggguuuuc	known	0.00
bta-mir-2285ao-3	aaaaaguucguucggguuuucu	known	108.05
bta-mir-2285ap	aaacccggaugaacuuuuuggc	known	9.31
bta-mir-2285au	aaaaaguucguucggguuuucu	known	108.05
bta-miR-2285av	aaaaguucguucggguuuuu	known	0.00
bta-miR-2285b	aaaaucugagugaacuuuuugg	known	3.73
bta-miR-2285ba	aaacccugaacgaacuuuuugg	known	44.71
bta-miR-2285bb	gaaaguuuguuggguuuuu	known	29.81
bta-miR-2285bc	aaaaaccugaaugacccuuuug	known	13.04
bta-miR-2285bd	aaacccugaugaacuuuuuggc	known	0.00
bta-mir-2285bf-3	aaaacacgaacaaacuuuuug	known	0.00
bta-miR-2285bh	aaagguucguucggguuuu	known	11.18
bta-mir-2285bl	aaaaccugaaugaacucuuuggu	known	7.45
bta-mir-2285bn	aaaaccugaaugaacuuuuugu	known	7.45
bta-miR-2285bo	ugaaaaguucguucgaguuuuc	known	165.80
bta-mir-2285br	aaaaccugaaugaacuuucugu	known	0.00
bta-miR-2285by	aaaaguuugguuggguuuuucu	known	3.73
bta-miR-2285bz	aaaacuugaaugaacuuuuugg	known	0.00
bta-mir-2285cc	aaaaaccugaaugaaccuuuuga	known	7.45
bta-mir-2285cd	aacaaccuggaugaacuuuuugg	known	7.45
bta-mir-2285ce	gaaaaccugaaugaacuuuuuga	known	1.86
bta-mir-2285cf	caaaguucguucggguuuuuc	known	1.86
bta-mir-2285cj	aaaacuggaaugaacuuuuugu	known	5.59
bta-mir-2285cm	aaaaccugaacaaacuuuuug	known	7.45
bta-mir-2285cr-2	aaaaguucguucagguuucu	known	14.90
bta-mir-2285da	gaaaaccugaaugaacuuuuuu	known	1.86
bta-mir-2285dd	gaaaaccugaaugaacuuuuua	known	3.73
bta-mir-2285j-2	aaaaaccagaacgaacuuuuug	known	14.90
bta-miR-2285k	aaaaccggaaugaacuuuuug	known	63.34
bta-mir-2285k-5	aaaaccggaaugaacuuuuug	known	0.00
bta-mir-2285n-7	aaaaacccgaaugaacuuuugg	known	1.86
bta-miR-2285o	gaaacccgaacgaacuuuug	known	3.73
bta-miR-2285p	aaaaacuugagugaacuuuugg	known	9.31
bta-miR-2285q	aaggaccugaaugaacuuuc	known	7.45

bta-miR-2285t	agaaucuggaugaacuuuuugg	known	3785.42
bta-mir-2285u	aaacccgaacgaacuuuuug	known	18.63
bta-mir-2285z	aaaaccugaaugaacuuuuugg	known	35.40
bta-miR-2299-3p	acuggauucauuucucugaaagu	known	20.49
bta-miR-2299-5p	guccggggaauggauccagcgu	known	20.49
bta-miR-2311	uacugaaacugugcucguggu	known	3.73
bta-miR-2316	acuccggccuggacugcggcg	known	1.86
bta-miR-2320-5p	ucgaugaugguccuguguuuug	known	3.73
bta-miR-2329-5p	ucugaucugugaugugagc	known	0.00
bta-mir-2332	cgguuuaaggucuuggagacaaag	known	3.73
bta-miR-2336	cuaaccguaacuuugaagugcu	known	55.89
bta-mir-2340	ggacuucccugguggucuugug	known	0.00
bta-miR-2387	uggaaggccuggcuuugcagcgu	known	55.89
bta-miR-23a	aucacauugccagggauuu	known	6676.64
bta-miR-24	uggcucaguucagcaggaac	known	6028.35
bta-miR-2409	ucucacauggacgcugcccugg	known	0.00
bta-miR-2411-5p	uggagugacugucagaugcagcc	known	39.12
bta-miR-2419-3p	acaggcgggugcuaauacgau	known	9.31
bta-miR-2419-5p	aucgcaucaacacucgucugu	known	95.01
bta-miR-2426	ugacaccgguccacuuccuu	known	1.86
bta-miR-2431-5p	caggucauauaaguguggaguu	known	1.86
bta-miR-24-3p	uggcucaguucagcaggaac	known	6028.35
bta-miR-2443	ugagggcaggaccguaugaggu	known	16.77
bta-miR-2448-3p	gugguugauuggauccgugggu	known	0.00
bta-miR-2448-5p	gugguugauuggauccgugggu	known	0.00
bta-miR-24-5p	gugccuacugagcugaaacaca	known	158.35
bta-miR-2468	auaggaacauggaagauuguc	known	0.00
bta-miR-2478	aucccacuucugacacca	known	134.13
bta-miR-2483-5p	uccgucaaccauccagcuguuu	known	1.86
bta-miR-2484	gagcuaugaugacuuugauu	known	7.45
bta-miR-25	cauugcacuugucucggucuga	known	7313.76
bta-miR-26a	uucaaguaauccaggauaggcu	known	72699.79
bta-miR-26b	uucaaguaauucaggauagguu	known	6693.41
bta-miR-27a-3p	agggcuuagcugcuugugagca	known	68.93
bta-miR-27a-5p	uucacaguggcuaaguuccg	known	3742.57
bta-miR-27b	uucacaguggcuaaguucug	known	13127.88
bta-miR-28	cacuagauugagagcuccugga	known	523.48
bta-mir-2887-2	cgggaccggggucggugcg	known	3.73
bta-mir-2904-3	gggagccucgguuggccuc	known	16.77
bta-miR-296-3p	aggguugggcggaggcuuucc	known	39.12
bta-miR-296-5p	gagggcccccccaauccugu	known	104.32
bta-miR-299	uaugugggacgguaaacc	known	0.00
bta-miR-29a	uagcaccaucugaaaucgguu	known	1764.17
bta-miR-29b	cuagcaccauuugaaaucagug	known	545.83
bta-mir-29c	ugaccgauuucuccugguguu	known	491.81
bta-miR-29d-5p	ugaccgauuucuccugguguu	known	245.90
bta-miR-30a-3p	cuuucagucggauguuugcagc	known	2911.72
bta-miR-30a-5p	uguaaacauccucgacuggaagcu	known	56183.29

bta-miR-30b-3p	cugggagguggauguuuacu	known	67.06
bta-miR-30b-5p	uguaaacauccuacacucagcu	known	3900.92
bta-mir-30c	uguaaacauccuacacucucagc	known	3651.29
bta-miR-30d	uguaaacauccccgacuggaagcu	known	24823.18
bta-miR-30e-3p	cuuucagucggauguuuacagc	known	888.60
bta-miR-30e-5p	uguaaacauccuugacuggaagcu	known	1516.40
bta-miR-30f	uguaaacacccuacacucucagc	known	4420.67
bta-miR-31	aggcaagaugcuggcauagcugu	known	3.73
bta-miR-32	uauugcacaugacuaaguugca	known	217.96
bta-miR-320a	aaaagcuggguugagagggcga	known	12453.51
bta-mir-320b	agcuggguugagaggguggu	known	1.86
bta-miR-324	acugccccaggugcugcuggg	known	29.81
bta-miR-326	ccucugggcccuuccuccagc	known	83.83
bta-miR-328	cuggcccucucugcccuuccgu	known	57.75
bta-miR-330	gcaaagcacacggccugcagaga	known	20.49
bta-miR-331-3p	gccccugggccuauccuagaac	known	35.40
bta-miR-331-5p	ucuagguaugguccagggauc	known	72.65
bta-miR-335	uuuuucauuauugcuccugacc	known	3.73
bta-miR-338	aacaauauccuggugcugagu	known	22.35
bta-miR-339b	ucccuguccuccaggagcuca	known	950.08
bta-miR-340	uuauaaagcaaugagacugauu	known	685.55
bta-miR-342	ucucacacagaaaucgcacccauc	known	251.49
bta-miR-3431	ccucagucagccuuguggaugu	known	7.45
bta-miR-3432a	ugcgggaucuuuaguuguggug	known	493.67
bta-mir-3432b	uucgggaucuuuaguuguggu	known	33.53
bta-miR-345-3p	gcugacuccuaguccagugcu	known	50.30
bta-miR-345-5p	cccugaacuaggggucuggagg	known	273.85
bta-miR-34a	uggcagugucuuagcugguuguu	known	70.79
bta-mir-3533	augaagugugacguggacau	known	1.86
bta-miR-361	ucccccaggugugauucugauu	known	4523.13
bta-miR-3613a	acaaaaaaaaaagcccaacccu	known	5.59
bta-miR-362-3p	aacacaccuauucaaggauu	known	7.45
bta-miR-362-5p	aauccuuggaaccuaggugugagu	known	128.54
bta-miR-363	aauugcacgguauccaucugcg	known	223.55
bta-miR-365-3p	uaaugccccuaaaaauccuuau	known	182.56
bta-miR-365-5p	uaaugccccuaaaaauccuuau	known	182.56
bta-miR-374a	uuauaauacaaccugauaagu	known	242.18
bta-miR-374b	auauaauacaaccugcuaagu	known	1024.60
bta-miR-374c	uuacaauacaaccugauaagugc	known	0.00
bta-miR-375	uuuguucguucggcucgcguga	known	5566.35
bta-miR-378	acuggacuuggagucagaaggc	known	8332.77
bta-mir-378b	acuugacuuggagucagaaggc	known	13.04
bta-mir-378c	acuggacuuggagucagaagu	known	1490.32
bta-mir-378d	cuggacuuggagucagaagacc	known	29.81
bta-miR-379	ugguagacuauggaacguagg	known	5.59
bta-miR-381	uauacaagggcaagcucucugu	known	1.86
bta-miR-382	gaaguuguucgugguggauucg	known	9.31
bta-miR-409a	gaauguugcucggugaacccc	known	3.73

bta-miR-411a	auaguagaccguauagcgua	known	5.59
bta-miR-421	aucaacagacauuaauugggcgc	known	100.60
bta-miR-423-3p	agcucggucugaggccccucagu	known	1812.60
bta-miR-423-5p	ugaggggcagagagcgagacuuu	known	14362.98
bta-miR-424-3p	caaaacgugaggcgcugcuaua	known	3.73
bta-miR-424-5p	cagcagcaauucauguuuuga	known	7.45
bta-miR-425-3p	caucgggaaugucguguccgcc	known	130.40
bta-miR-425-5p	aaugacacgaucacucccguugagu	known	234.73
bta-miR-4286	accccacuccugguacca	known	40.98
bta-miR-429	uaauacugucugguaaugccg	known	529.06
bta-mir-449d	gaaggcugugugcuguggag	known	0.00
bta-miR-450a	uuuugcgauguguuccuaaua	known	7.45
bta-miR-450b	uuuugcaauauguuccugaauau	known	22.35
bta-mir-451	aaaccguuaccauuacugaguuu	known	1.86
bta-miR-452	uguuugcagaggaaacugaga	known	5.59
bta-miR-455-3p	ugcaguccaugggcauauacacu	known	5.59
bta-miR-455-5p	uaugugccuuuggacuacaucgu	known	24.22
bta-mir-484	ucaggcucaguccccucccgau	known	411.70
bta-miR-486	uccuguacugagcugccccgagg	known	3.73
bta-miR-490	caaccuggaggacuccaugcug	known	5.59
bta-miR-491	aguggggaacccuuccaugagg	known	5.59
bta-miR-493	uuguacaugguaggcuuucauu	known	1.86
bta-miR-494	ugaaacauacacgggaaaccucu	known	3.73
bta-miR-499	uuaagacuugcagugauguu	known	111.77
bta-miR-500	aaugcaccugggcaaggauucu	known	471.31
bta-miR-502a	aaugcaccugggcaaggauuc	known	96.87
bta-mir-502b	aauccaccugggcaaggauuc	known	29.81
bta-miR-503-5p	uagcagcgggaacaguacug	known	0.00
bta-miR-504	agacccuggucugcacucuguc	known	24.22
bta-miR-505	gucaacacuugcugguuuccucu	known	24.22
bta-miR-532	caugccuugaguguaggaccgu	known	3982.89
bta-miR-542-5p	ugugacagauugauaacuga	known	1.86
bta-miR-574	cacgcucaugcacacacccaca	known	2298.82
bta-miR-6119-3p	caaaucauuuuuuacucuccaa	known	1.86
bta-miR-6119-5p	agagguaaaaaauugauuugacu	known	46.57
bta-miR-6120-5p	uauguuggacaacguggauagc	known	113.64
bta-miR-6123	ugccaagcccacguucaaagg	known	7.45
bta-miR-615	uccgagccugggucucccu	known	7.45
bta-miR-628	augcugacauauuuacuagaggg	known	11.18
bta-miR-6516	gaucauguaugauacugcaaaca	known	14.90
bta-miR-6517	ucucagggucccgugagcuccucg	known	61.48
bta-miR-6518	ucacggagaggagaaacugc	known	3.73
bta-miR-652	aauggcgccacuaggguugug	known	1276.09
bta-miR-6520	ugaguauugucagagagagcgau	known	22.35
bta-miR-6522	ucggaauuguuuguguaccugu	known	113.64
bta-miR-6523a	ucugggguaacuuugagcagg	known	16.77
bta-miR-6524	uuacucugaguaaccuaacug	known	301.79
bta-miR-6529a	gagagaucagaggcgcagagu	known	484.35

bta-miR-660	uacccauugcauaucggagcugu	known	2848.38
bta-mir-664b	uauucauuuaucucccagccuac	known	74.52
bta-miR-671	uccgguucucagggcuccacc	known	14.90
bta-miR-6715	ccaaaccaguugugccuguaga	known	264.53
bta-miR-7	uggaagacuagugauuuuguuguu	known	1192.26
bta-miR-708	caucuagacugugagcuucu	known	5.59
bta-mir-7180	uguggccucugggugugguacccuc	known	13.04
bta-miR-744	ugcggggcuagggcuaacagc	known	83.83
bta-miR-760-5p	cggcucugggucuguggggagc	known	404.25
bta-miR-769	ugagaccuccggguucugagcu	known	63.34
bta-miR-7857-3p	auuguucuccaaccuggcucuuu	known	20.49
bta-miR-7857-5p	uagccaguuggggaagaaugc	known	31.67
bta-miR-7858	uaggauuuggaaagauugcu	known	78.24
bta-miR-7859	aaaaacuggcagcuucaugu	known	132.27
bta-miR-7860	uggagcggcugcacagagcgug	known	5.59
bta-miR-7862	cacagcuccaggggacgccguuc	known	11.18
bta-mir-7863	auggacugucaccugaggagc	known	0.00
bta-miR-7865	cugacugccgcccccgcagg	known	5.59
bta-mir-8549	gagguaguagguggua u	known	13.04
bta-mir-874	cugcccuggcccgagggaccga	known	18.63
bta-miR-877	guagaggagauggcgcagggg	known	18.63
bta-miR-885	uccauuacacuacccugccucu	known	255.22
bta-miR-92a	uauugcacuugucccggccugu	known	4905.02
bta-miR-92b	uauugcacucgucccggccucc	known	57.75
bta-miR-93	caaagugcuguucgugcagguag	known	748.89
bta-miR-9-3p	ucuuugguuaucuagcuguauga	known	22.35
bta-miR-95	uucaacggguauuuauugagc	known	13.04
bta-miR-9-5p	auaaagcuagauaaccgaaagu	known	9.31
bta-miR-96	uuuggcacuagcacauuuuugcu	known	137.85
bta-miR-98	ugagguaguaaguuguauuguu	known	1445.61
bta-mir-9851	uggcaccagcacuggcggugg	known	3.73
bta-miR-99a-3p	caagcucgcuucuaugggucugu	known	27.94
bta-miR-99a-5p	aacccguagauccgaucuugu	known	70231.45
bta-miR-99b	cacccguagaaccgaccuugcg	known	963.12
bta-novel-miR7	agaguucccugaacagcaagga	novel	29.81
bta-novel-miR8	gcgcgcgccgcggcugga	novel	0.00
bta-novel-miR17	aaaacuugaaugaacuuuuugg	novel	0.00
bta-novel-miR19	ugugacuguuccugguauuga	novel	13.04
bta-novel-miR22	uuccuggucuagugguuaggauu	novel	70.79
bta-novel-miR23	acaccaagcuguaggucugagcc	novel	27.94
bta-novel-miR24-1	ggaggaggggagaggggg	novel	63.34
bta-novel-miR27	acugacuggcucagcgugu	novel	0.00
bta-novel-miR30	acucucauagggugggauuug	novel	0.00
bta-novel-miR31	ucccugugguccagugguuaggauc	novel	111.77
bta-novel-miR33	uucucagguuggacaguccug	novel	27.94
bta-novel-miR37	ugagguaguaguuugugu	novel	167.66
bta-novel-miR39	ucacggagaggagaaacugcg	novel	0.00
bta-novel-miR40	uagugugcuagaguccucgaag	novel	0.00

bta-novel-miR42	ugccgaucgucaggccucgcugag	novel	0.00
bta-novel-miR45	gaugaggcucagcgagcccuggu	novel	0.00
bta-novel-miR46-1	gggggguauagcucagugg	novel	0.00
bta-novel-miR47-1	ugagugugugugugugag	novel	0.00
bta-novel-miR49	acuggacuuggagucaga	novel	0.00
bta-novel-miR52	agaagccggcgggggcca	novel	0.00
bta-novel-miR53	ucuuugguuaucuagcuguaug	novel	22.35
bta-novel-miR54	aagguagauagaacaggucuug	novel	1073.03
bta-novel-miR55	aaaaguuuguucggguuuuc	novel	0.00
bta-novel-miR57	gugaaauguuuaggaccacu	novel	545.83
bta-novel-miR62	gggggcgcgcggugcguc	novel	0.00
bta-novel-miR64	ugcgguagagcaucagagaacu	novel	0.00
bta-novel-miR72	aaaaguucguuuggguuuuc	novel	72.65
bta-novel-miR34	ugagguaguagguugugg	novel	281.30
bta-novel-miR70	agagguaguaaguuguau	novel	0.00
bta-novel-miR12	aagcugccaguugcagaa	novel	0.00

t one timepoint. Also shown are the normalized counts per sample (six cows four timpoints)

cow1_D3	cow1_D10	cow1_D21	cow2_D0	cow2_D3	cow2_D10	cow2_D21	cow3_D0
0.00	34.75	43.38	31.12	20.19	16.38	3.51	5.18
0.00	0.00	3.62	3.89	6.21	0.00	0.00	0.00
66.00	69.51	57.84	97.26	110.29	125.61	248.03	54.34
97581.10	136336.43	132912.32	131381.30	169127.35	155884.92	95309.59	157833.53
143748.15	98535.03	174501.42	34787.59	45035.28	88976.06	55697.15	51920.29
26702.53	20968.60	29319.16	35386.70	32820.72	17747.15	9596.58	37936.87
4004.00	3608.68	4410.53	3614.14	4222.20	3465.32	1051.84	3436.34
1380.50	1291.71	1829.28	1287.71	1506.82	1526.49	943.64	1948.47
16076.52	43506.94	24369.97	24194.14	35028.13	59972.75	44326.41	27653.73
9608.51	18286.70	10747.95	17596.09	22392.59	29164.42	31218.76	16322.61
6385.51	8509.08	10165.90	7944.11	7565.17	10267.62	6806.42	6238.71
209.00	144.81	198.84	210.08	152.24	87.38	88.53	142.32
423.50	932.58	307.29	863.66	781.37	1269.80	5624.59	644.31
5.50	0.00	0.00	3.89	0.00	24.58	28.81	0.00
11.00	0.00	0.00	27.23	9.32	0.00	0.00	2.59
2717.00	9934.02	6181.97	3007.25	3728.21	11100.50	5266.25	2393.53
77.00	17.38	75.92	77.81	0.00	21.85	9.13	59.51
11.00	5.79	0.00	7.78	20.19	27.31	3.51	12.94
0.00	0.00	7.23	0.00	6.21	8.19	0.70	7.76
4493.50	9099.91	8271.55	4598.40	5662.23	12130.00	12337.56	3291.43
27.50	69.51	39.77	3.89	21.75	81.92	211.49	7.76
555.50	492.36	484.43	758.62	661.76	660.84	721.61	610.67
324.50	712.47	589.28	322.90	414.76	625.34	498.17	201.83
0.00	0.00	3.62	31.12	4.66	16.38	3.51	2.59
16.50	0.00	47.00	120.60	49.71	40.96	68.86	49.16
44.00	11.58	50.61	31.12	13.98	32.77	3.51	2.59
0.00	5.79	10.85	19.45	7.77	2.73	6.32	2.59
5.50	11.58	0.00	27.23	7.77	5.46	4.22	25.88
5.50	23.17	10.85	3.89	4.66	21.85	5.62	2.59
5.50	0.00	14.46	62.25	62.14	2.73	2.81	12.94
913.00	110.06	101.23	50.57	29.52	704.53	55.51	12.94
27.50	0.00	10.85	31.12	27.96	8.19	2.81	31.05
0.00	23.17	10.85	11.67	4.66	8.19	2.81	0.00
33.00	5.79	10.85	31.12	57.48	35.50	25.29	12.94
5.50	11.58	47.00	7.78	0.00	40.96	14.05	2.59
275.00	382.30	488.05	85.59	73.01	210.27	42.86	54.34
22.00	17.38	7.23	3.89	4.66	24.58	0.70	5.18
0.00	5.79	0.00	11.67	0.00	5.46	4.22	0.00
0.00	5.79	3.62	3.89	1.55	0.00	14.05	0.00
0.00	5.79	3.62	11.67	3.11	0.00	2.11	2.59
0.00	0.00	7.23	0.00	0.00	0.00	1.41	15.53
0.00	17.38	7.23	0.00	0.00	0.00	1.41	0.00
0.00	0.00	0.00	0.00	0.00	13.65	1.41	0.00
0.00	0.00	3.62	11.67	0.00	0.00	0.00	0.00
38.50	0.00	10.85	0.00	1.55	5.46	0.70	0.00
11.00	0.00	0.00	0.00	0.00	2.73	0.70	0.00

11.00	0.00	3.62	0.00	1.55	0.00	0.00	5.18
5.50	11.58	25.31	0.00	0.00	5.46	0.00	0.00
0.00	23.17	32.54	0.00	0.00	8.19	0.00	5.18
11.00	0.00	0.00	0.00	1.55	2.73	0.00	0.00
0.00	11.58	3.62	70.03	24.85	0.00	4.92	18.11
42773.54	31788.86	93423.64	4380.54	534.38	7031.68	2748.00	3379.41
11.00	5.79	3.62	15.56	15.53	5.46	4.92	5.18
561.00	480.77	683.27	2003.53	2003.92	406.88	1481.15	1257.58
1292.50	978.92	1688.29	3088.94	2493.24	830.15	2266.00	1684.53
88.00	46.34	101.23	194.52	152.24	98.31	131.39	144.91
16.50	46.34	7.23	19.45	35.73	21.85	22.48	20.70
27.50	5.79	28.92	27.23	20.19	10.92	7.03	7.76
66.00	75.30	97.61	58.36	37.28	38.23	63.94	25.88
396.00	347.55	516.97	688.59	893.22	223.92	165.82	535.63
16.50	11.58	0.00	23.34	21.75	21.85	5.62	25.88
5.50	17.38	0.00	15.56	4.66	0.00	4.92	5.18
11.00	28.96	0.00	42.79	10.87	0.00	6.32	15.53
616.00	353.34	513.36	836.43	495.54	199.34	186.90	533.05
0.00	5.79	25.31	3.89	4.66	2.73	5.62	0.00
5.50	295.41	354.29	19.45	10.87	144.73	40.75	7.76
22.00	5.79	0.00	23.34	24.85	2.73	3.51	5.18
82.50	11.58	39.77	112.82	132.04	32.77	39.35	64.69
44.00	34.75	39.77	42.79	49.71	30.04	34.43	31.05
0.00	0.00	0.00	0.00	0.00	0.00	2.81	0.00
0.00	92.68	50.61	7.78	4.66	19.12	16.86	7.76
16.50	11.58	50.61	19.45	23.30	19.12	14.76	2.59
1067.00	2380.69	1793.13	1361.63	1483.52	3448.94	2707.95	776.28
1325.50	1268.54	968.87	2454.82	2485.48	2124.52	3341.03	1477.52
0.00	191.15	0.00	346.24	489.33	1346.26	0.00	28.46
143.00	144.81	137.38	252.87	475.35	330.42	242.41	59.51
5.50	5.79	0.00	15.56	15.53	0.00	2.81	2.59
258.50	92.68	202.45	213.97	194.18	106.50	101.18	194.07
0.00	57.92	39.77	46.68	31.07	109.23	196.74	5.18
478.50	1048.43	1033.94	723.61	542.14	1146.92	921.15	289.81
44.00	167.98	108.46	54.47	27.96	177.50	97.67	10.35
770517.79	678171.96	505775.38	703474.11	662084.32	496521.54	361356.60	909457.62
2271.50	2722.44	1738.90	2548.18	2584.90	2454.95	3087.38	2991.27
60.50	5.79	32.54	27.23	32.62	8.19	14.76	7.76
38.50	17.38	47.00	62.25	83.88	79.19	49.18	15.53
99.00	92.68	115.69	241.20	141.36	111.96	155.28	93.15
16808.02	10252.60	13325.58	18942.16	13848.76	8339.71	4765.27	18328.00
847.00	2021.56	1359.31	929.80	1222.54	3091.21	2795.08	680.54
286.00	220.11	339.83	447.39	377.48	710.00	496.06	173.37
0.00	11.58	18.08	11.67	20.19	73.73	45.67	10.35
11.00	11.58	79.53	81.70	43.50	174.77	73.07	18.11
181.50	144.81	220.53	315.12	380.59	576.19	786.25	150.08
99.00	69.51	90.38	120.60	173.98	215.73	255.06	54.34
0.00	5.79	3.62	15.56	9.32	8.19	7.73	0.00

170.50	527.11	404.90	241.20	281.17	704.53	1433.37	100.92
1716.00	990.51	1605.14	2194.16	2219.84	1903.33	2786.65	1348.14
418.00	469.19	498.90	462.95	459.81	497.00	678.04	279.46
0.00	0.00	3.62	0.00	1.55	5.46	12.65	0.00
93.50	46.34	83.15	38.90	15.53	27.31	37.94	12.94
3707.00	3405.95	4077.93	4209.37	3919.29	4306.39	3972.70	4303.19
616.00	903.62	1254.47	766.40	626.03	1081.38	1279.50	789.22
473.00	1077.39	856.80	1034.84	1045.45	2080.83	1090.49	784.04
49.50	63.72	213.30	50.57	26.41	30.04	14.05	7.76
467.50	868.86	860.41	280.11	419.42	903.88	354.83	188.90
6864.01	5462.26	5863.83	8562.68	9889.09	8006.56	11391.11	4003.02
0.00	52.13	25.31	11.67	0.00	21.85	10.54	10.35
11.00	40.55	18.08	31.12	1.55	16.38	8.43	7.76
7969.51	2201.12	5090.18	9935.98	10717.06	4464.78	2437.44	7180.60
275.00	700.88	665.19	731.39	823.31	882.03	994.93	432.13
0.00	0.00	0.00	3.89	7.77	0.00	6.32	0.00
913.00	1784.07	1984.74	1548.36	936.71	614.42	394.88	1032.45
0.00	11.58	7.23	0.00	0.00	0.00	4.92	2.59
324.50	376.51	401.29	420.16	444.28	617.15	683.66	175.96
0.00	0.00	0.00	0.00	3.11	0.00	4.22	0.00
231.00	191.15	198.84	287.89	293.60	420.54	409.64	271.70
5.50	28.96	21.69	15.56	18.64	32.77	38.64	2.59
27.50	23.17	32.54	248.98	217.48	35.50	51.99	124.21
11.00	11.58	0.00	15.56	35.73	21.85	36.54	18.11
60.50	23.17	18.08	108.93	114.95	65.54	277.54	36.23
20696.52	14666.43	15039.17	31453.55	33710.83	24276.38	29587.95	17981.26
51876.05	39307.43	56367.98	77853.86	72252.80	61370.90	70083.60	52142.82
55687.56	30334.96	49719.65	84027.86	77444.34	47075.42	50206.06	51387.24
44.00	69.51	151.84	31.12	76.12	40.96	90.64	10.35
308.00	816.73	592.89	287.89	506.42	1428.18	3492.09	137.14
33.00	81.09	36.15	15.56	26.41	40.96	129.28	23.29
27.50	81.09	54.23	120.60	83.88	87.38	133.50	10.35
0.00	0.00	3.62	0.00	1.55	2.73	0.00	2.59
5.50	5.79	7.23	11.67	1.55	27.31	25.29	0.00
181.50	156.40	108.46	190.63	212.82	256.69	189.01	173.37
54521.56	153209.78	100433.49	69769.69	81922.86	296930.95	526270.58	45060.55
5.50	17.38	14.46	11.67	3.11	8.19	11.24	0.00
159.50	1424.94	3293.43	579.66	535.93	939.38	568.43	175.96
60.50	312.79	365.13	190.63	150.68	207.54	108.91	67.28
143.00	46.34	14.46	245.09	402.34	581.65	223.44	10.35
275.00	5.79	0.00	684.70	407.00	5.46	2.81	351.91
11.00	17.38	36.15	27.23	34.18	40.96	37.24	10.35
5087.51	63566.13	39401.91	9947.65	10219.97	26095.06	28399.79	4458.44
0.00	5.79	0.00	0.00	0.00	21.85	7.73	0.00
0.00	0.00	0.00	0.00	0.00	0.00	0.00	0.00
66.00	121.64	104.84	128.38	172.43	311.31	145.45	119.03
11.00	0.00	3.62	0.00	1.55	0.00	0.00	2.59
0.00	0.00	0.00	7.78	0.00	0.00	14.05	0.00

16.50	11.58	50.61	11.67	27.96	24.58	14.05	5.18
5.50	17.38	54.23	97.26	32.62	60.08	33.02	23.29
0.00	0.00	10.85	11.67	0.00	5.46	3.51	2.59
5.50	11.58	25.31	35.01	40.39	32.77	18.97	10.35
0.00	11.58	3.62	0.00	7.77	0.00	23.89	0.00
0.00	0.00	0.00	0.00	0.00	0.00	2.81	2.59
77.00	179.57	130.15	159.50	114.95	221.19	103.29	36.23
1903.00	2803.54	3698.34	3812.55	3015.19	2905.52	2616.61	2603.13
412.50	712.47	986.95	618.57	708.36	557.07	539.62	504.58
11.00	0.00	7.23	7.78	27.96	71.00	59.02	20.70
55.00	63.72	47.00	50.57	54.37	158.38	76.59	77.63
0.00	0.00	3.62	31.12	21.75	0.00	0.00	0.00
0.00	0.00	0.00	0.00	7.77	46.42	12.65	0.00
0.00	0.00	32.54	15.56	10.87	43.69	23.89	0.00
22.00	0.00	39.77	15.56	20.19	27.31	13.35	23.29
22.00	34.75	137.38	0.00	62.14	73.73	61.13	46.58
0.00	0.00	10.85	0.00	1.55	8.19	4.22	5.18
22.00	34.75	137.38	70.03	62.14	73.73	60.43	46.58
0.00	0.00	0.00	0.00	242.33	0.00	169.33	0.00
16.50	11.58	3.62	0.00	3.11	21.85	14.05	2.59
38.50	0.00	36.15	0.00	26.41	0.00	17.57	15.53
38.50	11.58	21.69	19.45	10.87	8.19	16.16	18.11
0.00	0.00	0.00	15.56	20.19	13.65	4.92	23.29
0.00	0.00	0.00	0.00	12.43	0.00	0.00	2.59
0.00	5.79	0.00	11.67	0.00	2.73	0.00	2.59
0.00	0.00	25.31	15.56	15.53	19.12	10.54	5.18
0.00	11.58	0.00	0.00	0.00	5.46	7.03	0.00
0.00	5.79	14.46	27.23	6.21	8.19	18.27	10.35
55.00	40.55	65.07	120.60	65.24	87.38	40.75	75.04
0.00	0.00	3.62	3.89	6.21	0.00	3.51	0.00
0.00	17.38	0.00	0.00	10.87	5.46	2.81	5.18
0.00	11.58	0.00	31.12	23.30	38.23	36.54	18.11
5.50	5.79	0.00	0.00	10.87	2.73	7.73	15.53
0.00	5.79	3.62	0.00	0.00	0.00	0.00	5.18
0.00	5.79	10.85	23.34	4.66	8.19	11.94	5.18
0.00	0.00	14.46	3.89	4.66	5.46	2.11	2.59
0.00	0.00	7.23	11.67	3.11	0.00	14.76	5.18
0.00	0.00	0.00	15.56	0.00	5.46	0.00	0.00
0.00	5.79	3.62	0.00	0.00	5.46	0.00	5.18
0.00	0.00	0.00	7.78	1.55	8.19	2.11	0.00
0.00	0.00	0.00	7.78	3.11	8.19	2.11	0.00
5.50	5.79	7.23	0.00	1.55	0.00	0.00	0.00
0.00	0.00	0.00	0.00	0.00	0.00	102.58	0.00
5.50	86.89	21.69	31.12	32.62	84.65	0.00	36.23
0.00	0.00	14.46	0.00	1.55	0.00	2.11	5.18
27.50	0.00	0.00	0.00	0.00	0.00	4.92	0.00
0.00	28.96	3.62	11.67	10.87	43.69	21.78	12.94
0.00	17.38	7.23	15.56	13.98	8.19	2.11	20.70

2794.00	3701.36	3539.27	1283.82	1320.41	1365.38	2101.58	5868.69
11.00	0.00	21.69	0.00	3.11	10.92	14.05	20.70
27.50	28.96	28.92	38.90	26.41	43.69	54.81	25.88
0.00	5.79	25.31	7.78	6.21	13.65	1.41	0.00
0.00	5.79	25.31	7.78	6.21	13.65	4.22	0.00
5.50	0.00	14.46	15.56	0.00	0.00	2.11	2.59
27.50	11.58	0.00	0.00	3.11	0.00	2.11	2.59
5.50	0.00	14.46	0.00	0.00	2.73	0.00	10.35
16.50	0.00	0.00	3.89	3.11	0.00	0.00	2.59
0.00	0.00	7.23	0.00	1.55	0.00	0.70	0.00
22.00	34.75	21.69	46.68	38.84	24.58	28.81	38.81
22.00	0.00	0.00	0.00	0.00	0.00	0.70	0.00
49.50	46.34	39.77	58.36	59.03	19.12	21.78	54.34
3223.00	10866.60	7805.19	5395.93	6740.30	19115.26	7405.77	3801.19
3822.50	9887.68	10755.18	5831.65	5143.38	7132.72	4821.48	2905.88
16.50	0.00	0.00	0.00	1.55	0.00	0.70	5.18
33.00	17.38	28.92	31.12	68.35	43.69	20.38	62.10
5.50	11.58	0.00	0.00	0.00	0.00	10.54	0.00
115.50	63.72	61.46	0.00	0.00	0.00	74.48	23.29
0.00	17.38	7.23	3.89	1.55	0.00	0.70	2.59
5.50	11.58	14.46	7.78	1.55	0.00	1.41	2.59
3822.50	9887.68	10755.18	5831.65	5143.38	7132.72	4821.48	2905.88
22.00	0.00	14.46	23.34	17.09	8.19	3.51	38.81
16.50	0.00	7.23	7.78	9.32	5.46	1.41	0.00
16.50	0.00	0.00	7.78	9.32	5.46	1.41	0.00
170.50	301.21	336.21	112.82	104.08	90.11	61.13	82.80
0.00	0.00	0.00	0.00	0.00	0.00	1.41	0.00
82.50	86.89	140.99	322.90	191.07	71.00	113.12	170.78
5.50	11.58	3.62	7.78	0.00	8.19	0.70	0.00
0.00	0.00	0.00	3.89	0.00	2.73	4.22	2.59
3096.50	2137.41	3568.19	8562.68	7108.46	4093.40	8114.03	4807.77
72242.57	45192.54	63309.14	87583.64	92170.79	62820.93	33812.19	46571.71
3267.00	5195.81	3806.79	5905.56	6780.69	11638.46	10854.30	3912.46
93.50	185.36	187.99	128.38	96.31	150.19	95.56	108.68
2766.50	4616.57	4598.52	3509.10	3496.75	6299.84	4496.86	2248.63
5791.51	7854.54	6952.00	9733.68	10064.63	11930.65	19633.72	9763.03
467.50	619.79	791.73	774.18	717.68	824.69	937.31	504.58
11.00	5.79	0.00	3.89	7.77	35.50	12.65	0.00
104.50	191.15	104.84	27.23	35.73	344.07	266.30	20.70
0.00	28.96	28.92	38.90	18.64	0.00	27.40	31.05
5.50	17.38	166.30	85.59	34.18	0.00	23.89	23.29
0.00	17.38	3.62	27.23	4.66	0.00	0.00	2.59
1072.50	1297.50	1218.32	1774.00	2021.00	2332.06	4662.68	732.29
242.00	324.38	177.14	225.64	380.59	322.23	388.56	212.18
253.00	167.98	144.61	420.16	396.12	270.34	350.61	227.71
159.50	0.00	119.30	287.89	239.23	144.73	0.00	163.02
3014.00	3666.61	3969.47	3738.63	3297.92	2678.87	3440.10	3555.37
39473.54	35293.28	37634.09	38242.23	44227.50	45415.12	88145.52	26398.74

55.00	23.17	25.31	42.79	34.18	27.31	11.94	36.23
1072.50	509.73	676.04	2182.49	2031.88	660.84	1869.71	732.29
1826.00	1094.77	1901.59	3123.96	3397.34	1313.49	1965.97	1754.40
25058.03	14162.49	21850.18	28162.31	31388.46	15608.97	14623.23	20646.50
979.00	880.45	1091.79	1085.41	1144.87	1196.07	1593.58	1003.99
1155.00	1007.88	911.03	1206.01	1541.00	1471.87	3739.42	724.53
2321.00	1343.84	2454.71	5866.66	4883.96	1269.80	1038.49	2416.82
5.50	46.34	39.77	0.00	7.77	24.58	47.78	0.00
93.50	220.11	57.84	81.70	178.64	223.92	650.64	108.68
15075.52	12824.44	25187.00	18319.70	11226.59	6695.80	1628.00	11874.52
11.00	28.96	122.92	11.67	9.32	5.46	0.70	20.70
38.50	5.79	14.46	11.67	23.30	10.92	4.92	7.76
55.00	150.60	148.22	38.90	45.05	38.23	25.29	20.70
55.00	81.09	28.92	97.26	97.87	24.58	75.88	67.28
16.50	34.75	50.61	11.67	6.21	19.12	16.16	7.76
11.00	11.58	14.46	46.68	49.71	24.58	40.75	25.88
77.00	115.85	126.53	93.37	57.48	87.38	69.56	56.93
0.00	5.79	3.62	3.89	7.77	10.92	12.65	7.76
11.00	11.58	18.08	3.89	20.19	0.00	7.73	7.76
434.50	196.94	350.67	754.73	753.41	136.54	1113.68	323.45
357.50	1083.18	516.97	544.65	739.43	1881.49	2369.99	419.19
165.00	173.77	202.45	190.63	229.91	188.42	108.21	137.14
16.50	5.79	43.38	7.78	15.53	27.31	26.70	12.94
1325.50	984.71	1446.07	1135.98	627.58	805.57	694.20	916.01
77.00	81.09	101.23	89.48	66.80	65.54	45.67	75.04
11.00	0.00	10.85	7.78	38.84	2.73	2.81	5.18
137.50	295.41	314.52	268.43	208.16	185.69	178.47	113.85
71.50	40.55	47.00	35.01	63.69	16.38	39.35	33.64
5.50	5.79	0.00	0.00	6.21	21.85	0.70	2.59
3630.00	556.07	582.04	7609.54	5830.00	671.76	333.75	5105.34
5.50	11.58	3.62	3.89	4.66	10.92	18.27	2.59
0.00	0.00	0.00	0.00	0.00	2.73	10.54	0.00
60.50	0.00	68.69	70.03	82.33	71.00	84.32	43.99
66.00	46.34	47.00	101.15	102.53	92.85	145.45	87.98
93.50	17.38	14.46	190.63	139.81	0.00	1.41	160.43
93.50	0.00	7.23	190.63	139.81	0.00	1.41	160.43
253.00	139.02	90.38	400.71	438.07	229.38	715.28	160.43
324.50	144.81	126.53	490.19	588.75	365.92	715.28	318.28
0.00	0.00	3.62	0.00	7.77	13.65	0.00	0.00
7133.51	3232.18	4620.21	9091.77	7782.65	3896.78	3515.98	8960.87
4218.50	29888.94	18755.59	5349.24	4186.47	7796.29	3603.11	4145.34
0.00	17.38	21.69	0.00	12.43	19.12	13.35	12.94
643.50	5056.79	2689.70	797.52	722.34	1206.99	972.45	551.16
77.00	1563.96	650.73	31.12	60.58	62.81	144.74	49.16
11.00	5.79	14.46	7.78	21.75	10.92	6.32	7.76
0.00	0.00	0.00	0.00	4.66	5.46	0.00	0.00
27.50	0.00	10.85	3.89	12.43	10.92	0.70	0.00
0.00	5.79	36.15	3.89	6.21	0.00	0.70	5.18

27.50	0.00	3.62	23.34	10.87	5.46	16.86	2.59
49.50	104.26	97.61	198.41	164.66	103.77	54.81	77.63
1848.00	0.00	1905.20	2913.88	2440.43	1463.68	456.01	1788.03
8294.01	0.00	6847.16	18459.75	5837.76	3574.55	2097.36	13528.00
0.00	0.00	3.62	3.89	0.00	0.00	0.00	0.00
11.00	5.79	7.23	3.89	4.66	0.00	4.22	2.59
60.50	127.43	137.38	0.00	82.33	95.58	114.53	54.34
242.00	225.90	242.22	0.00	276.51	172.04	153.17	181.13
5.50	0.00	3.62	38.90	24.85	2.73	14.05	10.35
451.00	127.43	303.68	556.32	598.07	314.04	200.25	302.75
5.50	11.58	0.00	0.00	0.00	10.92	0.70	0.00
0.00	11.58	21.69	3.89	9.32	5.46	7.03	2.59
0.00	0.00	10.85	3.89	7.77	8.19	13.35	5.18
0.00	0.00	54.23	7.78	24.85	0.00	1.41	0.00
11.00	46.34	14.46	7.78	10.87	10.92	18.97	2.59
5.50	5.79	21.69	11.67	1.55	0.00	1.41	0.00
16.50	81.09	61.46	23.34	4.66	0.00	21.08	2.59
203.50	156.40	206.07	392.93	388.36	185.69	127.88	134.56
27.50	0.00	119.30	27.23	18.64	5.46	18.27	5.18
5.50	23.17	3.62	0.00	3.11	0.00	1.41	2.59
16.50	11.58	0.00	3.89	3.11	8.19	2.11	10.35
11.00	11.58	7.23	3.89	4.66	0.00	1.41	2.59
0.00	5.79	32.54	0.00	3.11	16.38	3.51	0.00
60.50	139.02	65.07	97.26	114.95	51.88	108.21	62.10
170.50	330.17	220.53	252.87	236.12	365.92	223.44	276.87
49.50	63.72	14.46	54.47	60.58	62.81	33.02	46.58
11.00	0.00	10.85	15.56	27.96	8.19	18.97	7.76
0.00	0.00	0.00	0.00	3.11	2.73	0.70	0.00
5.50	17.38	47.00	3.89	13.98	2.73	28.81	0.00
16.50	11.58	36.15	11.67	21.75	27.31	76.59	12.94
2783.00	1320.67	1822.05	4014.85	3391.12	1679.41	1383.49	2652.29
5.50	17.38	0.00	0.00	1.55	5.46	7.73	0.00
913.00	642.96	1344.85	2964.45	2277.32	412.34	399.80	2067.50
11.00	0.00	28.92	0.00	3.11	0.00	16.16	2.59
49.50	40.55	10.85	46.68	34.18	71.00	120.85	28.46
159.50	162.19	140.99	260.65	259.42	278.54	138.42	155.26
5.50	11.58	3.62	3.89	6.21	0.00	2.81	2.59
11.00	0.00	7.23	11.67	7.77	0.00	1.41	23.29
55.00	17.38	32.54	7.78	12.43	21.85	14.76	5.18
38.50	23.17	28.92	50.57	38.84	81.92	88.53	25.88
82.50	40.55	72.30	171.18	85.44	49.15	31.62	54.34
16.50	34.75	36.15	0.00	0.00	19.12	0.70	2.59
913.00	712.47	1001.41	976.48	1126.23	1228.84	652.05	706.42
0.00	86.89	25.31	15.56	6.21	32.77	8.43	12.94
121.00	63.72	101.23	175.07	183.30	136.54	151.77	134.56
44.00	23.17	18.08	7.78	10.87	10.92	11.94	10.35
137.50	173.77	249.45	252.87	231.46	324.96	493.25	183.72
330.00	422.85	571.20	560.21	264.08	103.77	45.67	315.69

1424.50	1627.67	1753.37	2236.96	2247.80	1348.99	1615.36	1591.38
55.00	150.60	292.83	124.49	108.74	43.69	21.08	41.40
11.00	17.38	7.23	31.12	10.87	13.65	19.67	28.46
198.00	57.92	68.69	132.27	229.91	125.61	109.61	173.37
1023.00	758.81	972.49	1400.53	1106.04	985.80	682.96	1562.91
11.00	5.79	3.62	3.89	4.66	10.92	13.35	0.00
11.00	0.00	28.92	46.68	24.85	8.19	9.13	12.94
121.00	104.26	180.76	147.83	108.74	109.23	108.21	106.09
264.00	2062.11	3156.06	412.38	282.72	251.23	88.53	212.18
121.00	69.51	79.53	81.70	73.01	117.42	181.98	36.23
0.00	5.79	7.23	0.00	9.32	8.19	0.00	7.76
22.00	115.85	122.92	50.57	35.73	49.15	16.86	18.11
33.00	40.55	54.23	105.04	90.10	114.69	87.13	64.69
77.00	110.06	159.07	260.65	201.94	114.69	57.62	139.73
11.00	11.58	14.46	11.67	3.11	21.85	1.41	5.18
11.00	5.79	0.00	3.89	4.66	5.46	14.05	5.18
0.00	0.00	18.08	0.00	3.11	0.00	0.70	0.00
0.00	5.79	3.62	3.89	26.41	5.46	1.41	2.59
0.00	11.58	7.23	11.67	27.96	8.19	1.41	23.29
11.00	115.85	72.30	15.56	6.21	35.50	21.78	12.94
22.00	11.58	18.08	3.89	15.53	13.65	7.73	10.35
44.00	40.55	75.92	711.94	518.84	84.65	103.99	150.08
2156.00	938.37	1449.69	6555.25	6943.80	1960.68	4589.61	3917.63
55.00	0.00	10.85	93.37	74.56	30.04	49.89	75.04
176.00	359.13	332.60	482.40	567.00	679.96	1639.25	207.01
16.50	52.13	21.69	280.11	312.24	327.69	441.96	20.70
0.00	11.58	28.92	15.56	17.09	10.92	38.64	15.53
0.00	0.00	0.00	38.90	55.92	24.58	29.51	0.00
82.50	191.15	101.23	105.04	136.70	155.65	378.72	72.45
951.50	1268.54	1142.40	1143.77	1655.95	1499.18	948.56	1278.28
11.00	5.79	0.00	7.78	6.21	2.73	2.81	0.00
27.50	23.17	18.08	0.00	21.75	8.19	19.67	7.76
71060.07	57177.08	94797.41	70613.90	63491.50	35406.92	42283.15	49640.61
583.00	1251.16	1362.93	1645.62	1349.92	1237.03	2814.75	1203.24
22.00	81.09	83.15	38.90	0.00	35.50	19.67	15.53
0.00	0.00	0.00	0.00	29.52	46.42	11.94	0.00
0.00	0.00	21.69	0.00	0.00	21.85	40.05	0.00
0.00	0.00	0.00	0.00	26.41	0.00	0.00	0.00
71.50	0.00	0.00	85.59	116.51	0.00	0.00	0.00
0.00	0.00	0.00	0.00	38.84	0.00	27.40	0.00
0.00	0.00	567.58	0.00	0.00	144.73	16.16	0.00
682.00	3909.89	1746.13	307.34	608.94	4036.05	2071.37	126.79
0.00	0.00	0.00	38.90	31.07	49.15	18.27	0.00
137.50	0.00	122.92	182.85	243.89	79.19	13.35	100.92
0.00	0.00	43.38	0.00	20.19	0.00	43.56	0.00
0.00	0.00	0.00	0.00	177.09	0.00	0.00	0.00
0.00	561.87	462.74	0.00	0.00	43.69	2.81	0.00
0.00	0.00	0.00	0.00	62.14	0.00	0.00	0.00

0.00	341.75	108.46	0.00	0.00	32.77	36.54	0.00
0.00	0.00	0.00	0.00	0.00	0.00	41.46	0.00
0.00	0.00	0.00	0.00	338.65	0.00	0.00	0.00
0.00	0.00	0.00	0.00	24.85	0.00	0.00	0.00
0.00	0.00	216.91	0.00	0.00	84.65	66.05	62.10
533.50	0.00	0.00	0.00	0.00	57.35	0.00	0.00
0.00	0.00	0.00	280.11	312.24	327.69	441.96	0.00
0.00	1106.35	921.87	1104.86	1135.55	2184.60	1122.11	815.10
0.00	0.00	0.00	0.00	7.77	13.65	4.22	0.00
583.00	2073.69	2418.56	953.14	939.82	3547.25	63.94	494.23
649.00	0.00	0.00	0.00	0.00	0.00	0.00	0.00
0.00	0.00	47.00	0.00	0.00	0.00	14.76	0.00
0.00	0.00	50.61	0.00	0.00	0.00	24.59	0.00
489.50	237.49	412.13	373.47	366.61	319.50	119.45	1151.48
0.00	0.00	0.00	0.00	24.85	0.00	0.00	0.00
0.00	191.15	122.92	0.00	0.00	43.69	40.05	0.00

cow3_D3	cow3_D10	cow3_D21	cow4_D0	cow4_D3	cow4_D10	cow4_D21	cow5_D0
0.00	2.08	0.00	4.18	13.39	7.59	16.65	6.80
0.00	2.08	2.05	6.96	0.89	0.99	14.99	1.13
95.46	132.94	116.70	104.38	215.14	179.76	273.10	119.62
112399.91	92363.54	99884.48	129865.61	91096.98	72255.91	52205.18	111216.09
60115.62	61697.56	49264.55	70206.95	59458.66	47273.91	22340.82	54627.59
25558.77	11451.71	9823.43	26222.53	14417.66	7821.26	5433.67	26689.07
1969.32	1414.59	1537.60	6617.91	3036.00	1207.18	1901.70	3270.44
1534.45	926.44	1056.46	889.35	759.67	581.82	927.54	641.16
24006.65	53696.11	41586.80	24023.52	36987.25	57497.99	48495.03	17027.40
19247.75	28848.42	29881.81	14045.84	33487.06	44557.43	48067.07	12741.07
7025.22	8601.76	8339.06	4022.24	8254.60	7938.68	46781.51	5799.95
99.00	56.08	98.28	151.70	128.55	46.84	174.85	117.35
604.59	2160.31	1918.41	913.01	2316.50	7033.63	3163.95	960.90
0.00	4.15	6.14	2.78	3.57	11.54	28.31	2.27
10.61	0.00	0.00	13.92	0.00	0.00	0.00	5.67
2061.25	7617.16	5691.77	4052.86	0.00	5299.05	9142.15	2994.93
63.64	39.47	22.52	0.00	50.88	13.85	9.99	47.05
0.00	10.39	2.05	16.70	39.28	4.29	41.63	1.70
0.00	2.08	0.00	4.18	15.18	1.65	16.65	0.00
2923.93	11175.44	10916.74	4087.66	5124.87	12554.99	9711.66	2842.44
17.68	122.56	83.94	9.74	35.71	176.79	171.52	7.37
491.45	535.92	591.70	318.72	477.58	636.57	374.68	431.41
215.67	392.59	509.80	338.20	667.72	615.79	854.27	342.97
0.00	6.23	14.33	6.96	4.46	2.97	38.30	3.40
31.82	31.16	81.90	157.27	84.80	118.08	76.60	54.99
14.14	4.15	6.14	15.31	6.25	6.27	14.99	13.61
3.54	4.15	20.47	5.57	0.89	7.59	13.32	3.97
0.00	0.00	0.00	11.13	5.36	0.99	8.33	22.11
0.00	14.54	8.19	44.54	85.70	9.89	479.59	21.54
3.54	2.08	2.05	25.05	14.28	1.65	1.67	24.94
60.11	222.26	43.00	27.84	87.48	123.69	303.07	23.81
3.54	8.31	0.00	25.05	8.93	2.97	1.67	11.34
0.00	0.00	6.14	1.39	0.89	0.00	14.99	0.57
10.61	10.39	22.52	43.15	66.06	24.41	56.62	22.68
0.00	8.31	2.05	2.78	5.36	5.94	39.97	4.54
137.89	124.63	124.89	73.76	1.79	102.25	464.60	67.46
0.00	2.08	2.05	1.39	0.89	2.64	3.33	0.57
0.00	2.08	2.05	0.00	0.00	2.97	0.00	0.00
14.14	14.54	8.19	1.39	3.57	16.16	11.66	2.27
3.54	6.23	12.28	1.39	6.25	3.63	6.66	1.13
3.54	0.00	6.14	1.39	2.68	2.31	0.00	1.13
0.00	0.00	2.05	0.00	0.00	0.00	1.67	0.00
7.07	0.00	0.00	0.00	0.00	1.32	5.00	5.10
0.00	0.00	0.00	1.39	1.79	0.66	6.66	2.83
3.54	2.08	4.09	1.39	0.89	1.98	11.66	1.13
0.00	2.08	0.00	0.00	1.79	1.98	0.00	2.83

0.00	2.08	0.00	2.78	0.00	0.66	1.67	0.57
3.54	4.15	10.24	0.00	0.00	1.65	3.33	0.57
7.07	0.00	2.05	1.39	0.89	0.00	11.66	1.70
0.00	2.08	0.00	0.00	2.68	1.32	0.00	4.54
7.07	2.08	4.09	126.65	6.25	0.99	0.00	92.97
5543.80	4636.35	3351.59	4739.01	8470.63	1677.19	73688.41	2450.14
0.00	2.08	2.05	1.39	0.00	1.98	1.67	4.54
427.81	423.75	1142.45	1162.14	858.76	712.43	1605.29	960.33
678.83	818.42	1357.43	1590.80	1123.88	1089.10	2646.06	1370.20
77.78	87.24	47.09	126.65	136.58	184.05	106.58	145.69
21.21	6.23	6.14	23.66	16.07	9.89	26.64	60.66
10.61	8.31	8.19	29.23	19.64	15.50	8.33	33.45
53.03	39.47	53.23	36.19	75.88	60.03	143.21	31.75
229.81	112.17	139.22	890.74	378.50	58.71	188.17	809.53
10.61	22.85	12.28	2.78	6.25	10.22	11.66	3.97
0.00	2.08	4.09	0.00	2.68	1.65	16.65	1.13
3.54	4.15	14.33	13.92	7.14	2.31	21.65	17.57
272.24	89.32	67.56	821.15	355.29	136.22	149.87	593.54
0.00	8.31	16.38	1.39	14.28	14.84	68.27	1.70
0.00	56.08	57.33	4.18	23.21	41.56	159.86	3.97
0.00	2.08	2.05	6.96	11.60	10.88	31.64	8.50
21.21	8.31	8.19	90.47	11.60	9.57	11.66	19.27
42.43	43.62	47.09	30.62	64.27	28.04	23.31	24.94
0.00	0.00	0.00	1.39	0.89	2.31	14.99	1.13
14.14	20.77	36.85	5.57	1.79	15.50	126.56	6.24
0.00	18.69	18.43	6.96	21.42	8.58	24.98	6.24
1230.38	2874.87	2815.18	1125.95	2290.61	3943.12	7112.23	835.04
1311.70	1726.17	2010.55	5355.57	7700.25	5083.67	2194.78	3340.74
28.28	0.00	0.00	112.73	837.33	405.36	9092.19	69.73
162.64	243.03	171.98	377.17	489.19	252.98	616.14	143.99
7.07	0.00	8.19	45.93	15.18	2.97	3.33	11.34
88.39	72.70	83.94	175.36	74.99	40.90	59.95	175.74
3.54	39.47	45.04	26.44	34.81	66.63	1205.63	14.74
325.27	440.37	620.36	725.12	499.01	555.10	1019.13	345.24
28.28	60.24	100.32	1.39	55.35	70.58	454.61	3.40
974387.13	728127.55	627534.38	673270.83	700894.81	396584.59	254636.40	864309.49
2987.57	3610.21	3435.54	1505.91	3087.78	3877.81	3816.72	2641.75
21.21	4.15	4.09	20.88	23.21	8.58	38.30	13.04
3.54	20.77	18.43	33.40	39.28	38.59	949.19	29.48
102.53	101.78	126.94	192.07	99.98	148.09	226.47	99.77
17246.60	9158.46	9196.92	15157.88	14156.99	8311.06	6705.91	17493.39
707.12	2669.23	2076.06	342.38	931.06	2764.97	1483.73	422.34
123.75	172.41	243.64	254.70	284.76	181.74	3828.38	172.34
10.61	12.46	18.43	6.96	31.24	39.91	369.68	10.20
24.75	29.08	61.42	20.88	113.37	74.21	870.92	21.54
127.28	336.51	362.39	274.18	505.26	803.80	2334.66	172.34
49.50	110.09	159.70	122.48	205.32	295.20	1355.50	72.00
7.07	2.08	0.00	2.78	15.18	12.20	16.65	6.80

74.25	456.99	550.75	141.96	324.94	1071.62	920.88	142.86
1050.07	1346.04	1682.96	1546.27	2247.77	1860.24	4269.67	1494.91
282.85	317.81	311.20	672.23	411.52	365.12	717.72	378.12
3.54	2.08	2.05	2.78	3.57	13.85	98.25	1.13
3.54	20.77	36.85	47.32	37.49	31.00	219.81	10.20
3783.08	4056.81	4158.27	6212.91	4381.27	5325.11	6246.30	2904.79
576.30	940.98	1119.93	524.70	657.90	1140.22	1773.48	442.18
1081.89	1784.33	1463.89	1255.39	1608.61	2321.02	2116.52	691.05
17.68	4.15	22.52	6.96	19.64	6.93	24.98	38.55
162.64	463.22	528.23	336.81	897.14	461.76	1307.21	177.44
4575.05	5614.72	7292.84	10097.36	13250.92	12754.87	10142.96	6595.31
3.54	0.00	6.14	6.96	3.57	12.86	28.31	2.27
3.54	2.08	6.14	18.09	16.07	3.30	16.65	22.68
5391.77	1921.43	2065.83	12235.14	7138.75	2802.57	5029.02	8437.17
364.17	589.93	870.15	325.68	623.09	910.33	1195.64	359.98
3.54	0.00	0.00	0.00	17.85	0.33	0.00	1.70
339.42	309.51	292.78	1066.10	412.42	170.52	329.72	487.53
3.54	4.15	4.09	0.00	3.57	3.63	6.66	0.00
159.10	361.44	477.04	540.01	655.23	778.73	774.34	285.15
0.00	4.15	4.09	1.39	0.89	1.32	26.64	1.13
183.85	297.04	300.97	471.81	342.79	354.57	136.55	136.06
0.00	2.08	4.09	4.18	8.03	2.64	11.66	3.97
77.78	39.47	79.85	101.60	97.30	88.72	211.49	153.06
21.21	8.31	20.47	20.88	57.13	25.07	48.29	35.71
21.21	72.70	88.04	62.63	152.65	142.82	274.76	85.60
20870.58	16879.48	18725.52	36214.12	50252.46	28843.33	21698.04	26088.16
42052.30	50574.05	62634.07	63994.05	55887.05	70868.65	62691.18	46947.23
33492.64	31768.99	32688.80	89156.04	54065.09	39260.35	24881.97	67919.69
7.07	31.16	67.56	12.53	55.35	11.87	44.96	9.64
183.85	1055.23	1183.40	240.78	649.87	2763.65	1761.82	224.49
10.61	83.09	63.47	15.31	48.20	107.52	123.23	26.08
24.75	47.78	59.37	27.84	51.78	97.63	329.72	34.58
0.00	2.08	0.00	1.39	1.79	0.99	3.33	0.57
7.07	18.69	12.28	0.00	12.50	29.03	49.96	1.70
141.42	228.49	188.36	96.03	133.90	205.15	121.56	105.44
71001.70	298600.22	339291.11	64888.96	119289.56	508434.65	361331.47	46045.86
7.07	14.54	16.38	0.00	23.21	18.80	394.66	1.13
84.85	411.29	704.31	122.48	406.17	171.51	4183.08	105.44
21.21	37.39	90.09	27.84	48.20	19.46	890.90	18.71
35.36	62.32	18.43	19.48	331.18	47.50	1049.10	11.90
243.96	0.00	0.00	388.31	304.40	0.33	0.00	362.25
7.07	14.54	34.81	1.39	37.49	10.22	109.91	3.97
4500.80	24382.40	23147.91	10070.92	9328.49	17836.89	19103.60	6331.70
0.00	0.00	10.24	0.00	1.79	5.94	9.99	1.13
0.00	2.08	0.00	0.00	1.79	6.93	1.67	0.57
53.03	58.16	90.09	65.41	113.37	67.62	179.85	93.54
0.00	0.00	2.05	0.00	0.00	0.00	1.67	0.00
0.00	0.00	0.00	0.00	0.89	4.62	1.67	0.57

3.54	24.93	22.52	25.05	12.50	16.16	28.31	6.24
24.75	27.00	24.57	93.25	45.53	55.74	56.62	28.91
7.07	2.08	10.24	2.78	5.36	3.63	5.00	1.13
17.68	14.54	2.05	47.32	19.64	15.83	41.63	19.27
7.07	0.00	0.00	2.78	1.79	0.00	1.67	9.07
0.00	0.00	4.09	0.00	4.46	3.63	13.32	1.13
21.21	85.17	61.42	29.23	62.49	63.33	722.71	10.20
2089.53	2197.70	3754.93	2232.42	2107.61	2112.89	4909.12	1446.73
392.45	436.22	651.07	643.00	326.72	305.75	664.43	171.77
0.00	37.39	40.95	13.92	58.92	38.92	296.41	18.14
28.28	91.40	61.42	108.56	57.13	121.38	38.30	32.31
0.00	0.00	2.05	27.84	0.89	1.65	1.67	13.61
0.00	31.16	0.00	0.00	10.71	12.86	0.00	13.61
0.00	39.47	0.00	0.00	0.00	7.92	0.00	11.34
17.68	12.46	12.28	12.53	7.14	2.31	8.33	11.34
45.96	39.47	55.28	93.25	47.31	0.00	0.00	30.61
3.54	0.00	2.05	5.57	0.00	12.53	1.67	0.00
45.96	39.47	55.28	93.25	47.31	46.51	136.55	30.61
152.03	0.00	0.00	183.71	0.00	85.10	0.00	56.12
0.00	14.54	12.28	0.00	6.25	4.62	36.64	1.70
24.75	20.77	0.00	32.01	23.21	14.18	0.00	32.88
3.54	2.08	12.28	0.00	0.89	0.00	0.00	0.57
0.00	4.15	10.24	6.96	12.50	4.29	28.31	0.57
0.00	0.00	6.14	9.74	0.00	9.89	6.66	5.67
0.00	2.08	0.00	23.66	24.10	0.00	0.00	0.00
7.07	18.69	10.24	8.35	2.68	1.65	16.65	2.27
0.00	0.00	0.00	0.00	5.36	0.66	23.31	0.00
7.07	18.69	14.33	9.74	21.42	28.04	56.62	7.37
74.25	51.93	53.23	68.20	48.20	44.53	139.88	62.36
0.00	0.00	2.05	2.78	1.79	6.27	3.33	0.00
3.54	4.15	10.24	0.00	0.89	3.63	5.00	2.83
10.61	33.24	24.57	4.18	8.03	3.30	38.30	13.61
14.14	4.15	8.19	8.35	7.14	3.30	13.32	10.77
7.07	18.69	0.00	12.53	14.28	5.94	11.66	4.54
7.07	14.54	6.14	11.13	22.32	21.77	46.63	6.80
3.54	6.23	2.05	1.39	0.89	1.65	1.67	0.57
0.00	6.23	12.28	1.39	10.71	15.17	24.98	3.40
0.00	4.15	24.57	16.70	16.07	38.26	9.99	0.00
0.00	10.39	12.28	4.18	14.28	13.85	0.00	4.54
0.00	0.00	2.05	1.39	9.82	9.89	24.98	2.27
0.00	0.00	2.05	1.39	9.82	10.22	24.98	2.27
0.00	4.15	0.00	5.57	6.25	5.94	11.66	2.27
0.00	87.24	69.61	0.00	67.84	78.83	106.58	0.00
10.61	0.00	0.00	36.19	0.00	0.00	0.00	14.74
0.00	0.00	0.00	5.57	0.00	0.00	1.67	0.00
0.00	0.00	0.00	22.27	25.00	15.50	6.66	2.27
28.28	16.62	34.81	4.18	33.92	53.43	78.27	8.50
24.75	8.31	2.05	6.96	8.93	5.61	9.99	11.90

5508.45	9114.84	6113.54	4194.83	4889.20	5141.06	3656.86	2467.71
7.07	4.15	12.28	15.31	20.53	14.18	3.33	1.70
10.61	24.93	30.71	40.36	0.00	0.00	0.00	15.31
3.54	2.08	0.00	2.78	7.14	3.63	8.33	0.57
3.54	2.08	0.00	2.78	7.14	3.63	8.33	0.57
0.00	4.15	2.05	8.35	11.60	0.66	38.30	1.13
3.54	4.15	0.00	2.78	5.36	3.30	8.33	6.80
0.00	2.08	4.09	2.78	0.00	1.65	0.00	2.27
3.54	4.15	0.00	0.00	0.00	1.98	0.00	1.13
0.00	2.08	0.00	2.78	2.68	0.66	14.99	0.00
14.14	27.00	36.85	59.85	35.71	31.66	59.95	28.34
0.00	0.00	10.24	0.00	0.89	0.33	5.00	0.00
24.75	49.85	38.90	25.05	15.18	17.15	36.64	11.34
3157.28	9690.23	7237.56	8406.35	7649.36	8081.83	27324.88	4078.28
2436.02	4079.66	6629.48	2610.98	4608.01	3150.21	19808.00	2798.78
10.61	2.08	6.14	2.78	6.25	0.66	0.00	6.80
56.57	56.08	28.66	0.00	0.00	40.24	24.98	56.12
0.00	0.00	0.00	2.78	0.00	0.33	1.67	4.54
7.07	12.46	0.00	235.21	85.70	31.33	26.64	195.01
0.00	0.00	0.00	0.00	0.89	0.00	1.67	2.83
7.07	0.00	4.09	0.00	2.68	0.66	5.00	2.27
2436.02	4079.66	6629.48	2610.98	4608.01	3150.21	19808.00	2798.78
21.21	22.85	2.05	19.48	8.03	8.58	1.67	11.90
0.00	2.08	2.05	5.57	0.00	3.30	23.31	2.83
0.00	2.08	2.05	5.57	0.00	0.00	23.31	2.83
99.00	74.78	57.33	97.42	158.90	64.65	274.76	159.87
0.00	2.08	0.00	1.39	1.79	1.32	11.66	0.00
63.64	81.01	92.13	137.79	61.59	75.53	146.54	97.51
0.00	0.00	0.00	0.00	1.79	0.33	1.67	0.57
0.00	0.00	0.00	4.18	2.68	1.98	59.95	1.70
3305.78	3092.98	6344.89	7714.64	5442.66	4989.34	13023.82	4543.13
46153.58	32612.34	46330.62	82503.33	105015.63	46089.49	79518.40	99119.58
2789.58	7700.25	10609.63	8066.76	14311.43	14454.15	26249.13	5936.57
123.75	126.71	104.42	77.94	108.91	131.27	298.08	45.92
2047.11	4287.38	6545.54	10574.74	8970.53	6802.09	26285.77	2770.44
6668.12	13599.55	16522.52	17000.59	13447.31	20590.64	35178.13	7587.39
282.85	635.63	560.99	544.19	482.05	819.30	1020.79	285.15
0.00	12.46	8.19	5.57	7.14	8.25	104.91	1.13
38.89	172.41	151.51	8.35	36.60	80.81	309.73	15.31
10.61	0.00	0.00	2.78	10.71	12.86	0.00	3.40
10.61	0.00	0.00	11.13	20.53	7.59	0.00	15.87
0.00	4.15	4.09	1.39	7.14	4.62	0.00	2.83
1021.79	1821.72	3195.99	1746.68	3375.22	4670.73	12724.08	1417.25
176.78	332.36	331.68	396.66	747.17	915.28	526.21	219.39
286.38	313.66	391.05	329.85	677.54	673.51	557.85	336.74
205.06	201.49	235.45	190.67	343.68	259.25	239.79	202.95
2771.90	2353.49	1854.94	2786.34	2035.31	1335.81	849.27	2819.19
32859.76	53681.57	57024.20	68251.50	61415.41	95369.10	76003.09	47711.41

35.36	4.15	12.28	16.70	15.18	3.96	24.98	20.41
774.29	700.02	866.05	1729.98	2289.72	1500.07	3102.34	934.25
1389.49	889.05	1422.94	1611.68	1261.36	496.72	1798.46	1251.71
16755.16	12423.85	14051.31	38897.47	22867.75	17910.44	16442.55	28481.61
1067.75	1125.85	1156.78	1653.43	1100.67	1023.46	965.84	835.61
689.44	1329.42	1611.30	2789.13	2322.75	3364.60	3075.69	1469.97
1488.48	648.09	972.52	872.65	905.18	201.86	1064.09	1935.96
0.00	8.31	34.81	0.00	11.60	12.86	44.96	1.70
81.32	228.49	272.30	299.23	599.88	628.66	419.64	243.77
7293.92	2654.69	2512.16	19583.74	6355.87	1931.82	4792.55	11980.29
3.54	2.08	2.05	11.13	1.79	1.98	8.33	3.97
17.68	8.31	10.24	11.13	19.64	12.86	29.97	6.80
10.61	33.24	40.95	2.78	19.64	12.86	93.25	5.67
35.36	20.77	28.66	34.79	39.28	28.04	73.27	71.43
7.07	0.00	4.09	18.09	7.14	7.26	63.28	7.94
31.82	12.46	0.00	15.31	31.24	37.60	48.29	21.54
24.75	41.54	0.00	83.51	93.73	63.99	126.56	72.56
3.54	4.15	0.00	16.70	15.18	24.74	33.30	4.54
3.54	8.31	2.05	32.01	11.60	6.60	6.66	10.77
293.45	122.56	603.98	211.55	408.85	313.67	726.04	236.96
530.34	1869.50	1801.71	739.04	1564.87	2808.83	1685.22	552.16
74.25	74.78	122.84	146.14	218.71	212.41	1015.79	130.95
7.07	4.15	12.28	6.96	13.39	17.48	148.21	3.40
615.19	614.86	389.01	590.11	211.56	208.78	196.50	284.02
38.89	29.08	36.85	47.32	23.21	17.15	9.99	22.11
17.68	6.23	4.09	9.74	35.71	26.06	41.63	13.61
148.49	85.17	110.56	414.75	201.75	203.84	301.41	174.61
38.89	35.31	32.76	36.19	84.80	93.67	183.18	12.47
3.54	6.23	0.00	0.00	0.89	1.32	11.66	1.13
4345.24	319.89	2610.44	3670.12	4931.16	4011.07	769.34	4374.77
3.54	12.46	10.24	5.57	3.57	19.46	29.97	3.97
0.00	0.00	0.00	0.00	8.93	16.49	6.66	1.13
56.57	58.16	43.00	96.03	79.45	117.09	83.26	81.07
113.14	101.78	155.60	66.81	182.11	163.60	516.22	53.29
38.89	6.23	2.05	83.51	81.23	0.00	41.63	95.81
38.89	0.00	2.05	83.51	81.23	4.95	41.63	95.81
212.14	297.04	298.92	570.63	1104.24	829.19	539.54	462.02
473.77	398.83	485.23	510.78	916.78	951.56	2497.86	315.20
3.54	2.08	2.05	1.39	8.93	2.31	5.00	2.83
5529.66	3772.23	2849.98	5702.12	4399.12	3910.14	975.83	8330.02
3701.76	7149.79	9385.28	6293.63	4409.83	5651.31	27229.96	2901.39
3.54	20.77	14.33	151.70	114.26	80.15	817.63	82.77
643.48	1709.55	2112.92	1085.59	768.60	1784.05	4592.72	540.26
49.50	213.95	249.78	22.27	22.32	145.13	198.16	14.74
3.54	4.15	2.05	29.23	13.39	10.88	1.67	18.14
7.07	0.00	0.00	1.39	13.39	6.93	3.33	15.87
3.54	4.15	2.05	50.10	14.28	5.28	1.67	27.78
0.00	0.00	0.00	5.57	1.79	0.66	1.67	10.20

10.61	2.08	2.05	44.54	26.78	12.20	18.32	39.68
56.57	49.85	57.33	247.74	141.94	84.11	126.56	118.48
1060.68	826.73	1334.91	668.05	645.41	516.84	1009.13	975.63
5490.77	1611.92	1347.19	10068.14	3324.34	996.42	3482.01	5761.97
0.00	2.08	0.00	12.53	2.68	0.00	1.67	0.00
0.00	2.08	26.62	12.53	2.68	11.54	54.95	3.40
49.50	132.94	71.66	77.94	89.27	111.15	174.85	42.52
190.92	56.08	135.13	204.59	258.88	212.74	274.76	267.58
0.00	6.23	16.38	4.18	18.75	13.85	24.98	0.00
332.35	108.02	124.89	1057.75	703.43	129.29	253.12	513.04
3.54	4.15	2.05	0.00	1.79	0.99	1.67	0.00
0.00	8.31	4.09	16.70	16.07	11.21	46.63	2.27
0.00	8.31	22.52	1.39	5.36	24.08	111.57	3.40
0.00	6.23	4.09	55.67	4.46	1.98	74.94	11.34
0.00	10.39	24.57	1.39	8.93	13.19	109.91	0.00
0.00	4.15	2.05	0.00	6.25	0.33	44.96	0.00
7.07	10.39	38.90	1.39	27.67	8.91	146.54	3.40
84.85	58.16	106.46	119.69	127.65	109.83	228.14	189.34
3.54	2.08	22.52	47.32	15.18	4.29	134.88	15.31
3.54	2.08	4.09	2.78	2.68	2.31	1.67	0.00
7.07	0.00	2.05	4.18	7.14	0.66	1.67	4.54
0.00	0.00	0.00	5.57	7.14	3.30	5.00	6.24
0.00	0.00	0.00	4.18	2.68	0.00	3.33	1.13
56.57	83.09	106.46	139.18	274.05	238.47	116.57	103.74
190.92	176.56	192.46	180.93	236.56	241.44	419.64	183.68
24.75	29.08	32.76	62.63	84.80	48.16	81.60	56.12
3.54	8.31	6.14	6.96	19.64	12.20	41.63	11.34
0.00	0.00	0.00	0.00	2.68	1.32	13.32	0.00
10.61	4.15	16.38	2.78	16.07	4.95	188.17	1.13
3.54	14.54	14.33	33.40	19.64	25.40	124.89	5.67
2485.52	1503.91	1332.86	5091.13	2249.55	1895.53	2104.86	2296.51
0.00	14.54	16.38	5.57	3.57	24.08	33.30	0.00
2294.60	301.20	808.72	719.55	1941.58	835.46	1643.59	1246.05
3.54	2.08	6.14	0.00	6.25	9.89	16.65	3.97
24.75	66.47	45.04	54.28	88.38	128.30	131.55	39.12
127.28	135.02	98.28	61.24	52.67	60.36	74.94	66.33
3.54	0.00	2.05	0.00	10.71	0.66	9.99	0.00
3.54	2.08	8.19	12.53	4.46	0.66	0.00	7.37
3.54	0.00	4.09	11.13	10.71	11.21	18.32	6.24
21.21	54.01	26.62	58.45	61.59	119.73	34.97	38.55
42.43	47.78	22.52	29.23	45.53	28.04	38.30	32.88
3.54	4.15	10.24	0.00	0.00	2.31	18.32	0.00
622.26	434.14	540.51	732.08	620.41	739.48	1144.02	781.75
17.68	60.24	10.24	6.96	9.82	18.14	39.97	9.64
159.10	199.41	163.79	125.26	130.33	180.42	91.59	96.37
17.68	6.23	14.33	11.13	8.03	15.50	44.96	5.10
162.64	243.03	331.68	278.36	399.92	389.53	391.33	121.32
240.42	58.16	128.99	442.59	170.50	48.49	546.20	213.72

1170.28	1076.00	1410.66	1259.56	1370.26	931.11	1888.38	1527.23
21.21	143.33	57.33	64.02	66.06	48.16	64.94	50.45
14.14	33.24	14.33	8.35	10.71	16.16	31.64	15.87
282.85	118.40	57.33	217.12	144.61	140.18	48.29	150.80
1393.02	1015.76	769.82	1749.47	1114.96	1003.67	1183.98	948.99
0.00	2.08	18.43	2.78	17.85	5.28	24.98	0.57
17.68	2.08	6.14	13.92	5.36	2.97	6.66	15.87
63.64	43.62	77.80	25.05	54.45	30.67	98.25	40.82
148.49	124.63	122.84	108.56	62.49	27.38	168.19	87.87
91.93	159.95	169.93	57.06	108.01	230.55	261.44	83.33
0.00	2.08	0.00	12.53	6.25	0.00	8.33	6.80
28.28	6.23	2.05	34.79	25.89	5.61	26.64	23.24
109.60	108.02	112.61	123.87	158.90	170.19	98.25	66.89
155.57	87.24	53.23	203.20	120.51	47.83	63.28	194.45
0.00	2.08	2.05	2.78	0.89	1.65	9.99	1.70
7.07	6.23	12.28	1.39	9.82	9.24	16.65	3.97
0.00	0.00	0.00	0.00	0.00	0.33	1.67	0.00
3.54	4.15	0.00	4.18	0.89	0.66	1.67	0.00
3.54	2.08	4.09	4.18	1.79	0.99	0.00	6.24
0.00	6.23	22.52	2.78	2.68	8.25	101.58	5.10
10.61	12.46	12.28	26.44	13.39	19.79	36.64	11.34
120.21	51.93	67.56	271.40	248.16	101.26	134.88	277.21
1944.57	1412.51	1910.22	10648.51	4056.33	2737.59	4491.14	6492.70
24.75	14.54	24.57	79.33	40.17	37.93	83.26	69.73
286.38	483.99	956.14	615.17	762.35	1123.07	2344.65	289.12
31.82	47.78	106.46	37.58	98.19	177.12	136.55	5.67
10.61	10.39	28.66	11.13	28.57	25.07	58.28	4.54
0.00	12.46	8.19	11.13	16.96	11.54	8.33	0.57
106.07	201.49	229.31	115.52	315.12	360.83	467.93	103.18
1134.92	965.91	976.61	1330.54	2044.23	1278.75	2176.46	1178.02
0.00	0.00	0.00	2.78	2.68	6.60	0.00	1.70
3.54	4.15	18.43	22.27	27.67	42.88	109.91	14.74
50965.52	25975.62	47917.36	39356.76	57833.09	26384.11	85506.59	56189.40
898.04	1466.52	2094.49	1394.56	829.30	1605.61	2061.56	1127.56
0.00	27.00	30.71	34.79	42.85	24.08	0.00	24.94
0.00	0.00	0.00	0.00	14.28	4.95	68.27	11.90
0.00	22.85	0.00	0.00	14.28	12.86	0.00	15.31
0.00	0.00	0.00	0.00	6.25	3.63	0.00	7.37
0.00	0.00	0.00	75.16	32.14	0.00	0.00	0.00
0.00	33.24	0.00	25.05	23.21	48.49	0.00	24.38
0.00	0.00	137.18	0.00	0.00	0.00	0.00	0.00
420.74	2206.01	2327.89	0.00	668.62	1083.82	0.00	112.25
0.00	6.23	0.00	33.40	34.81	15.17	19.98	0.00
0.00	0.00	26.62	251.91	43.74	14.84	156.53	152.50
0.00	0.00	28.66	0.00	15.18	31.66	113.24	7.94
0.00	0.00	0.00	0.00	122.30	0.00	0.00	0.00
0.00	0.00	22.52	0.00	0.00	0.00	0.00	0.00
63.64	0.00	0.00	44.54	50.88	37.27	0.00	83.33

0.00	0.00	0.00	0.00	0.00	5.28	0.00	0.00
0.00	20.77	0.00	0.00	0.00	23.42	28.31	0.00
0.00	0.00	0.00	0.00	156.22	0.00	587.83	0.00
0.00	0.00	0.00	65.41	57.13	16.82	34.97	16.44
0.00	95.55	104.42	89.07	109.80	152.05	349.70	61.79
49.50	49.85	26.62	0.00	21.42	25.07	38.30	0.00
0.00	47.78	106.46	37.58	98.19	176.79	136.55	0.00
1127.85	1865.34	1500.74	1326.37	0.00	2410.40	2156.48	0.00
0.00	0.00	0.00	0.00	21.42	0.00	21.65	6.24
685.90	33.24	55.28	761.30	19.64	89.38	98.25	304.99
84.85	0.00	0.00	0.00	52.67	0.00	23.31	0.00
0.00	24.93	28.66	0.00	0.00	18.47	71.61	7.37
0.00	0.00	0.00	61.24	0.00	20.45	76.60	0.00
770.76	105.94	120.80	765.48	433.84	152.38	91.59	322.57
0.00	0.00	0.00	0.00	8.03	0.00	0.00	20.41
0.00	64.39	45.04	36.19	0.00	6.60	16.65	0.00

cow5_D3	cow5_D10	cow5_D21	cow6_D0	cow6_D3	cow6_D10	cow6_D21
16.17	11.91	15.05	10.60	9.02	14.10	17.76
0.00	3.53	0.72	3.03	2.82	2.35	7.40
148.03	119.14	214.25	53.00	122.39	51.71	68.07
87884.97	134384.96	105643.10	86414.84	163635.91	105247.11	170092.12
44000.33	44963.84	42424.91	46373.20	48924.73	109744.14	203632.72
16143.18	19120.38	9249.82	25697.21	23387.92	20256.25	34103.66
2965.66	3335.95	2265.70	1938.23	4038.33	5248.37	6156.00
603.33	1008.73	980.23	837.88	1883.24	1733.00	2973.67
19258.73	35521.69	59819.64	12180.07	45002.02	20503.82	27549.59
17602.37	24792.38	51739.93	9636.14	26789.48	12964.63	14152.89
6237.33	7471.47	11099.21	3998.11	5675.10	6506.60	6349.12
130.62	92.67	59.47	130.73	154.54	92.45	187.94
1081.02	1254.51	2812.42	722.80	1289.33	544.50	432.10
1.87	3.53	0.72	0.00	0.56	3.92	7.40
4.98	5.30	0.00	7.07	5.08	0.00	0.00
3297.18	3856.64	0.00	3291.96	4782.83	11818.43	0.00
32.97	45.45	20.78	62.59	51.89	43.87	28.12
5.60	18.09	12.18	2.52	9.59	10.18	2.96
0.62	6.18	4.30	0.50	1.69	1.57	1.48
4561.07	4196.41	4458.31	2434.40	6120.10	5338.47	11227.31
23.64	17.65	42.28	7.57	22.56	39.17	65.11
473.34	593.50	251.51	399.26	525.66	526.48	688.11
500.08	312.86	514.48	219.57	584.88	983.24	816.11
15.55	4.41	49.44	4.04	18.05	18.80	5.18
46.03	52.95	118.95	56.53	81.78	43.09	85.09
19.90	6.62	10.03	12.11	7.33	22.72	13.32
8.71	7.94	10.75	3.03	3.38	3.92	9.62
6.84	6.18	2.15	9.09	7.33	2.35	5.18
23.64	10.15	35.11	6.06	9.59	18.02	223.45
31.72	10.15	2.87	18.68	15.79	1.57	0.74
21.77	21.62	41.56	15.65	14.66	159.04	69.55
2.49	11.47	0.00	10.60	6.77	5.48	5.92
3.73	4.85	11.46	2.02	0.56	3.13	3.70
52.87	36.18	70.94	31.29	54.15	10.97	16.28
1.24	3.97	5.02	2.02	5.08	4.70	12.58
51.63	61.34	113.93	37.86	73.32	192.73	142.06
1.87	1.32	5.02	0.50	0.00	5.48	1.48
0.00	0.44	0.00	1.01	1.13	0.78	0.74
1.24	2.21	12.18	1.51	1.13	3.13	2.22
4.35	3.09	6.45	3.53	3.38	4.70	8.88
0.00	1.32	0.00	3.03	1.69	3.13	1.48
0.62	0.00	0.72	0.00	0.00	3.92	0.74
1.24	2.21	0.00	0.50	1.69	0.78	0.00
0.00	0.44	0.72	0.00	3.38	3.13	0.00
0.62	1.32	0.72	2.02	1.13	3.13	0.74
0.62	0.44	0.72	1.01	1.69	0.00	0.74

0.00	3.09	0.00	3.03	1.13	1.57	1.48
0.62	0.00	0.72	0.00	0.00	5.48	13.32
0.62	1.32	1.43	0.00	1.13	9.40	10.36
0.62	1.32	0.72	4.04	2.26	2.35	2.22
27.99	19.86	2.15	18.17	6.77	3.13	4.44
7786.09	3887.09	11798.55	2744.82	7650.83	470832.67	76355.89
3.73	6.62	10.03	4.54	1.69	7.05	5.92
965.95	1067.86	472.92	665.26	1222.22	268.73	2209.35
1322.35	1448.23	559.62	1325.47	1974.04	969.13	5245.18
157.99	139.88	89.57	147.89	127.47	48.57	121.34
46.65	25.15	15.76	25.74	27.07	20.37	16.28
36.08	36.18	18.63	6.06	6.20	10.97	25.90
27.37	43.24	55.17	42.40	37.79	43.09	60.67
538.64	340.66	74.52	421.46	631.69	241.30	458.00
9.33	7.50	42.99	10.09	5.64	40.74	36.26
1.87	1.32	10.03	2.02	2.82	2.35	5.92
18.66	8.83	9.32	12.62	11.84	7.05	11.10
461.52	408.61	117.51	534.02	311.90	337.67	389.93
1.87	3.09	17.91	1.01	5.08	8.62	11.10
4.98	4.85	42.28	6.06	14.10	287.53	278.94
31.72	15.00	68.07	11.61	18.61	18.02	9.62
29.23	12.80	14.33	25.74	18.61	11.75	22.94
30.48	19.86	35.83	27.26	71.63	24.29	19.24
1.24	3.09	5.73	0.00	0.56	2.35	1.48
6.84	5.74	43.71	0.00	2.82	13.32	10.36
6.22	7.94	14.33	6.56	9.59	26.64	59.93
1510.82	1451.31	3130.56	1192.72	1990.97	2163.12	2983.29
3776.11	1950.82	2573.81	1623.27	2910.87	1191.64	1405.82
299.80	116.49	1889.52	67.13	225.61	0.00	0.00
225.78	229.02	500.15	187.77	329.38	147.29	127.26
18.04	8.83	11.46	9.09	12.41	4.70	1.48
159.85	92.67	36.54	163.03	128.03	89.31	137.62
36.70	22.06	270.85	9.59	9.59	59.54	47.35
453.43	339.33	430.64	269.03	353.64	348.64	933.76
18.66	6.62	48.01	6.56	21.43	49.36	112.47
814323.60	758072.20	591511.09	1055231.25	693879.58	328276.61	231174.91
2855.56	2995.30	3443.69	3111.77	2805.40	1429.02	1066.20
24.26	15.89	22.21	19.18	31.02	11.75	17.02
69.04	36.62	83.84	53.00	34.40	37.61	51.79
113.20	120.02	160.51	94.89	130.29	114.38	230.85
16600.96	17880.43	8054.63	18208.27	11582.57	7870.60	8759.73
589.03	564.38	781.03	342.22	650.31	680.04	896.76
195.93	148.71	259.39	176.66	138.18	532.75	740.64
17.42	13.24	90.28	8.08	20.87	38.39	44.39
52.87	53.83	195.62	23.72	71.63	72.86	61.41
330.28	326.53	940.82	140.82	350.25	347.07	657.03
114.45	119.14	351.10	45.93	157.92	115.17	214.57
6.84	6.62	7.88	7.57	6.77	1.57	4.44

247.55	177.39	326.74	98.93	219.96	307.11	532.73
1648.90	1324.23	1327.75	910.06	1587.70	1924.95	2340.32
451.57	292.12	252.94	206.44	343.48	657.32	843.49
1.87	2.65	5.02	1.01	3.38	3.92	13.32
20.53	4.85	13.61	10.60	15.23	31.34	62.89
3070.77	3977.11	3579.83	2913.91	4456.83	3548.27	5505.63
395.59	574.52	788.19	527.97	803.15	839.08	1548.62
1063.61	1350.27	1970.49	602.67	1104.34	785.81	703.65
0.62	0.88	0.72	62.08	15.23	30.55	105.07
311.00	341.54	791.78	112.56	434.29	832.03	1311.85
8482.73	6006.48	8450.16	4054.13	9492.90	6019.29	6531.87
4.35	4.85	24.36	4.04	4.51	15.67	25.16
29.86	12.80	6.45	10.09	18.05	3.92	26.64
9298.78	7287.46	5033.69	6010.54	8869.10	5105.00	6495.62
369.46	608.06	525.22	286.19	456.85	381.54	533.47
12.44	7.06	0.00	1.51	2.82	0.00	0.00
586.54	358.75	0.00	560.27	353.07	908.03	1043.26
1.87	0.88	8.60	0.00	0.00	8.62	17.02
371.33	221.51	269.42	208.46	373.94	369.01	796.88
1.87	1.32	5.02	0.50	1.13	0.78	4.44
148.66	134.59	113.93	212.50	375.63	416.80	654.08
11.82	4.85	11.46	6.56	8.46	10.97	24.42
215.21	136.35	106.76	118.62	148.90	43.87	102.85
37.94	30.89	32.96	9.09	31.58	14.10	14.80
116.31	81.63	242.91	49.97	117.31	29.77	59.93
31765.75	22730.79	25164.89	18780.66	31455.56	13723.01	15767.36
54638.26	59053.82	48643.76	40387.39	66166.03	37840.13	72114.76
75542.78	55273.52	23657.29	47731.98	60368.55	43816.33	93381.82
15.55	24.27	22.21	8.58	29.33	34.47	102.85
411.14	285.06	667.82	126.19	399.89	372.14	683.67
24.88	30.01	53.02	12.62	36.10	47.79	73.99
72.15	50.30	139.73	34.32	52.45	42.31	65.85
5.60	1.77	7.17	0.00	2.82	0.78	0.74
3.11	6.18	26.51	2.02	2.26	32.12	27.38
158.61	157.53	219.26	204.42	409.47	147.29	133.92
60118.63	110438.05	258857.31	35186.47	83729.39	152313.22	236903.25
3.73	2.65	14.33	1.01	1.69	8.62	17.76
466.49	283.29	1155.78	218.56	428.65	1413.35	2007.36
79.61	67.07	144.02	71.67	50.20	317.30	384.75
241.33	30.89	238.61	19.18	151.72	101.07	13.32
440.99	241.81	0.00	240.76	261.14	0.00	0.00
5.60	6.18	14.33	6.06	9.59	19.59	57.71
8212.78	5826.44	11126.44	4369.60	8043.95	21609.28	29428.95
0.00	0.00	1.43	0.00	1.13	2.35	2.22
0.00	0.44	0.72	0.50	0.56	18.80	1.48
136.22	117.38	129.69	97.92	151.16	211.53	213.09
1.24	0.44	2.15	0.50	0.56	0.78	0.74
0.62	6.18	10.03	5.55	0.56	12.54	0.00

11.82	15.00	0.00	0.00	14.10	25.07	22.20
62.20	70.60	77.39	23.22	81.22	35.26	14.80
3.11	3.53	8.60	2.02	2.82	4.70	4.44
33.59	18.53	48.01	18.68	45.69	32.12	17.02
11.82	3.97	11.46	0.50	0.00	0.78	0.00
1.24	3.53	18.63	0.50	3.38	1.57	0.74
54.74	25.59	110.35	37.35	64.30	171.58	54.01
2531.51	2782.61	3990.41	1880.69	2529.60	2621.44	1907.47
375.06	399.78	0.00	0.00	389.17	559.39	182.02
12.44	16.77	0.00	5.05	20.30	32.91	19.24
42.92	51.19	41.56	38.87	51.89	43.09	44.39
2.49	12.80	7.17	11.10	16.92	14.10	0.00
6.84	15.00	6.45	11.61	0.00	14.89	12.58
9.33	12.36	12.90	8.58	10.72	16.45	8.88
8.09	9.71	7.17	7.07	8.46	12.54	7.40
63.44	41.48	0.00	30.28	65.99	80.70	41.43
1.87	0.88	0.00	0.50	1.13	0.78	1.48
63.44	41.48	103.18	30.28	65.99	80.70	41.43
120.04	122.67	169.10	60.06	117.88	186.46	0.00
6.22	1.77	15.05	2.52	3.38	5.48	4.44
26.12	21.18	10.03	22.21	23.69	10.97	10.36
0.00	0.44	0.00	6.06	17.48	17.24	5.18
4.35	12.36	12.90	9.09	9.02	14.89	6.66
1.87	2.21	5.02	0.00	2.82	3.13	2.96
9.33	5.74	32.24	7.07	9.59	10.18	2.22
2.49	1.32	7.17	5.05	1.13	4.70	2.96
0.00	0.00	14.33	2.02	2.26	1.57	1.48
6.84	14.12	30.09	7.07	11.28	7.83	5.18
100.76	82.96	168.39	58.05	114.49	68.16	48.09
3.11	2.21	11.46	0.50	0.00	3.92	0.00
1.87	3.09	1.43	3.53	6.77	2.35	1.48
3.73	13.68	6.45	11.61	17.48	16.45	2.22
11.20	9.71	12.18	7.57	5.08	2.35	0.74
6.84	5.30	7.88	0.00	0.56	0.00	0.00
5.60	7.50	24.36	4.54	9.59	3.13	5.18
0.62	3.09	1.43	0.50	2.82	3.13	0.00
2.49	7.94	12.18	3.53	3.95	9.40	1.48
0.00	0.00	0.00	0.00	0.00	3.13	2.96
4.35	0.00	0.00	2.52	0.00	3.13	2.96
1.24	3.97	18.63	1.51	4.51	2.35	0.74
1.24	3.53	17.91	2.02	3.95	2.35	0.74
3.11	3.53	8.60	4.04	3.95	2.35	2.96
30.48	45.01	75.24	0.00	0.00	58.76	29.60
0.00	0.00	0.00	20.19	33.84	0.00	0.00
1.87	0.44	0.00	0.50	0.56	1.57	0.74
4.35	0.44	8.60	0.00	1.13	0.00	2.96
13.68	9.27	24.36	8.08	16.36	6.27	5.18
2.49	13.24	22.93	9.09	14.10	6.27	3.70

3277.28	3533.64	3680.15	1031.20	1434.28	353.34	350.71
6.84	1.77	0.72	1.01	0.00	3.13	2.96
0.00	18.97	0.00	16.66	24.25	18.02	9.62
7.46	3.53	4.30	2.02	0.56	9.40	11.84
7.46	3.53	4.30	3.03	0.56	9.40	11.84
6.84	2.65	2.15	2.52	1.69	8.62	3.70
4.35	2.21	2.15	2.52	2.82	3.92	3.70
1.87	2.21	0.00	2.52	3.95	1.57	5.92
1.24	0.00	0.00	1.51	0.00	1.57	0.00
1.24	0.44	0.72	2.02	0.00	1.57	0.74
38.56	28.24	38.69	17.16	34.40	11.75	5.92
0.62	0.00	5.02	0.00	0.00	0.00	0.00
17.42	22.95	21.50	26.25	19.74	18.02	28.86
6094.90	5514.91	10283.07	4032.43	7940.74	16877.21	13602.40
4593.41	3457.30	8367.04	2576.74	3176.52	7227.38	8400.87
4.98	0.00	2.15	2.52	3.38	0.78	0.74
54.11	31.33	0.00	47.95	31.58	24.29	24.42
4.35	0.44	2.15	3.53	0.56	1.57	2.96
146.79	72.81	14.33	69.66	98.14	39.17	49.57
1.24	0.44	1.43	2.02	1.13	5.48	8.14
2.49	0.88	2.15	0.50	6.20	5.48	5.18
4593.41	3457.30	8367.04	2576.74	3176.52	7227.38	8400.87
8.71	13.68	2.87	18.68	6.77	10.97	10.36
11.20	9.27	3.58	3.03	0.00	11.75	11.84
0.00	9.27	0.00	3.03	0.00	11.75	11.84
177.27	101.93	117.51	88.84	111.67	210.75	116.90
0.00	0.00	2.15	0.00	0.56	0.00	0.74
105.12	124.44	61.62	93.38	76.14	52.49	99.89
0.00	0.00	0.72	0.50	0.56	2.35	2.96
3.11	1.77	1.43	4.04	1.69	1.57	0.00
6466.23	5871.01	6399.42	3328.81	4609.11	2717.81	5300.67
105599.29	91701.57	100203.13	58464.43	93815.07	69450.23	115112.81
8240.77	7489.56	16949.76	4183.85	9398.15	5662.04	8577.71
71.53	93.99	199.91	71.17	85.17	120.65	97.67
3796.64	2806.43	8660.82	1801.44	3646.91	4949.09	7079.40
9454.28	12791.78	19541.48	6413.83	13791.24	7612.84	9475.95
344.58	552.90	242.91	312.94	401.01	390.94	1121.69
3.11	3.97	5.02	1.01	1.13	35.26	27.38
16.79	70.16	106.05	6.06	27.07	325.13	283.38
9.33	9.71	50.16	6.56	9.59	0.00	73.25
49.14	34.86	53.74	15.65	19.18	0.00	48.09
4.35	0.44	2.15	1.01	0.00	3.13	11.84
2029.56	1607.96	5563.93	715.23	1657.63	1835.64	2651.08
340.23	256.82	432.07	153.95	492.95	217.80	244.17
462.76	372.43	814.71	261.96	504.79	240.52	231.59
282.38	238.72	308.83	171.11	274.11	137.89	121.34
2221.13	2845.71	837.64	1990.22	2075.57	2238.33	3845.28
64876.24	56797.64	72636.40	34914.41	55067.39	26605.38	36392.19

19.90	22.50	16.48	30.79	17.48	21.94	33.30
1989.75	1847.13	1875.90	696.05	1927.80	669.85	1107.64
1471.63	2202.79	1223.13	911.57	1599.54	398.78	1803.89
33771.67	25692.55	14755.00	23591.91	29338.25	12020.56	25577.75
817.92	1060.80	677.85	654.15	983.64	660.45	1017.37
1768.94	1432.78	2209.81	935.80	1673.43	885.31	1203.08
2154.58	2572.12	932.22	1080.16	1739.98	366.66	1462.79
0.00	1.77	1.43	2.02	5.08	25.07	170.92
259.99	183.57	364.72	107.01	319.23	85.40	132.44
10106.12	7486.03	3116.95	7854.88	6328.79	16560.69	14909.81
6.84	2.65	0.72	1.01	6.20	45.44	37.74
17.42	14.56	17.91	13.63	18.61	20.37	28.86
15.55	11.91	69.50	7.07	15.23	65.81	31.82
84.59	59.57	44.43	57.54	60.91	31.34	66.59
3.73	10.15	27.23	7.57	6.20	32.12	36.26
18.04	34.86	30.09	20.69	35.53	10.97	42.17
80.24	86.93	99.60	63.09	55.27	124.57	136.88
4.35	7.50	7.88	1.01	5.08	4.70	7.40
18.66	6.18	7.17	20.19	21.43	15.67	11.10
486.40	425.38	455.00	288.21	617.03	67.38	299.66
694.76	619.09	1372.89	381.08	1042.86	550.77	793.18
190.95	236.08	347.52	113.06	174.28	207.62	256.01
12.44	3.09	9.32	5.55	9.02	10.97	20.72
278.03	226.81	170.54	495.16	262.83	492.79	1158.69
20.53	15.89	10.03	22.71	15.23	26.64	83.61
32.34	19.42	83.12	12.11	21.43	9.40	6.66
205.26	182.24	285.90	174.64	181.61	171.58	194.59
23.64	40.15	174.84	12.11	49.07	20.37	31.08
3.73	4.85	5.73	0.50	2.82	12.54	2.22
5670.70	5711.27	446.40	4652.26	5135.90	1231.59	870.13
10.57	4.41	27.95	10.09	9.02	10.97	14.06
4.35	2.21	12.90	0.50	3.95	2.35	0.74
92.68	78.99	70.94	50.98	124.65	83.83	70.29
80.24	103.26	173.40	48.46	99.83	57.19	71.03
180.38	140.76	41.56	119.63	158.49	8.62	2.96
180.38	140.76	41.56	119.63	158.49	10.18	1.48
506.30	187.98	642.02	240.26	573.04	155.12	81.39
612.04	804.86	2378.91	217.55	827.41	263.24	244.91
3.11	2.21	1.43	1.01	4.51	2.35	2.22
6962.58	5005.25	1543.43	5912.62	5211.48	3555.32	4116.83
4671.78	5288.98	25296.02	3432.28	3426.38	13439.40	5993.96
87.08	87.81	333.91	37.35	92.50	402.70	312.24
778.11	791.19	4014.06	646.58	640.72	2766.38	917.48
20.53	18.53	177.70	39.88	49.07	337.67	174.62
32.34	18.53	9.32	5.05	0.00	3.13	5.92
15.55	14.12	7.17	1.01	0.56	4.70	5.18
18.66	13.68	2.87	1.51	2.82	1.57	13.32
5.60	2.65	2.15	1.01	1.69	0.00	12.58

31.72	14.56	17.91	2.02	3.38	5.48	11.10
138.08	101.93	139.73	77.23	153.41	114.38	189.42
905.62	898.85	688.60	844.95	838.12	632.25	1593.01
7606.34	4338.94	4494.86	8331.37	3379.56	10003.95	2494.96
0.62	0.44	2.15	2.02	1.13	1.57	3.70
0.62	0.44	77.39	2.02	1.13	15.67	8.88
90.19	56.48	132.56	39.88	85.17	120.65	102.85
268.08	213.13	152.62	183.22	203.04	237.39	265.63
21.77	20.30	26.51	15.65	20.30	12.54	5.92
577.83	377.28	307.40	338.18	683.02	348.64	509.79
0.00	0.00	1.43	0.00	0.00	0.78	0.00
3.11	3.97	22.93	2.02	0.56	22.72	13.32
2.49	6.62	128.98	3.53	3.38	18.80	9.62
32.34	95.75	103.18	41.39	38.35	83.83	56.23
3.11	2.21	5.02	0.00	1.13	1.57	12.58
0.00	1.32	5.02	0.00	0.56	13.32	15.54
1.24	4.41	15.76	5.55	1.13	20.37	35.52
243.20	239.61	171.97	136.79	212.63	164.53	290.04
37.94	76.78	90.28	33.82	27.64	61.11	82.87
0.62	0.88	2.15	2.52	0.00	2.35	1.48
0.62	5.30	3.58	2.52	3.38	6.27	12.58
6.84	8.38	3.58	1.01	2.82	4.70	5.18
4.35	2.21	1.43	1.51	0.56	5.48	19.24
120.67	75.46	85.27	105.49	182.18	113.60	81.39
283.01	379.93	391.23	261.46	466.44	253.84	355.15
77.13	53.83	93.15	45.43	119.57	56.41	54.75
13.68	11.03	12.90	11.61	21.43	13.32	51.79
0.62	0.88	8.60	0.50	0.00	11.75	5.18
2.49	4.85	6.45	1.51	7.90	18.80	28.12
19.28	13.68	26.51	8.58	19.18	27.42	105.07
2645.95	2313.10	1900.98	3100.67	3550.46	1503.45	1514.58
0.00	0.00	24.36	0.00	0.00	7.83	7.40
2104.82	2418.12	2682.73	806.08	1507.61	484.18	331.48
3.11	4.85	8.60	3.53	5.08	1.57	2.96
47.27	47.22	90.28	21.70	78.96	39.96	51.79
71.53	97.96	65.21	130.22	128.03	96.37	151.68
4.98	2.21	14.33	0.50	3.38	0.78	15.54
5.60	2.65	0.72	11.61	7.33	2.35	11.84
16.79	4.41	12.18	5.55	9.59	11.75	6.66
41.05	37.95	54.46	29.78	49.07	33.69	34.04
57.22	40.15	17.20	39.37	36.66	35.26	34.78
0.62	0.44	10.03	0.00	0.56	10.18	0.74
1258.29	1053.30	1259.68	642.54	617.59	471.64	605.98
10.57	9.27	11.46	5.55	9.59	27.42	24.42
125.02	174.74	167.67	75.71	153.98	68.94	44.39
9.95	6.62	17.20	7.07	5.64	20.37	13.32
181.00	225.49	435.66	136.28	201.92	130.05	264.89
313.48	348.16	786.76	337.17	240.83	742.72	275.24

1799.42	1921.26	1631.56	1628.82	2749.00	1652.31	1811.29
100.14	73.25	89.57	29.28	80.09	20.37	372.17
6.84	19.42	20.78	27.76	9.59	8.62	18.50
140.57	185.33	107.48	141.83	257.19	57.98	59.19
873.90	1295.99	1275.44	866.15	803.72	651.84	655.56
0.62	2.21	1.43	2.52	9.59	3.13	14.80
8.71	11.03	2.87	14.64	11.84	3.13	8.88
42.92	41.48	23.65	57.54	50.20	36.04	151.68
113.82	102.81	119.66	135.78	84.60	726.26	554.93
96.41	118.26	160.51	80.76	111.11	69.73	117.64
13.06	22.50	7.88	3.53	9.59	0.00	7.40
23.64	40.60	7.17	13.12	15.79	28.20	71.03
77.13	67.95	94.58	71.17	101.52	39.17	32.56
172.29	131.50	59.47	175.65	270.73	91.66	121.34
2.49	3.53	3.58	4.04	3.38	5.48	3.70
9.95	11.91	7.88	4.04	6.77	6.27	5.92
0.00	0.44	1.43	0.00	0.00	8.62	2.96
2.49	3.53	0.00	1.01	3.95	0.00	0.74
1.87	9.71	1.43	8.08	1.13	1.57	0.74
8.71	11.03	39.41	5.55	11.84	55.63	66.59
8.71	18.53	54.46	13.12	16.36	74.43	34.04
431.66	406.40	222.13	102.97	216.58	35.26	71.77
7523.61	4995.10	4497.72	3709.90	6520.55	1982.14	3120.92
85.83	56.04	60.91	77.23	62.61	54.06	70.29
623.86	566.58	1471.06	244.80	480.54	452.05	550.49
7.46	8.38	19.35	6.56	15.23	14.89	20.72
8.09	18.53	18.63	3.53	10.72	4.70	17.02
1.24	2.65	9.32	2.02	3.95	1.57	0.74
137.46	112.52	198.48	99.94	129.16	107.33	206.43
1161.88	1385.13	2064.35	797.00	2259.44	1215.14	1445.77
4.35	2.21	2.15	3.53	3.38	3.13	4.44
23.64	23.39	101.75	11.10	22.00	14.10	24.42
57841.51	46459.73	26742.00	60256.28	68408.55	48748.97	90136.60
1061.12	1370.12	702.21	833.34	901.29	886.87	2951.48
21.15	26.03	0.00	13.63	19.74	26.64	28.86
9.33	8.83	0.00	10.09	12.97	33.69	27.38
1.87	15.44	0.00	12.11	19.74	0.00	0.00
6.22	8.83	0.00	9.59	6.77	0.00	0.00
32.34	38.83	10.75	0.00	77.27	16.45	0.00
27.99	33.09	30.81	26.25	31.58	23.50	16.28
0.00	0.00	133.99	0.00	0.00	0.00	210.13
255.02	829.13	2449.85	0.00	500.28	7723.31	4234.47
0.00	0.44	1.43	0.00	1.69	2.35	1.48
95.16	75.90	38.69	133.25	140.44	91.66	65.11
12.44	12.36	42.99	8.08	12.41	13.32	18.50
119.42	118.26	108.20	0.00	156.80	0.00	0.00
0.00	0.00	88.85	0.00	0.00	51.71	17.76
90.81	75.01	25.08	98.43	50.20	0.00	0.00

0.00	0.00	2.87	0.00	0.00	23.50	61.41
0.00	3.53	8.60	0.00	0.00	13.32	8.88
121.91	104.14	0.00	0.00	116.75	0.00	758.40
14.31	15.44	42.99	0.00	19.18	83.05	49.57
103.87	90.90	344.66	100.95	66.55	521.00	78.43
0.00	0.00	303.81	0.00	9.02	114.38	20.72
7.46	8.38	19.35	6.56	15.23	14.89	20.72
1102.79	1440.28	2019.21	0.00	1173.71	822.63	740.64
6.22	3.09	1.43	3.53	5.64	8.62	2.96
468.36	6.18	13.61	273.57	577.55	1376.53	44.39
11.82	0.00	57.32	0.00	14.66	0.00	0.00
8.71	13.68	31.53	0.00	10.72	11.75	0.00
0.00	29.12	0.00	23.72	47.38	32.91	14.80
220.81	209.16	216.40	223.10	291.03	256.19	345.54
12.44	14.12	0.00	16.66	12.41	0.00	11.10
0.00	0.00	0.00	0.00	0.00	0.00	0.00
